# Design, Synthesis, and Anti-PVY Biological Activity of 1,3,5-Triazine Derivatives Containing Piperazine Structure

**DOI:** 10.3390/ijms24098280

**Published:** 2023-05-05

**Authors:** Lian Bai, Chunle Wei, Jian Zhang, Runjiang Song

**Affiliations:** Center for R&D of Fine Chemicals of Guizhou University, Key Laboratory of Green Pesticide and Agricultural Bioengineering, Ministry of Education, National Key Laboratory of Green Pesticide, Guiyang 550025, China; 15885174719@163.com (L.B.); weichle@163.com (C.W.)

**Keywords:** 1,3,5-triazine derivatives, piperazine, 3D-QSAR, potato virus Y

## Abstract

In this study, a commercial agent with antivirus activity and moroxydine hydrochloride were employed to perform a lead optimization. A series of 1,3,5-triazine derivatives with piperazine structures were devised and synthesized, and an evaluation of their anti-potato virus Y (PVY) activity revealed that several of the target compounds possessed potent anti-PVY activity. The synthesis of compound **C35** was directed by a 3D-quantitative structure–activity relationship that used the compound’s structural parameters. The assessment of the anti-PVY activity of compound **C35** revealed that its curative, protective, and inactivation activities (53.3 ± 2.5%, 56.9 ± 1.5%, and 85.8 ± 4.4%, respectively) were comparable to the positive control of ningnanmycin (49.1 ± 2.4%, 50.7 ± 4.1%, and 82.3 ± 6.4%) and were superior to moroxydine hydrochloride (36.7 ± 2.7%, 31.4 ± 2.0%, and 57.1 ± 1.8%). In addition, molecular docking demonstrated that **C35** can form hydrogen bonds with glutamic acid at position 150 (GLU 150) of PVY CP, providing a partial theoretical basis for the antiviral activity of the target compounds.

## 1. Introduction

Potato virus Y (PVY) is an RNA virus that was initially discovered in 1931 by Smith [1]. It is a constituent of the genus *Potyvirus* and is one of the most prevalent plant viruses [2]. PVY can swiftly replicate and proliferate in plants and can infiltrate cash crops—including peppers, tobacco, and tomatoes—thus causing symptoms such as greenish, mottled, and deformed tobacco leaves in these crops, which has culminated in unfathomable economic losses for Chinese agriculture [3,4]. The potato Y virus capsid protein (PVY CP) is the paramount protein of PVY; it has curved linear particles without an envelope and is approximately 680–900 nm in length, 11–15 nm in diameter, and possesses a genome consisting of 10 kb of single-stranded RNA [5]. PVY CP is crucial for numerous physiological processes, including proliferating, transporting, and transmitting aphids [6,7,8]; therefore, PVY CP is the central protein to consider in the investigation of the antiviral mechanism of PVY. In addition, the irrational misuse of existing pesticides and the inefficacy and environmental impacts of certain pharmaceuticals have contributed to an incremental proliferation of PVY and to its global presence [9]. Therefore, it is essential that we discover a novel environmentally friendly and effective anti-PVY virus agent.

**Figure 1:**
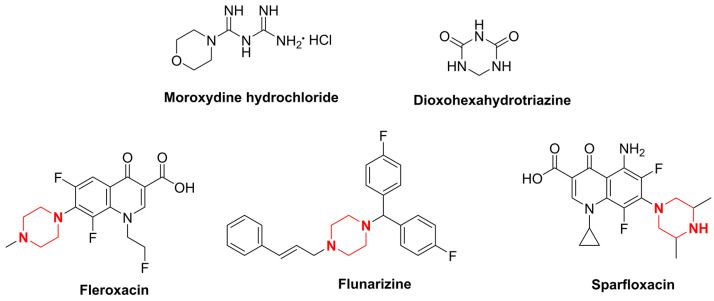
Chemical structure of moroxydine hydrochloride, dioxohexahydrotriazine, fleroxacin, flunarizine, and sparfloxacin.

Moroxydine hydrochloride is an antiviral agent with broad-spectrum antiviral activity [10] (Figure 1). It was introduced in the 20th century as an anti-influenza agent and is widely used to treat the infectious diseases that are caused by influenza and herpes viruses [11]. Since its introduction, most research has emphasized its use in the field of medicine [12]. In 1995, moroxydine hydrochloride was first registered as a national pesticide in China in combination with other pesticides for the control of tomato virus diseases. After 20 years of development, researchers discovered that moroxydine hydrochloride is effective at controlling tobacco mosaic virus, cucumber mosaic virus, and tomato virus [13]. However, after we evaluated the activity of moroxydine hydrochloride against PVY, we found that it was not as active as it should be. Therefore, we tried to optimize the structure of moroxydine hydrochloride. As they are lead compounds, heterocyclic structures containing nitrogen atoms, such as triazines [14], indoles [15], and quinolines [16]—which are themselves active structures with superb biological activity levels—have been emphasized in drug development. Triazine is a common lead structure in pesticide development, and it has been recognized in previous studies because it possesses a broad spectrum of biological activities, such as herbicidal [17,18,19] and antibacterial [20,21,22] activities, in addition to being a pharmacophore with decent antiviral [23] activity. Consequently, its physicochemical and pharmacological properties have intrigued a great deal of scrutiny from numerous researchers. Aside from a few researchers who have examined the effect of dioxohexahydrotriazine (DHT) (Figure 1) on the potato Y virus (PVY), the triazine structure found in the control version of the potato Y virus has not been studied nearly enough.

Piperazine has been utilized as a pharmacologically active moiety in the synthesis of pharmaceuticals. Due to its facile ability to form hydrogen and ionic bonds, it is frequently incorporated as a pivotal moiety in drug design for the purpose of modulating the biological activity of the parent scaffold [24]. The investigation of piperazine in the realm of pesticides primarily focuses on its antiviral [25,26,27,28], antibacterial [29,30,31,32,33], and insecticidal [34,35,36,37] properties. In the antiviral field, piperazine has spawned a number of commercial drugs with excellent antiviral activity, such as fleroxacin, flunarizine, and sparfloxacin (Figure 1).

**Figure 2:**
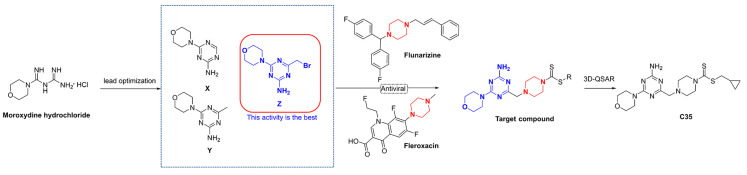
Design of the target compounds.

In this study, firstly, three intermediates containing triazine structures (**X**, **Y**, and **Z**) were synthesized via a lead optimization of moroxydine hydrochloride. Activity assays revealed that the intermediate **Z** possessed excellent live curative and protective properties against PVY (Figure 2). Secondly, by introducing a piperazine group into the structure, which also has antiviral activity, we anticipated that a small molecule with superior anti-PVY virus activity would be obtained. Additionally, a 3D quantitative structure–activity relationship (3D-QSAR) model was employed to screen the target compound **C35**, and the subsequent assessment of activity demonstrated that the anti-PVY virus activity and EC_50_ inactivation of **C35** were comparable to those of the control agent ningnanmycin (NNM). Finally, a concise depiction of the antiviral mechanism of **C35** through molecular docking analysis is also presented.

## 2. Results and Discussion

### 2.1. Chemistry

**Figure 3:**
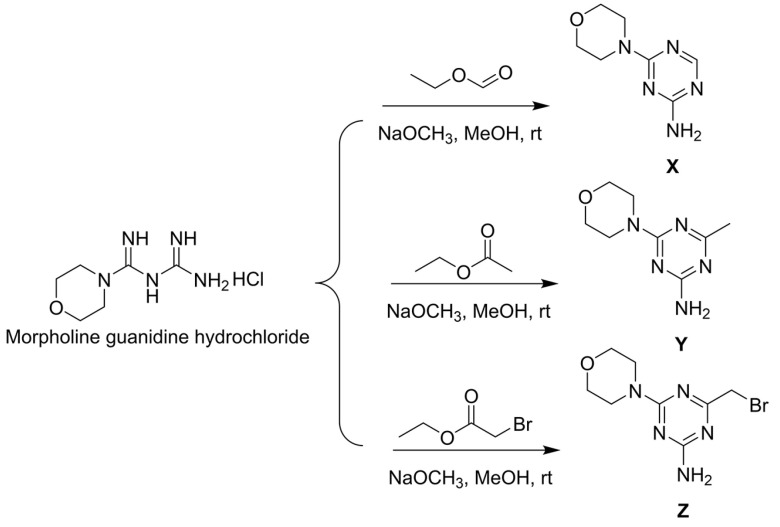
Synthesis route of intermediates **X**, **Y**, and **Z**.

**Figure 4:**
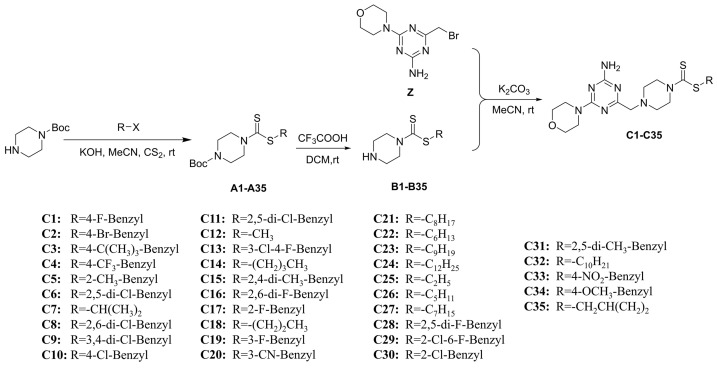
Synthesis route of target compounds **C1**–**C35**.

The synthesis of 6-morpholinyl−1,3,5-triazine intermediates **X**, **Y**, and **Z** is shown in Figure 3. The reaction of Boc-piperazine with SC_2_ and halohydrocarbon under alkaline conditions generated the intermediate Boc-piperazine dithiocarbonates **A1**–**A35**. The Boc-piperazine dithiocarbonate was deprotected in the presence of trifluoroacetic acid to generate the intermediate piperazine dithiocarbonates **B1**–**B35**. The final intermediate **Z** and the piperazine dithiocarbonates **B1**–**B35** underwent a nucleophilic reaction (Figure 4). All compounds were identified by ^1^H NMR, ^13^C NMR, ^19^F NMR, and HRMS (see Figures S1–S113 in the Supplementary Materials).

### 2.2. Anti-PVY Activity In Vivo

Table 1 demonstrates that among the all-target compounds, 16 exhibited superior anti-PVY curative activity compared to NNM (49.1%) and moroxydine hydrochloride (36.7%). The compounds **C8**, **C16**, **C18**, **C23**, and **C34** exhibited significant curative activity with their percentages of 56.8%, 53.3%, 55.3%, 56.1%, and 56.1%, respectively. Among the all-target compounds, 17 exhibited superior protective activity compared to the control agents NNM (50.7%) and moroxydine hydrochloride (31.4%), with **C5**, **C10**, **C19**, **C32**, and **C33** displaying remarkable protective activities of 63.6%, 60.1%, 60.0%, 65.5%, and 64.8%, respectively. The inactivation activity of the target compounds was evaluated and found to be terrific for the compounds **C9** and **C32**—with 82.7% and 80.9%, respectively—which were equivalent to NNM (82.3%) and were significantly superior to moroxydine hydrochloride (57.2%). Furthermore, owing to the exceptional passivation activity exhibited by these compounds, we have determined the EC_50_ values of all the compounds against PVY passivation based on the activity data presented in Table 1. Our findings indicate that the EC_50_ values of compounds **C9**, **C32**, and **C34** are comparable to that of NNM (72 μg/mL) with 108 μg/mL, 85 μg/mL, 83 μg/mL, and 89 μg/mL, respectively, which are much better than that of moroxydine hydrochloride (450 μg/mL).

### 2.3. 3D-QSAR Analysis

The comparative molecular field analysis (CoMFA) and comparative molecular similarity index analysis (CoMSIA) models were generated using SYBYL−2.0. The cross-validation coefficients (q^2^) and principal component scores (ONC) were used to compute the correlation coefficient (r^2^), standard error of estimate (SEE), and F values used in the CoMFA and the CoMSIA models, which are presented in Table 2. The q^2^ and r^2^ values of these models were found to be significant—>0.5 and >0.8, respectively—indicating their efficacy in predicting the framework of the target compounds. The residual analysis of the CoMFA and CoMSIA models’ experimental and predicted values is presented in Table 3.

**Figure 5:**
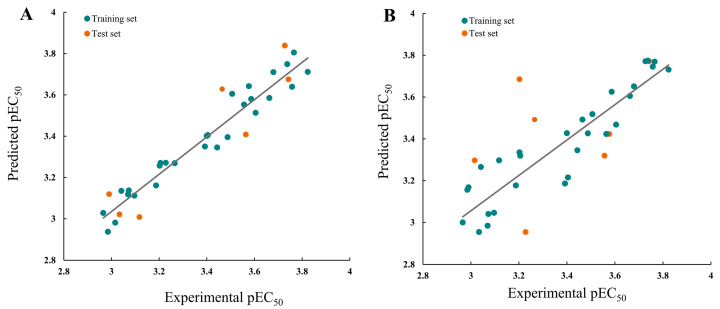
Plots of the experimental and predicted pEC_50_ for the (**A**) CoMFA and (**B**) CoMSIA models.

**Figure 6:**
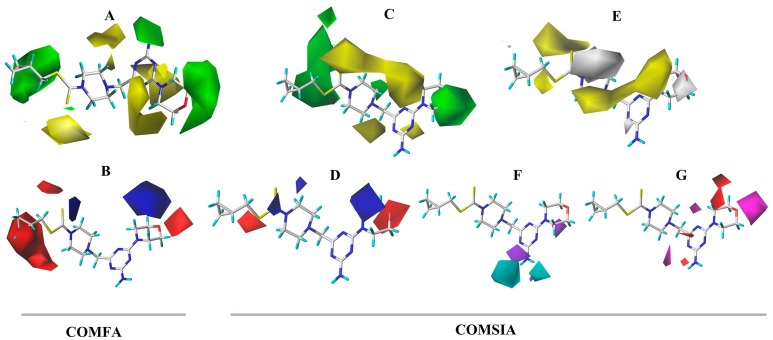
CoMFA three-dimensional isopotentials of (**A**) steric, (**B**) electrostatic fields and CoMSIA three-dimensional isopotentials of (**C**) steric, (**D**) electrostatic, (**E**) hydrophobic, (**F**) H-bond acceptor fields, and (**G**) H-bond receptor fields.

In the CoMFA model analysis, the CoMFA model linear equation (Figure 5A) has y = 0.9034x + 0.3248 (where x is the test activity and y is the predicted activity), q^2^ = 0.693, r^2^ = 0.920, ONC = 2, SEE = 0.080, and F = 184.422. Figure 6A depicts a 3D equipotential diagram of the stereo field, where green indicates that increasing the size of the group increases the activity of the compound and the yellow indicates that increasing the size of the group decreases the activity of the compound. The presence of a large green color block at the R substituent, as depicted in Figure 6A, indicates that the introduction of a small volume group here is advantageous for enhancing the activity of the target compound. The anti-PVY EC_50_ arises from the inactivation activity of the target compound when the F, Cl, and Br groups are sequentially introduced at the 4-position of the benzene ring if R is substituted with benzyl. For instance, **C1** (R = 4-F-benzyl; EC_50_ = 115 ± 5 μg/mL) > **C10** (R = 4-Cl-benzyl; EC_50_ = 133 ± 6 μg/mL) > **C2** (R = 4-Br-benzyl; EC_50_ = 566 ± 8 μg/mL). The anti-PVY EC_50_ arises from the inactivation activity of the target compound if R is aliphatically substituted. For example, **C18** (R = –C_3_H_7_; EC_50_ = 143 ± 7 μg/mL) > **C7** (R = –CH(CH_3_)_2_; EC_50_ = 161 ± 4 μg/mL). Figure 6B depicts a 3D equipotential diagram of the electrostatic field. The red blocks indicate that the introduction of an electron-withdrawing group at this position increases the activity of the target compound, while the blue blocks indicate that the introduction of an electron-donating group at this position increases the activity of the target compound. As depicted in Figure 6B, the R substituent is colored red, indicating that the incorporation of an electron-absorbing group here is advantageous for augmenting the activity of the target compound against PVY. For example, **C1** (R = 4-F-benzyl; EC_50_ = 115 ± 5 μg/mL) > **C33** (R = 4-NO_2_-benzyl, EC_50_ = 127 ± 5 μg/mL) > **C12** (R = –CH_3_, EC_50_ = 147 ± 6 μg/mL) ≈ **C25** (R = –C_2_H_5_, EC_50_ = 151 ± 10 μg/mL) ≈ **C18** (R = –C_3_H_7_, EC_50_ = 143 ± 7 μg/mL) > **C22** (R = –C_6_H_13_, EC_50_ = 449 ±4 μg/mL). Finally, the electrostatic field is responsible for 57.7% of the activity of the target compounds, while the steric field is responsible for 42.3%. These results indicate that both the electrostatic and steric fields contribute significantly to the enhancement of the activity of these compounds.

In the CoMSIA model analysis, the CoMSIA model exhibits an exemplary predictive capacity (Figure 5B), as evidenced by q^2^ = 0.632, r^2^ = 0.83, ONC = 2, SEE = 0.12, and F = 78.915; notably, the values of q^2^ > 0.5 and r^2^ > 0.8 indicate high prediction accuracies. The CoMSIA model’s steric and electrostatic fields are depicted in Figure 6C,D, which depict the 3D equipotential diagram. The color-coded activity analysis of each block aligns with that of the CoMFA model, thus requiring no further elaboration. The 3D isopotential plot of the hydrophobic field in the CoMSIA model, as depicted in Figure 6E, illustrates that the yellow regions correspond to the enhanced anti-PVY activity that occurs upon the addition of hydrophobic groups. Meanwhile, the white regions indicate the increased anti-PVY activity that occurs with the introduction of hydrophilic groups. Figure 6F,G illustrate the 3D equipotential diagram of the CoMSIA model hydrogen bond donor and acceptor fields, which express the significant contributions of the amino group on the triazine as a hydrogen bond donor and the oxygen atom on the morpholine and nitrogen atom on the triazine as hydrogen bond acceptors, which also enhance the anti-PVY activity of this target compound.

### 2.4. Synthesis and Activity Test of Target Compound ***C35***

**Figure 7:**
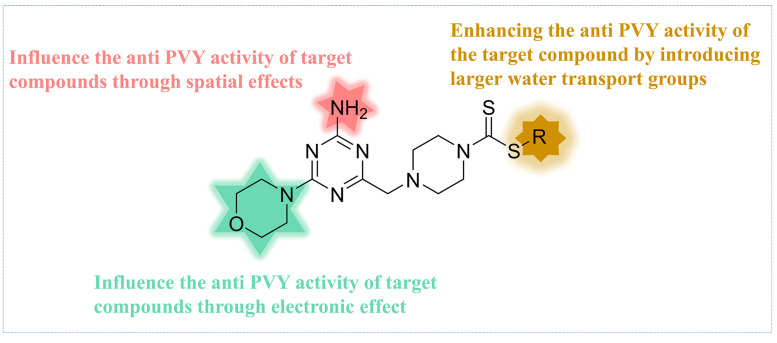
Analysis of structure–activity relationship of the anti-PVY activity.

**Figure 8:**
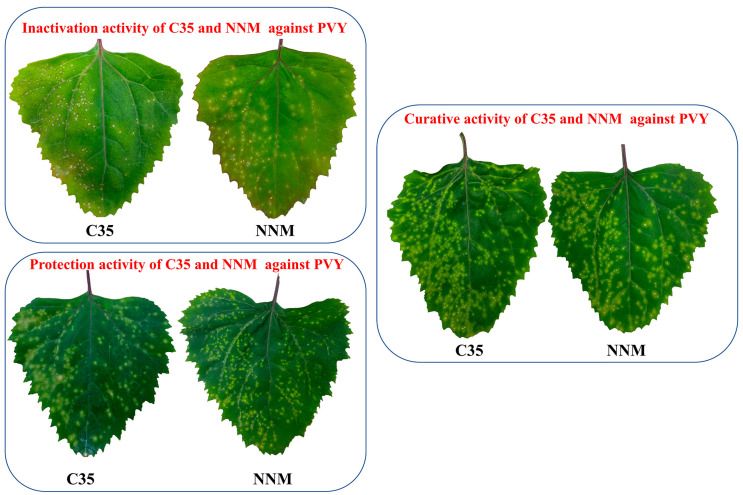
Curative, protective, and inactivation activity of **C35** against PVY at 500 μg/mL. NNM was used as a positive control.

Based on the analysis of the 3D-QSAR model results. Compound **C35** was synthesized with a cyclopropyl substituent at the R group to increase the hydrophobicity and spatial resistance while conserving the amino and morpholine groups on the triazine that substantially contribute to its anti-PVY activity (Figure 7). Additional research on the anti-PVY activity of **C35** revealed that its EC_50_ was 89 ± 6 μg/mL, which was substantially superior to that found in the control agent moroxydine hydrochloride (450 ± 3 μg/mL), and which was comparable to NNM (72 ± 6 μg/mL) (Figure 8).

### 2.5. Molecular Docking Analysis and MD Simulations

#### 2.5.1. Molecular Docking

**Figure 9:**
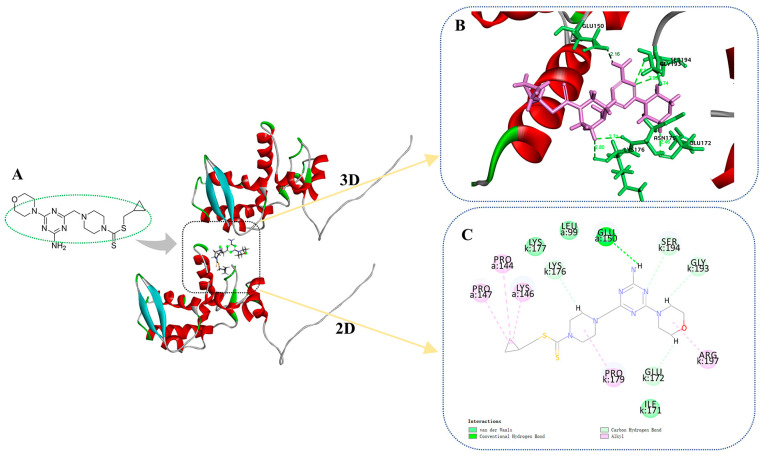
Molecular docking results of **C35** and PVY CP (**A**) molecular docking position of **C35**, (**B**) 3D diagram of molecular docking, (**C**) 2D diagram of molecular docking.

Docking compound **C35** into the pocket between the protein crystals (PDB: 6HXZ) can be achieved through molecular docking simulations. Based on Figure 9A–C, it can be observed that the target compound **C35** exhibits diverse interactions with PVY CP. Specifically, the amino group located on the triazine moiety of **C35** forms an intermolecular hydrogen bond with the GLU 150 of PVY CP at 2.16 Å. The glutamic acid at position 172 (GLU 172) and the lysine residue at position 176 (LYS 176) can participate in van der Waals interactions with morpholine, piperazine, and triazine through their side chains. Glycine at position 193 (GLY 193) and serine at position 194 (SER 194) also contribute to these interactions. Proline residues at positions 144 and 147 (PRO 144, PRO 147), as well as lysine residue at position 146 (LYS 146), proline residues at positions 179 (PRO 179), and arginine residue at position 197 (ARG 197) can form alkyl bonds with cyclopropyl, piperazine, and morpholine. This significantly enhances the interaction between the target compound **C35** and PVY CP, as well as provides a theoretical foundation for the anti-PVY capability of the compound.

#### 2.5.2. MD Simulations

**Figure 10:**
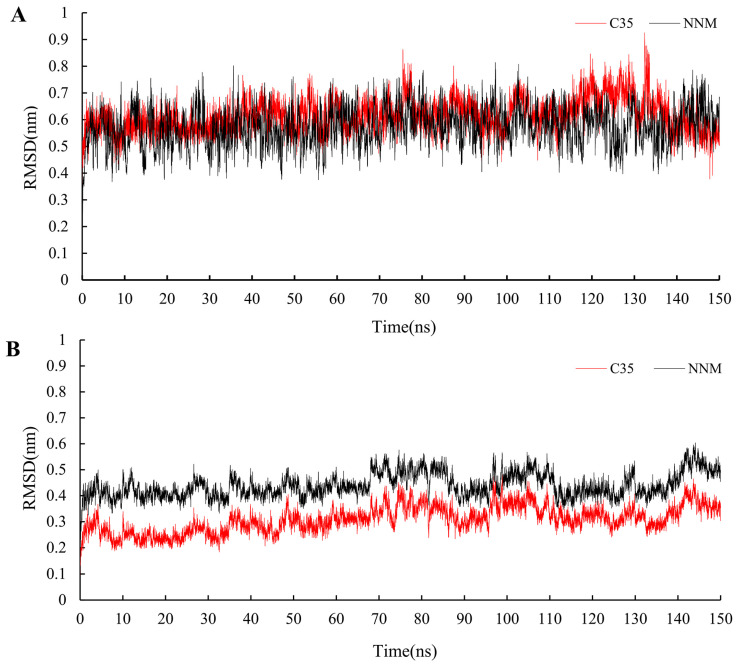
RMSD trajectories of the PVY–ligand complex (**A**) and ligand (**B**) during 150 ns simulations.

Using the results from the molecular docking experiments, simulations of molecular dynamics were conducted while monitoring the RMSD changes in the protein–ligand complexes. It was discovered that the compounds **C35** and NNM displayed similar motion trends to the protein complexes constructed with PVY proteins. The RMSD for 150 ns was found to be within 0.3 nm, indicating that the system is stable and that the results are representative (Figure 10). The binding energy of NNM (Gbind = −32.4 kcal/mol) was slightly greater than that of **C35** (Gbind = −27.1 kcal/mol) (Table 4), which was consistent with the results of the bioactivity test.

## 3. Materials and Methods

### 3.1. Instruments and Chemicals

All the chemical precursor materials used in the production of this article came from official sources. The melting points of all the target compounds were determined using an XGE X−4B micro melting point apparatus (Shanghai Yidian Physical Optics Instrument Co., Ltd., Shanghai, China). To identify the molecular structures, nuclear magnetic resonance NMR analyses were conducted using DMSO-*d*_6_ or CDCl_3_ as a solvent and a Bruker DPX instrument (Bruker, Karlsruhe, Germany) that was operated at a 400 or 500 MHz magnetic field strength. Then, a Thermo Scientific Q Exactive (Thermo Scientific, Waltham, MA, USA) was used and high-resolution mass spectrometry (HRMS) measurements were carried out. A 3D-QSAR model formulation was fulfilled utilizing SYBYL−2.0. In the experimental investigation, the software DISCOVERY STUDIO 4.5 (DS 4.5) was employed to execute the molecular docking of **C35** and PVY CP.

#### 3.1.1. General Procedures for Preparing Intermediates **X**, **Y**, and **Z**

According to the previously reported literature, the synthetic intermediates used were **X** [38], **Y** [39], and **Z** [40]. First, taking synthesis **Z** as an example, 1.0 eq. of moroxydine hydrochloride (10.0 g, 48.16 mmol) was dissolved in 40 mL of MeOH and kept at 37 °C for 2 days. Then, 1.3 eq. ethyl bromoacetate (6.94 mL, 62.60 mmol) was slowly added dropwise and the reaction system slowly changed from a colorless liquid to a white turbidity after 15 min of mixing. The reaction was monitored using thin-layer chromatography (TLC), and when the reaction was terminated, it was immediately filtered using a 60-mm Buchner funnel and the filter cake was washed with plenty of water and oven-dried in order to provide a white flocculent solid **Z**.

#### 3.1.2. General Procedures for Preparing Intermediates **A1**–**A35**

The synthesis of **A1** was used as an example for the synthesis of compounds **A1–A35** [41]. Firstly, 1.2 eq. KOH (542.21 mg, 9.66 mmol) was added to a stirring solution of acetonitrile containing 1.0 eq. Boc-piperazine (1.50 g, 8.05 mmol). After 15 min, 1.2 eq. of SC_2_ (579.33 μL, 9.66 mmol) was added, at which point the reaction system changed from a colorless liquid to a white turbidity. After 8 h, 0.5 mL of H_2_O was added dropwise to the system and the white turbidity slowly disappeared. After 30 min, 1.2 eq. of 4-fluorobenzyl chloride (1.16 mL, 9.66 mmol) was added and stirred for 2 h to produce a large white solid. The filter was drawn using a 60 mm Buchner funnel, and the cake was washed with a small amount of petroleum ether and filtered in the oven to give a white granular solid **A1**–**A35**.

#### 3.1.3. General Procedures for Preparing the Intermediates **B1**–**B35**

The synthesis of **B1** was used as an example for the synthesis of compounds **B1**–**B35** [41]. First, 1.0 eq. of **B1** (2.0 g, 4.94 mmol) was weighed in a 100 mL eggplant-shaped flask at 37 °C; it was then dissolved with 10 mL of DCM and held for 15 min. Then, 3.0 eq. of trifluoroacetic acid (1.10 mL, 14.82 mmol) was added and stirred for 3 h. The reaction was monitored using TLC, and when the reaction was terminated, the reaction mixture was poured into water, extracted with dichloromethane, concentrated to obtain the crude product, dried under infrared to obtain a white solid **B1**–**B35**, and the crude product was directly used as the raw material for the next reaction step without further purification.

#### 3.1.4. General Procedures for the Preparation of the Title Compounds **C1**–**C35**

The synthesis of **C1** was used as an example for the synthesis of compounds **C1**–**C35**. First, 1.0 eq. of **B1** (200.00 mg, 739.69 *µ*mol), 1.1 eq. of **Z** (223.04 mg, 813.66 µmol), and 1.3 eq. of K_2_CO_3_ (153.34 mg, 1.11 mmol) were dissolved in acetonitrile in a 50 mL single-mouth flask and then stirred at 37 ℃. The reaction was monitored using TLC. When the reaction was terminated, the reaction mixture was poured into water, and a large amount of white solid was precipitated. This was finally extracted using a 60 mm Buchner funnel, and it was also dried and weighed to obtain the white lumpy solids of **C1**–**C35**.

### 3.2. Antiviral Activity Assay

The curative activity of compounds against PVY: A host, adopted as *Chenopodium amaranthcolor*, was used for the blight of PVY and the 200–300 mesh carborundum that was transmitted on its surface. The leaves and a small amount of the virus solution were diluted with 0.01 M of PBS and were then soaked in the leaves. After 1.5 h, the water was rinsed with the laminas and dried naturally, and 500 μg/mL of the compound solution was applied on the right side. A control solution was applied on the left side. After 5–7 d, the antiviral activities of the compounds were determined by critiquing the tissue splotch. Each agent was applied to three plants.

The protective activity of compounds against PVY: Then, 200–300 mesh carborundum, which was coated with 500 μg/mL of the mixture on the right-hand side and the control solution on the left-hand side and incubated in the glasshouse for 24 h, was sprinkled on the tissue; the virus solution was brushed onto the vane, rinsed with water after 1.5 h, dried naturally, and incubated in a glasshouse for 5–7 d; and the compound activity was assessed by observing tissue speckling. Each agent was applied to three plants.

The inactivating activity of compounds against PVY: An equal volume of the compound and 2 × virus solution was mixed for 30 min and plated on the right-hand side of the lamina. The left hand side of the leaves was inoculated with the virus solution for the control. This was then flushed with water after 1.5 h, the leaves were allowed to dry naturally, and were then incubated in a glass chamber. After 5–7 d, the compounds’ activity was assessed by observing the tissue speckles. Each agent was applied to three plants.

### 3.3. 3D-QSAR

Using SYBYL-2.0 software, the CoMFA and CoMSIA models were developed. Randomly partitioning all target compounds into a training set and a test set. The partial least-square method (PLS) was used to construct 3D-QSAR models of the structures and the anti-PVY passivation activities of the target compounds. First, the target compound structure was imported into the software for energy minimization. Next, the steric and electrostatic molecular fields of the molecule were evaluated by superimposing its three-dimensional structure. Finally, to obtain q^2^, ONC, r^2^, and SEE were used to predict the model’s predictive ability. Compound **C5** serves as the template molecule for both the CoMFA and CoMSIA models.

### 3.4. Molecular Docking and MD Simulations

We selected PVY CP as the subject of our investigation. The 3D protein structure of PVY CP used for docking was obtained from the Protein Data Bank (PDB, http://www.rcsb.org (accessed on 23 March 2022), under the accession number 6HXZ. Using DS 4.5 software, the hydrogenation and charge calculation of PVY CP were carried out. Chem3D 20.0 was used to assemble the structure of the compound **C35** and to optimize its energy, followed by the use of DS 4.5 for molecular coupling and visualization. Amber94 and TIP3P force fields were applied to the proteins and water, respectively, using the Amber18 procedure. Meanwhile, GAFF force fields were used for the organic small molecules, and the system was completed by adding sodium ions for the purpose of electrical neutralization. Then, the system was minimized using the steepest descent method for 1000 steps, and the conjugate gradient method was used for the next 2000 steps. After that, the system was simulated by molecular dynamics. This was performed according to the following steps: the system was heated from 20 K to 300 K for 30 ps under constant volume conditions; then, MD simulations were performed at 1 atm and 300 K via a relaxation process similar to the minimization; and finally, 150 ns molecular dynamics were performed for each system. Then, the binding-free energies (ΔG_bind_) of the sample compounds with PVY CP were computed using the molecular mechanics Poisson–Boltzmann surface area (MM-PBSA) method, and the contributions of residues to the ligand were determined using the deconstruct module.

## 4. Conclusions

This study centered on the lead optimization of the commercial antiviral drug moroxydine hydrochloride and incorporated the antiviral-active piperazine into the synthesized lead compound. Furthermore, this study designed and synthesized 34 1,3,5-triazine scaffold target compounds containing piperazine and morpholine structures. The preponderance of the compounds that were evaluated for their anti-PVY activity demonstrated their significant efficacy against PVY. The template molecule compound **C5** was utilized in order to develop a 3D-QSAR model that correlates, with its passivated EC_50_, with the structure of the compound. The target compound **C35** was simulated based on the above model and evaluated for its anti-PVY activity; the results revealed values of 53.3 ± 2.5%, 56.9 ± 1.5%, 85.8 ± 4.4%, and 89 ± 5 μg/mL for its curative, protection, inactivation, and EC_50_ values for inactivation activity, respectively. These are significantly higher results than those achieved by the control agent moroxydine hydrochloride (36.7 ± 2.7%, 31.4 ± 2.0%, 7.2 ± 1.8%, and 450 ± 3 μg/mL) and are comparable to the results achieved by NNM (49.1 ± 2.4%, 50.7 ± 4.1%, 82.3 ± 6.4%, and 72 ± 6 μg/mL). Finally, molecular docking was employed to provide a concise depiction of the potential amino acid binding sites between PVY CP and the target compound **C35**.

## Figures and Tables

**Table 1:** Antiviral Activities of the Compounds Against PVY.

Compd.	Curative Activity (%) *^a^*	Protective Activity (%) *^a^*	Inactivation Activity (%) *^a^*	EC_50_ for Inactivation Activity (μg/mL)
**X**	28.1 ± 1.8	36.8 ± 4.6	57.4 ± 3.0	-
**Y**	29.1 ± 4.5	14.2 ± 3.8	39.3 ± 3.4	-
**Z**	39.3 ± 2.2	42.8 ± 3.9	67.1 ± 2.1	-
**C1**	45.1 ± 3.5	34.2 ± 2.8	74.4 ± 3.2	115 ± 5
**C2**	40.4 ± 3.7	47.3 ± 1.4	46.1 ± 1.8	566 ± 8
**C3**	51.5 ± 2.2	46.8 ± 5.6	69.1 ± 3.8	109 ± 8
**C4**	48.4 ± 2.7	41.4 ± 6.4	47.9 ± 5.8	531 ± 9
**C5**	23.2 ± 3.6	63.6 ± 3.0	79.4 ± 5.9	86 ± 10
**C6**	36.5 ± 2.2	56.3 ± 2.2	55.5 ± 2.2	466 ± 8
**C7**	46.7 ± 3.4	56.3 ± 1.8	64.7 ± 4.9	161 ± 4
**C8**	56.8 ± 1.5	57.1 ± 3.7	67.4 ± 1.2	176 ± 2
**C9**	51.5 ± 2.1	56.1 ± 2.6	82.7 ± 4.5	108 ± 8
**C10**	40.0 ± 3.5	60.1 ± 4.1	71.4 ± 1.5	133 ± 6
**C11**	50.7 ± 3.0	42.8 ± 1.7	67.3 ± 2.7	156 ± 5
**C12**	43.8 ± 5.4	50.4 ± 2.5	70.0 ± 1.9	147 ± 6
**C13**	31.7 ± 4.6	41.1 ± 1.9	75.6 ± 4.3	91 ± 6
**C14**	38.2 ± 4.2	46.2 ± 3.1	78.0 ± 3.8	109 ± 6
**C15**	48.6 ± 5.2	42.9 ± 1.7	59.5 ± 5.6	361 ± 9
**C16**	53.3 ± 2.8	22.4 ± 3.4	67.3 ± 1.5	445 ± 7
**C17**	40.5 ± 3.7	48.7 ± 8.1	51.9 ± 4.7	301 ± 9
**C18**	55.3 ± 1.2	59.5 ± 5.6	73.0 ± 1.1	143 ± 7
**C19**	34.3 ± 2.1	60.0 ± 3.8	60.5 ± 2.4	371 ± 4
**C20**	40.6 ± 1.7	56.9 ± 4.8	60.0 ± 3.3	295 ± 6
**C21**	34.1 ± 4.5	52.3 ± 4.1	60.5 ± 1.1	291 ± 6
**C22**	53.9 ± 1.3	55.3 ± 1.4	48.4 ± 4.1	449 ± 4
**C23**	56.1 ± 5.1	46.8 ± 1.2	74.8 ± 3.3	150 ± 9
**C24**	51.7 ± 2.5	43.3 ± 3.5	62.0 ± 5.2	284 ± 9
**C25**	41.9 ± 1.9	46.5 ± 4.9	67.2 ± 8.1	151 ± 10
**C26**	52.0 ± 3.9	42.2 ± 7.3	77.8 ± 3.5	116 ± 5
**C27**	50.1 ± 4.8	49.7 ± 2.9	58.2 ± 3.9	386 ± 7
**C28**	53.7 ± 0.7	35.2 ± 2.5	56.2 ± 3.1	464 ± 7
**C29**	26.9 ± 3.3	49.5 ± 1.3	63.0 ± 1.2	294 ± 8
**C30**	36.1 ± 3.3	53.0 ± 1.8	53.5 ± 4.2	405 ± 7
**C31**	51.3 ± 2.4	52.7 ± 3.7	78.1 ± 4.4	99 ± 8
**C32**	56.4 ± 5.6	65.5 ± 5.6	80.9 ± 4.4	85 ± 6
**C33**	48.9 ± 4.6	64.8 ± 3.6	76.1 ± 2.2	127 ± 5
**C34**	56.1 ± 4.9	48.3 ± 2.2	79.8 ± 3.5	83 ± 5
**C35**	53.3 ± 2.5	56.9 ± 1.5	85.8 ± 4.4	89 ± 5
**Mor** *^b^*	36.7 ± 2.7	31.4 ± 2.0	57.2 ± 1.8	450 ± 3
**NNM** *^c^*	49.1 ± 2.4	50.7 ± 4.1	82.3 ± 6.4	72 ± 6

*^a^* The average of three replicates. *^b^* Moroxydine hydrochloride used as a control. *^c^* Ningnanmycin used as a control.

**Table 2:** Statistical Results of the CoMFA and CoMSIA Models.

Statistical Parameter	CoMFA	CoMSIA	Verification Standard
q^2^	0.693	0.632	>0.5
ONC	2	2	
r^2^	0.92	0.83	>0.8
SEE	0.08	0.12	
F value	184.422	78.915	
Steric	0.423	0.075	
Electrostatic	0.577	0.261	
Hydrophobic		0.162	
Hydrogen-bond acceptor		0.200	
Hydrogen-bond donor		0.302	

**Table 3:** Experimental and Predicted Results of pEC_50_ for the CoMFA and CoMSIA Models.

Compd.	Exp *^a^*	CoMFA		CoMSIA	
		Pred *^b^*	Res *^c^*	Pred *^b^*	Res *^c^*
**C1**	3.605	3.513	−0.092	3.468	−0.137
**C2**	2.966	3.028	0.062	3.000	0.034
**C3**	3.663	3.585	−0.078	3.605	−0.057
**C4**	2.985	2.937	−0.048	3.156	0.171
**C5** *^d^*	3.728	3.839	0.112	3.771	0.044
**C6**	3.042	3.135	0.093	3.265	0.223
**C7**	3.392	3.350	−0.042	3.186	−0.207
**C8** *^d^*	3.465	3.628	0.163	3.492	0.027
**C9**	3.824	3.711	−0.113	3.731	−0.093
**C10**	3.557	3.553	−0.003	3.341	−0.215
**C11**	3.487	3.395	−0.092	3.426	−0.061
**C12**	3.400	3.401	0.001	3.427	0.027
**C13**	3.737	3.749	0.012	3.774	0.036
**C14**	3.577	3.642	0.066	3.698	0.121
**C15** *^d^*	3.118	3.008	−0.109	3.297	0.180
**C16** *^d^*	3.034	3.021	−0.013	2.954	−0.080
**C17**	3.187	3.161	−0.026	3.177	−0.011
**C18**	3.444	3.345	−0.099	3.345	−0.099
**C19**	3.096	3.112	0.016	3.046	−0.050
**C20**	3.202	3.257	0.054	3.335	0.133
**C21**	3.206	3.270	0.064	3.319	0.113
**C22** *^d^*	2.990	3.119	0.129	3.168	0.178
**C23**	3.506	3.605	0.099	3.518	0.012
**C24**	3.265	3.269	0.003	3.358	0.092
**C25**	3.404	3.405	0.001	3.215	−0.190
**C26** *^d^*	3.564	3.408	−0.156	3.423	−0.141
**C27**	3.070	3.118	0.048	2.984	−0.086
**C28**	3.016	2.981	−0.035	3.165	0.149
**C29**	3.228	3.271	0.043	3.203	−0.025
**C30**	3.073	3.137	0.064	3.040	−0.033
**C31**	3.679	3.710	0.030	3.651	−0.028
**C32**	3.765	3.805	0.039	3.769	0.004
**C33**	3.587	3.580	−0.007	3.625	0.038
**C34**	3.758	3.639	−0.119	3.745	−0.013
**C35** *^e^*	3.743	3.675	−0.067	3.685	−0.058

*^a^* Experimental pEC_50_. *^b^* Predicted pEC_50_. *^c^* Residual error (experimental prediction). *^d^* Testing samples. *^e^* Compound synthesized basis on the CoMFA and CoMSIA models.

**Table 4:** Calculated Binding Free Energies (kcal/mol) of **C35** and NNM with PVY Protein.

Compd.	ΔE_vdw_	ΔE_ele_	ΔE_MM_	ΔG_sol_	ΔE_bind_	−TΔS	ΔG_bind_
C35 ^a^	−184.4	−57.7	−242.1	142.1	−100.0	72.9	−27.1
NNM ^a^	−145.5	−43.8	−189.3	118.4	−70.9	38.5	−32.4

^a^: Free energies and their components were obtained from MM-PBSA calculations.

## Data Availability

All data generated in this study are presented in the current manuscript. No new datasets were generated. Data are available upon request from the corresponding authors.

## Supplementary Materials

The following supporting information can be downloaded at: https://www.mdpi.com/article/10.3390/ijms24098280/s1. All physical data, high-resolution spectra, and NMR data of compound **C1**–**C35** are presented in Figures S1–S113 in Supporting Information.
