# Supplementary material for: Design, Synthesis, and Anti-PVY Biological Activity of 1,3,5-Triazine Derivatives Containing Piperazine Structure
Source: Int J Mol Sci. 2023 May 5;24(9):8280. doi: 10.3390/ijms24098280 (PMC10179359; doi:10.3390/ijms24098280)
Supplement: Supplementary file 1 [file ijms-24-08280-s001.zip › ijms-2368779-supplementary.pdf]

Supplementary Information

Design, Synthesis, And Anti-PVY Biological Activity of 1,3,5-Triazine Derivatives Containing Piperazine Structure

Lian Bai, Chunle Wei, Jian Zhang *, Runjiang Song*

Center for R&D of Fine Chemicals of Guizhou University, Key Laboratory of Green Pesticide and Agricultural Bi-oengineering, Ministry of Education, National Key Laboratory of Green Pesticide, Guiyang 550025, China; 15885174719@163.com (L.B.); weichle@163.com (C.W.)

* Correspondence: jianzhang@gzu.edu.cn (J.Z.); songrj@gzu.edu.cn (R.S.)

Table of Contents

1. Characterizations of products………………………………………………… 3−23

2. ^{1}H, ^{13}C, ^{19}F NMR and HRMS data…………………………………………24−80

Characterization of target products (C1-C35)

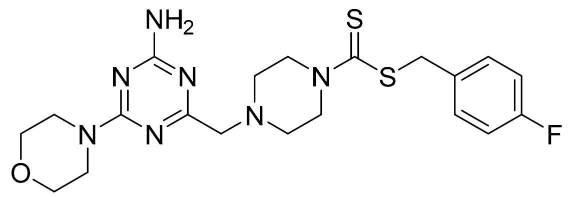

4-fluorobenzyl-4-((4-amino-6-morpholino-1,3,5-triazin-2-yl)methyl)piperazine-1-carbodithioate (C1): Yield: 77.29%; white solid; m. p. 78.1-78.9 °C; ^{1}H NMR (500 MHz, DMSO-d_6) δ 7.39 (dd, *J* = 8.7, 5.5 Hz, 2H, Ar-H), 7.10 (t, *J* = 8.9 Hz, 2H, Ar-H), 6.84 (d, *J* = 58.4 Hz, 2H, -NH_2), 4.49 (s, 2H, -S-CH_2), 4. 02 (d, *J* = 178.4 Hz, 4H, Piperazine-H), 3.61 (d, *J* = 5.0 Hz, 4H, Morpholine -H), 3.56 – 3.52 (m, 4H, -CH_2-O-CH_2-), 3.27 (s, 2H, -CH_2-N-), 2.58 (s, 4H, P iperazine-H). ^{13}C NMR (126 MHz, DMSO-d_6) δ 194.85, 174.19, 167.30, 165.1 0, 162.92, 160.98, 133.20, 131.74, 131.68, 115.86, 115.69, 66.46 (2), 63.41 (2), 52.51 (2), 51.68, 50.30, 40.28. HRMS (ESI): Calculated for $C_{20}H_{27}FN_7OS_2$ [M +H]$^+$: 464.16970, found: 464.16843.

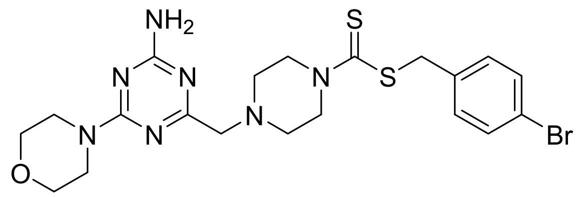

4-bromobenzyl-4-((4-amino-6-morpholino-1,3,5-triazin-2-yl)methyl)piperazine-1 -carbodithioate (C2): Yield: 84.16%; white solid; m. p. 113.2-114.0 °C; ^{1}H N MR (500 MHz, DMSO-d_6) δ 7.46 (d, *J* = 8.1 Hz, 2H, Ar-H), 7.30 (d, *J* = 8. 3 Hz, 2H, Ar-H), 6.84 (d, *J* = 56.4 Hz, 2H, -NH_2), 4.49 (s, 2H, -S-CH_2), 4.0

2 (d, J = 174.2 Hz, 4H, Piperazine-H), 3.67 – 3.59 (m, 4H, Morpholine-H), 3.54 (t, J = 4.8 Hz, 4H, $-CH_2-O-CH_2-$), 3.27 (s, 2H, $-CH_2-N-$), 2.59 (s, 4H, Piperazine-H). ^{13}C NMR (126 MHz, DMSO-d_6) δ 194.69, 174.18, 167.30, 165.10, 136.75, 131.88 (2), 131.84 (2), 120.96, 66.46 (2), 63.39 (2), 52.50 (2), 51.80, 50.33, 43.61, 40.64. HRMS (ESI): Calculated for $C_{20}H_{27}BrN_7OS_2$ $[M+H]^+$: 524.08964, found: 524.08820.

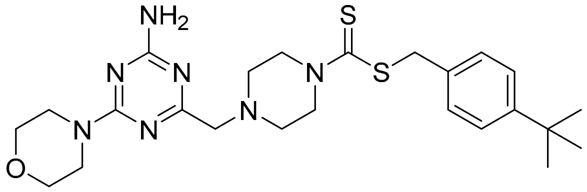

4-(tert-butyl)benzyl-4-((4-amino-6-morpholino-1,3,5-triazin-2-yl)methyl)piperazine-1-carbodithioate (C3): Yield: 84.03%; white solid; m. p. 171.3-171.6 °C; ^1H NMR (500 MHz, DMSO-d_6) δ 7.27 (q, J = 8.0 Hz, 4H, Ar-H), 6.84 (d, J = 57.7 Hz, 2H, $-NH_2$), 4.43 (s, 2H, $-S-CH_2$), 4.01 (d, J = 181.5 Hz, 4H, 4H, Piperazine-H), 3.61 (s, 4H, Morpholine-H), 3.57 – 3.50 (m, 4H, 4H, $-CH_2-O-CH_2-$), 3.27 (s, 2H, $-CH_2-N-$), 2.58 (s, 4H, Piperazine-H), 1.21 (s, 9H, $-C(CH_3)_3$). ^{13}C NMR (126 MHz, DMSO-d_6) δ 195.14, 174.20, 167.30, 165.10, 150.38, 133.57, 129.49 (2), 125.79 (2), 66.46 (2), 63.41 (2), 52.50 (2), 51.59, 50.24, 43.62, 41.03, 34.76, 31.62 (3). HRMS (ESI): Calculated for $C_{24}H_{36}N_7OS_2$ $[M+H]^+$: 502.24173, found: 502.24008.

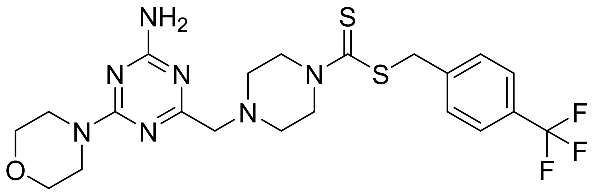

4-(trifluoromethyl)benzyl-4-((4-amino-6-morpholino-1,3,5-triazin-2-yl)methyl)piperazine-1-carbodithioate (C4): Yield: 71.84%; white solid; m. p. 114.8-115.4 °C; ^{1}H NMR (500 MHz, DMSO-d_6) δ 7.64 (d, J = 8.1 Hz, 2H, Ar-H), 7.57 (d, J = 8.5 Hz, 2H, Ar-H), 6.84 (d, J = 55.1 Hz, 2H, -NH_2), 4.62 (s, 2H, -S-CH_2), 4.03 (d, J = 168.4 Hz, 4H, Piperazine-H), 3.66 – 3.59 (m, 4H, Morpholine-H), 3.56 – 3.51 (m, 4H, -CH_2-O-CH_2-), 3.27 (s, 2H, -CH_2-N-), 2.60 (s, 4H, Piperazine-H).^{13}C NMR (126 MHz, DMSO-d_6) δ 194.48, 174.19, 167.30, 165.10, 142.48, 130.44, 128.21, 125.79, 125.74, 123.69, 66.45 (2), 63.38 (2), 52.50 (2), 51.95, 50.39, 43.62, 40.14. HRMS (ESI): Calculated for $C_{21}H_{27}F_3N_7OS_2$ $[M+H]^+$: 514.16651, found: 514.16497.

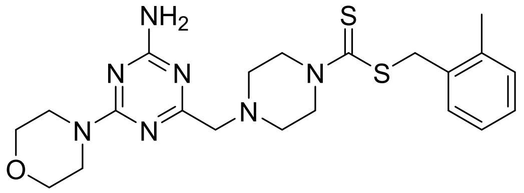

2-methylbenzyl-4-((4-amino-6-morpholino-1,3,5-triazin-2-yl)methyl)piperazine-1-carbodithioate (C5): Yield: 94.73%; white solid; m. p. 138.4-138.7 °C; ^{1}H NMR (400 MHz, DMSO-d_6) δ 7.36 (d, J = 7.1 Hz, 1H, Ar-H), 7.24 – 7.18 (m, 2H, Ar-H), 7.15 (dd, J = 7.5, 4.2 Hz, 1H, Ar-H), 6.88 (d, J = 39.4 Hz, 2H, -NH_2), 4.46 (s, 2H, 2H, -S-CH_2), 4.06 (d, J = 147.7 Hz, 4H, Piperazine-H), 3.66 (d, J = 4.2 Hz, 4H, Morpholine-H), 3.61 – 3.56 (m, 4H, -CH_2-O-CH_2-), 3.33 (s, 2H, -CH_2-N-)2.64 (s, 4H, Piperazine-H), 2.32 (s, 3H, -Ar-CH_3). ^{13}C NMR (101 MHz, DMSO-d_6) δ 194.95, 174.08, 167.22, 165.03, 137.33, 133.88, 130.76, 130.67, 128.30, 126.59, 66.40 (2), 63.31 (2), 52.44 (2), 51.33, 50.18, 43.

55, 40.12, 19.34. HRMS (ESI): Calculated for $C_{21}H_{30}N_7OS_2$ $[M+H]^+$: 460.1947 8, found: 460.19312.

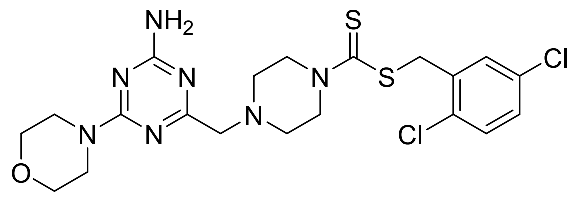

2,5-dichlorobenzyl-4-((4-amino-6-morpholino-1,3,5-triazin-2-yl)methyl)piperazine-1-carbodithioate (C6): Yield: 69.37%; white solid; m. p. 138.4-138.7 °C; ^1H NMR (500 MHz, DMSO-d_6) δ 7.63 (d, J = 2.6 Hz, 1H, Ar-H), 7.47 (d, J = 8.6 Hz, 1H, Ar-H), 7.37 (dd, J = 8.6, 2.7 Hz, 1H, Ar-H), 6.86 (d, J = 64.3 Hz, 2H, -NH_2), 4.56 (s, 2H, -S-CH_2), 4.02 (d, J = 172.5 Hz, 4H, Piperazine-H), 3.61 (s, 4H, Morpholine-H), 3.54 (d, J = 4.8 Hz, 4H, -CH_2-O-CH_2-), 3.27 (s, 2H, -CH_2-N-), 2.59 (s, 4H, Piperazine-H).^{13}C NMR (126 MHz, DMSO-d_6) δ 193.96, 174.16, 167.29, 165.08, 136.71, 132.76, 132.18, 131.61, 131.51, 129. 80, 66.45 (2), 63.39 (2), 52.49 (2), 51.82, 50.45, 43.58, 38.67.HRMS (ESI): C alculated for $C_{20}H_{26}Cl_2N_7OS_2$ $[M+H]^+$: 514.10118, found: 514.09973.

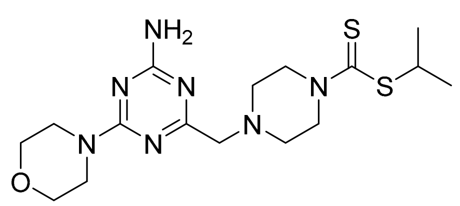

Isobutyl-4-((4-amino-6-morpholino-1,3,5-triazin-2-yl)methyl)piperazine-1-carbodithioate (C7): Yield: 76.43%; white solid; m. p. 139.6-139..9 °C; ^1H NMR (500 MHz, DMSO-d_6) δ 6.88 (d, J = 63.6 Hz, 2H, -NH_2), 4.21 (s, 2H, Pipera

zine-H), 3.95 – 3.89 (m, 1H, -<u>CH</u>(CH_3)$_2$), 3.89 – 3.81 (m, 2H, Piperazine-H), 3.66 (s, 4H, Morpholine-H), 3.60 – 3.57 (m, 4H, -CH_2-O-CH_2-), 3.34 (s, 2H, -CH_2-N-), 2.61 (t, J = 5.1 Hz, 4H, Piperazine-H), 1.35 (d, J = 6.9 Hz, 6H, -CH<u>(CH_3)</u>$_2$). ^{13}C NMR (126 MHz, DMSO-d_6) δ 194.97, 174.21, 167.30, 165.10, 66.46 (2), 63.41 (2), 52.49 (2), 50.96, 50.20, 43.61, 42.43, 23.01 (2).HRMS (ESI): Calculated for $C_{17}H_{30}N_7OS_2$ [M+H]$^+$: 412.19478, found: 412.19423.

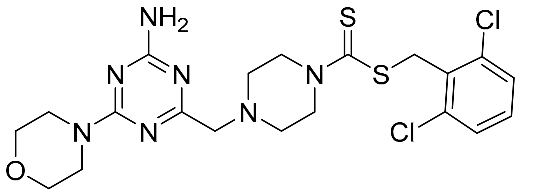

2,6-dichlorobenzyl-4-((4-amino-6-morpholino-1,3,5-triazin-2-yl)methyl)piperazine-1-carbodithioate (C8): Yield:87.98%; white solid; m. p. 123.0-123.3 °C; ^1H NMR (400 MHz, DMSO-d_6) δ 7.52 (d, J = 7.7 Hz, 2H, Ar-H), 7.39 (dd, J = 8.7, 7.4 Hz, 1H, Ar-H), 6.88 (d, J = 39.5 Hz, 2H, -NH_2), 4.61 (s, 2H, -S-CH_2), 4.05 (d, J = 157.8 Hz, 4H, Piperazine-H), 3.67 (s, 4H, Morpholine-H), 3.61 – 3.57 (m, 4H, -CH_2-O-CH_2-), 3.33 (s, 2H, -CH_2-N-), 2.64 (d, J = 12.9 Hz, 4H, Piperazine-H). ^{13}C NMR (101 MHz, DMSO-d_6) δ 193.92, 174.13, 167.23, 165.03, 135.87 (2), 131.18, 130.93, 129.25 (2), 66.40 (2), 63.29 (2), 52.36 (2), 51.36, 50.35, 43.56, 38.43.HRMS (ESI): Calculated for $C_{20}H_{26}Cl_2N_7OS_2$ [M+H]$^+$: 514.10118, found: 514.09967.

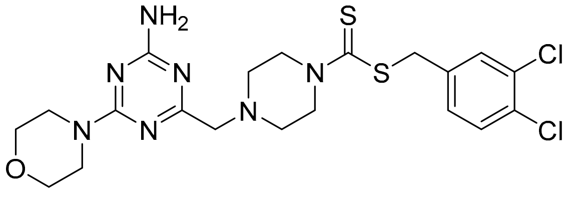

3,4-dichlorobenzyl-4-((4-amino-6-morpholino-1,3,5-triazin-2-yl)methyl)piperazine-1-carbodithioate (C9): Yield: 66.19%; white solid; m. p. 94.4-95.1 °C; ^1H NMR (500 MHz, DMSO-d_6) δ 7.66 (d, J = 2.2 Hz, 1H, Ar-H), 7.57 (d, J = 8.6 Hz, 1H, Ar-H), 7.38 (dd, J = 8.1, 2.2 Hz, 1H, Ar-H), 7.04 – 6.72 (m, 2H, -NH_2), 4.57 (s, 2H, -S-CH_2), 4.06 (d, J = 170.2 Hz, 4H, Piperazine-H), 3.65 (s, 4H, Morpholine-H), 3.58 (d, J = 4.7 Hz, 4H, -CH_2-O-CH_2-), 3.31 (s, 2H, -CH_2-N-), 2.63 (s, 4H, Piperazine-H). ^{13}C NMR (126 MHz, DMSO-d_6) δ 194.36, 174.17, 167.29, 165.09, 138.88, 131.52, 131.35, 131.05, 130.38, 130.02, 66.46 (2), 63.38 (2), 52.50 (2), 51.93, 50.40, 43.60, 39.38. HRMS (ESI): Calculated for $C_{20}H_{26}Cl_2N_7OS_2$ $[M+H]^+$: 514.10118, found: 514.09967.

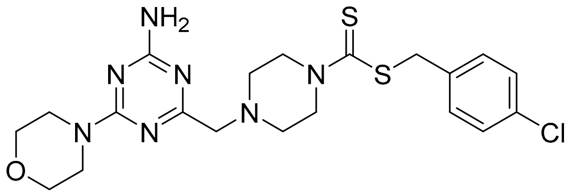

4-chlorobenzyl-4-((4-amino-6-morpholino-1,3,5-triazin-2-yl)methyl)piperazine-1-carbodithioate (C10): Yield: 89.44%; white solid; m. p. 75.3-75.6 °C; ^1H NMR (500 MHz, DMSO-d_6) δ 7.41 (d, J = 8.6 Hz, 2H, Ar-H), 7.36 (d, J = 8.5 Hz, 2H, Ar-H), 6.88 (d, J = 58.4 Hz, 2H, -NH_2), 4.54 (s, 2H, -S-CH_2), 4.06 (d, J = 175.1 Hz, 4H, Piperazine-H), 3.65 (t, J = 4.7 Hz, 4H, Morpholine-H), 3.58 (t, J = 4.7 Hz, 4H, -CH_2-O-CH_2-), 3.31 (s, 2H, -CH_2-N-), 2.63 (s, 4H, Piperazine-H). ^{13}C NMR (126 MHz, DMSO-d_6) δ 194.70, 174.17, 167.29, 165.09, 136.29, 132.46, 131.54 (2), 128.92 (2), 66.46 (2), 63.39 (2), 52.50 (2), 51.78, 50.32, 43.60, 40.62. HRMS (ESI): Calculated for $C_{20}H_{27}ClN_7OS_2$ $[M+H]^+$: 480.14015, found: 480.13867.

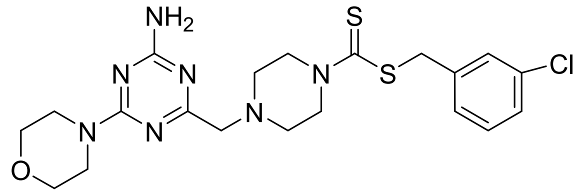

3-chlorobenzyl-4-((4-amino-6-morpholino-1,3,5-triazin-2-yl)methyl)piperazine-1-carbodithioate (C11): Yield: 98.15%; white solid; m. p. 113.4-114.0 °C; ^{1}H NMR (500 MHz, DMSO-d_6) δ 7.42 (s, 1H, Ar-H), 7.32 – 7.27 (m, 3H, Ar-H), 6.85 (d, J = 60.1 Hz, 2H, -NH$_2$), 4.52 (s, 2H, -S-CH$_2$), 4.03 (d, J = 172.3 Hz, 4H, Piperazine-H), 3.62 (s, 4H, Morpholine-H), 3.55 (d, J = 4.8 Hz, 4H, -CH$_2$-O-CH$_2$-), 3.28 (s, 2H, 2H, -CH$_2$-N-), 2.59 (s, 4H, Piperazine-H). ^{13}C NMR (101 MHz, DMSO-d_6) δ 195.72, 174.34, 166.93, 164.86, 138.36, 134.30, 129.80, 129.39, 127.70, 127.55, 66.72 (2), 63.60 (2), 52.73 (2), 51.24, 49.73, 43.49, 41.23. HRMS (ESI): Calculated for $C_{20}H_{27}ClN_7OS_2$ [M+H]$^+$: 480.14015, found: 480.13855.

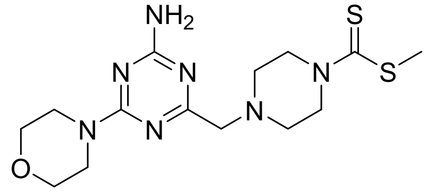

Methyl-4-((4-amino-6-morpholino-1,3,5-triazin-2-yl)methyl)piperazine-1-carbodithioate (C12): Yield: 83.12%; white solid; m. p. 174.6-174.9 °C; ^{1}H NMR (500 MHz, DMSO-d_6) δ 6.84 (d, J = 60.6 Hz, 2H, -NH$_2$), 4.02 (d, J = 167.6 Hz, 4H, Piperazine-H), 3.62 (s, 4H, Morpholine-H), 3.57 – 3.53 (m, 4H, -CH$_2$-O-CH$_2$-), 3.27 (s, 2H, -CH$_2$-N-), 2.59 (t, J = 5.1 Hz, 4H, Piperazine-H), 2.52 (s, 3H, -S-CH$_3$). ^{13}C NMR (126 MHz, DMSO-d_6) δ 196.69, 174.19, 167.29, 165.

10, 66.46 (2), 63.45 (2), 52.50 (2), 51.61, 50.14, 43.60, 20.00. HRMS (ESI): Calculated for $C_{14}H_{24}N_7OS_2$ [M+H]$^+$: 370.14783, found: 370.14648.

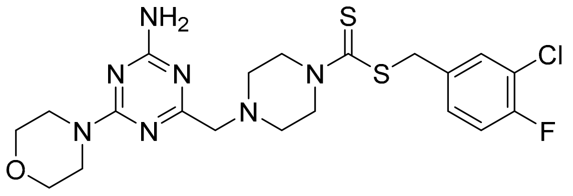

3-chloro-4-fluorobenzyl-4-((4-amino-6-morpholino-1,3,5-triazin-2-yl)methyl)piperazine-1-carbodithioate (C13): Yield: 71.11%; white solid; m. p. 115.4-115.8 °C; ^{1}H NMR (500 MHz, DMSO-d_6) δ 7.63 (dd, J = 7.3, 2.2 Hz, 1H, Ar-H), 7.42 (ddd, J = 8.6, 4.8, 2.3 Hz, 1H, Ar-H), 7.36 (t, J = 8.9 Hz, 1H, Ar-H), 6.91 (d, J = 62.6 Hz, 2H, -NH$_2$), 4.56 (s, 2H, -S-CH$_2$), 4.07 (d, J = 173.8 Hz, 4H, Piperazine-H), 3.66 (s, 4H, Morpholine-H), 3.59 (d, J = 4.4 Hz, 4H, -CH$_2$-O-CH$_2$-), 3.32 (s, 2H, -CH$_2$-N-), 2.63 (s, 4H, Piperazine-H). ^{13}C NMR (126 MHz, DMSO-d_6) δ 194.45, 174.17, 167.29, 165.08, 157.93, 155.97, 135.43, 135.40, 131.60, 130.44, 130.38, 119.76, 119.62, 117.45, 117.29, 66.45 (2), 63.40 (2), 52.50 (2), 51.83, 50.36, 43.58, 39.44. HRMS (ESI): Calculated for $C_{20}H_{26}ClFN_7OS_2$ [M+H]$^+$: 498.13073, found: 498.12903.

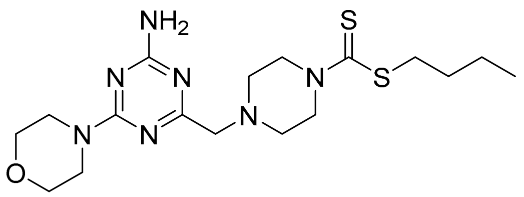

Butyl-4-((4-amino-6-morpholino-1,3,5-triazin-2-yl)methyl)piperazine-1-carbodithioate (C14): Yield: 73.05%; white solid; m. p. 117.6-118.3 °C; ^{1}H NMR (400 MHz, DMSO-d_6) δ 6.89 (d, J = 43.8 Hz, 2H, -NH$_2$), 4.23 (s, 4H, Piperazine-H), 3.66 (d, J = 3.8 Hz, 4H, Morpholine-H), 3.62 – 3.55 (m, 4H, -CH$_2$-O-C

H2-), 3.32 (s, 2H, -CH2-N-), 3.23 (t, *J* = 7.4 Hz, 2H, -S-CH2-), 2.63 (t, *J* = 5.1 Hz, 4H, Piperazine-H), 1.67 – 1.53 (m, 2H, -<u>CH2</u>-CH2-), 1.37 (h, *J* = 7.3 Hz, 2H, -<u>CH2</u>-CH3), 0.89 (t, *J* = 7.3 Hz, 3H, -CH2-<u>CH3</u>). ^{13}C NMR (101 MHz, DMSO-*d*6) δ 195.68, 174.12, 167.22, 165.02, 66.40 (2), 63.35 (2), 52.43 (2), 51.38, 50.13, 43.54, 36.37, 30.90, 21.99, 14.02. HRMS (ESI): Calculated for $C_{17}H_{30}N_7OS_2$ [M+H]$^+$: 412.19478, found: 412.09315.

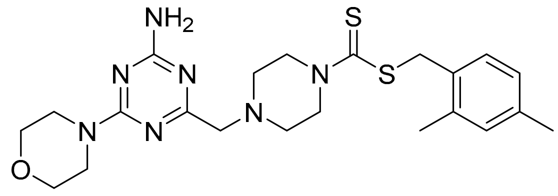

2,4-dimethylbenzyl-4-((4-amino-6-morpholino-1,3,5-triazin-2-yl)methyl)piperazine-1-carbodithioate (C15): Yield: 68.19%; white solid; m. p. 110.0-110.7 °C; ^{1}H NMR (500 MHz, DMSO-*d*6) δ 7.13 (d, *J* = 1.8 Hz, 1H, Ar-H), 7.09 – 7.04 (m, 2H, Ar-H), 7.01 – 6.80 (m, 2H, -NH2), 4.41 (s, 2H, -S-CH2), 4.05 (d, *J* = 185.6 Hz, 4H, Piperazine-H), 3.65 (s, 4H, Morpholine-H), 3.58 (d, *J* = 4.4 Hz, 4H, -CH2-O-CH2-), 3.36 (s, 2H, -CH2-N-), 2.60 (s, 4H, Piperazine-H), 2.17 (s, 6H, Ar-CH3). ^{13}C NMR (126 MHz, DMSO-*d*6) δ 195.16, 174.19, 167.29, 165.08, 136.84, 135.95, 133.61, 130.86, 130.09, 127.19, 66.46 (2), 63.43 (2), 52.51 (2), 51.48, 50.20, 43.58, 41.37, 19.87, 19.59. HRMS (ESI): Calculated for $C_{22}H_{32}N_7OS_2$ [M+H]$^+$: 474.21043, found: 474.20868.

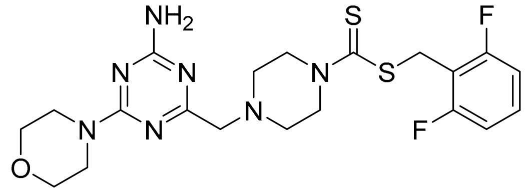

2,6-difluorobenzyl-4-((4-amino-6-morpholino-1,3,5-triazin-2-yl)methyl)piperazine-1-carbodithioate (C16): Yield: 62.17%; white solid; m. p. 152.0-152.3 °C; ^{1}H NMR (500 MHz, DMSO-d_6) δ 7.40 (tt, J = 8.5, 6.6 Hz, 1H, Ar-H), 7.10 (t, J = 8.0 Hz, 2H, Ar-H), 6.84 (d, J = 61.3 Hz, 2H, -NH_2), 4.43 (s, 2H, -S-CH_2), 4.19 (s, 4H, Piperazine-H), 3.61 (d, J = 4.5 Hz, 4H, Morpholine-H), 3.55 (d, J = 5.0 Hz, 4H, -CH_2-O-CH_2-), 3.28 (s, 2H, -CH_2-N-), 2.65 – 2.55 (m, 4H, Piperazine-H). ^{13}C NMR (126 MHz, DMSO-d_6) δ 193.85, 174.20, 167.30, 165.09, 162.41, 162.35, 160.43, 160.37, 131.20, 131.12, 131.04, 112.43, 112.40, 112.27, 112.24, 111.70, 111.55, 111.39, 66.45 (2), 63.33 (2), 52.42 (2), 51.53, 50.34, 43.61, 29.16. HRMS (ESI): Calculated for $C_{20}H_{26}F_2N_7OS_2$ $[M+H]^+$: 482.16028, found: 482.15860.

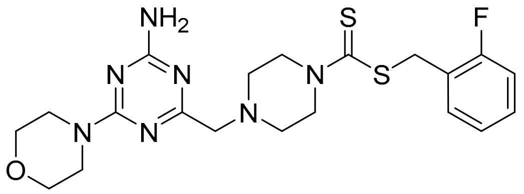

2-fluorobenzyl-4-((4-amino-6-morpholino-1,3,5-triazin-2-yl)methyl)piperazine-1-carbodithioate (C17): Yield: 77.18%; white solid; m. p. 115.4-115.9 °C; ^{1}H NMR (500 MHz, DMSO-d_6) δ 7.47 (td, J = 7.8, 1.9 Hz, 1H, Ar-H), 7.30 (tdd, J = 7.6, 5.4, 1.8 Hz, 1H, Ar-H), 7.18 – 7.10 (m, 2H, Ar-H), 6.86 (d, J = 66.7 Hz, 2H, -NH_2), 4.48 (s, 2H, -S-CH_2), 4.19 (s, 4H, Piperazine-H), 3.61 (s, 4H, Morpholine-H), 3.54 (d, J = 4.8 Hz, 4H, -CH_2-O-CH_2-), 3.27 (s, 2H, -CH_2-N-), 2.58 (s, 4H, Piperazine-H). ^{13}C NMR (126 MHz, DMSO-d_6) δ 194.39, 174.19, 167.29, 165.08, 161.99, 160.03, 132.11, 132.08, 130.38, 130.31, 125.08, 125.05, 123.62, 123.50, 116.01, 115.84, 66.45 (2), 63.40 (2), 52.49 (2), 51.64, 5

0.31, 43.59, 34.74. HRMS (ESI): Calculated for $C_{20}H_{27}FN_7OS_2$ $[M+H]^+$: 464.16970, found: 464.16809.

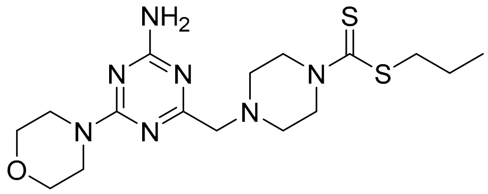

Propyl-4-((4-amino-6-morpholino-1,3,5-triazin-2-yl)methyl)piperazine-1-carbodithioate (C18) : Yield: 52.79%; white solid; m. p. 106.2-106.4 °C; ^{1}H NMR (400 MHz, DMSO-d_6) δ 6.89 (d, J = 39.1 Hz, 2H, -NH_2), 4.07 (d, J = 129.5 Hz, 4H, Piperazine-H), 3.66 (s, 4H, Morpholine-H), 3.60 (d, J = 4.8 Hz, 4H, -CH_2-O-CH_2-), 3.32 (s, 2H, -CH_2-N-), 3.21 (t, J = 6.4 Hz, 2H, -S-CH_2-), 2.63 (s, 4H, Piperazine-H), 1.64 (q, J = 7.3 Hz, 2H, -$\underline{CH_2}$-CH_3), 0.95 (t, J = 7.4 Hz, 3H, -CH_2-$\underline{CH_3}$). ^{13}C NMR (101 MHz, DMSO-d_6) δ 195.68, 174.13, 167.22, 165.02, 66.40 (2), 63.35 (2), 52.44 (2), 51.41, 50.15, 43.55, 38.52, 22.29, 13.80. HRMS (ESI): Calculated for $C_{16}H_{28}N_7OS_2$ $[M+H]^+$: 398.17913, found: 398.17749.

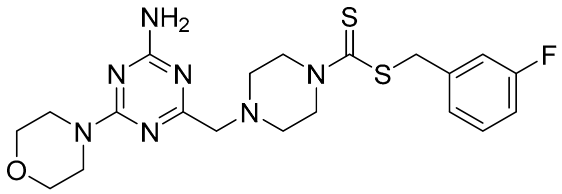

3-fluorobenzyl 4-((4-amino-6-morpholino-1,3,5-triazin-2-yl)methyl)piperazine-1-carbodithioate (C19) : Yield: 73.24%; white solid; m. p. 121.4-121.7 °C; ^{1}H NMR (500 MHz, DMSO-d_6) δ 7.35 (q, J = 7.5 Hz, 1H, Ar-H), 7.23 (d, J = 7.4 Hz, 2H, Ar-H), 7.12 – 7.07 (m, 1H, Ar-H), 6.90 (d, J = 64.9 Hz, 2H, -NH_2), 4.56 (s, 2H, -S-CH_2), 4.07 (d, J = 174.7 Hz, 4H, Piperazine-H), 3.65 (s,

4H, Morpholine-H), 3.58 (d, J = 4.5 Hz, 4H, -CH_2-O-CH_2-), 3.31 (s, 2H, -CH $_2$-N-), 2.63 (s, 4H, Piperazine-H).^{13}C NMR (126 MHz, $CDCl_3$) δ 195.79, 174.3 3, 166.91, 164.84, 138.79, 138.72, 130.06, 129.97, 125.01, 124.98, 116.34, 116. 12, 114.58, 114.37, 66.71 (2), 63.58 (2), 52.72 (2), 51.21, 49.71, 41.34, 41.32. HRMS (ESI): Calculated for $C_{20}H_{27}FN_7OS_2$ $[M+H]^+$: 464.16970, found: 464.168 12.

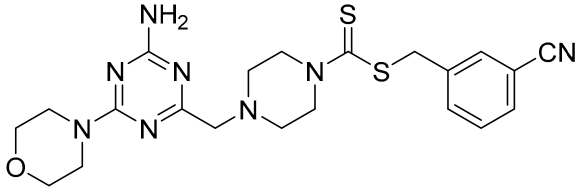

3-cyanobenzyl-4-((4-amino-6-morpholino-1,3,5-triazin-2-yl)methyl)piperazine-1-carbodithioate (C20): Yield: 68.12%; white solid; m. p. 112.4-112.8 °C; ^{1}H N MR (500 MHz, DMSO-d_6) δ 7.85 (s, 1H, Ar-H), 7.73 (d, J = 10.8 Hz, 2H, Ar-H), 7.53 (t, J = 7.8 Hz, 1H, Ar-H), 6.90 (d, J = 63.7 Hz, 2H, -NH_2), 4.61 (s, 2H, -S-CH_2), 4.06 (d, J = 169.6 Hz, 4H, Piperazine-H), 3.65 (s, 4H, Mor pholine-H), 3.58 (s, 4H, -CH_2-O-CH_2-), 3.31 (s, 2H, -CH_2-N-), 2.62 (s, 4H, Pi perazine-H). ^{13}C NMR (126 MHz, $CDCl_3$) δ 195.04, 174.30, 166.92, 164.85, 1 38.55, 133.81, 132.77, 131.04, 129.26, 118.69, 112.56, 66.71 (2), 63.57 (2), 52. 71 (2), 51.48, 49.80, 43.48, 40.44. HRMS (ESI): Calculated for $C_{21}H_{27}N_8OS_2$ $[M+H]^+$: 471.17438, found: 471.17239.

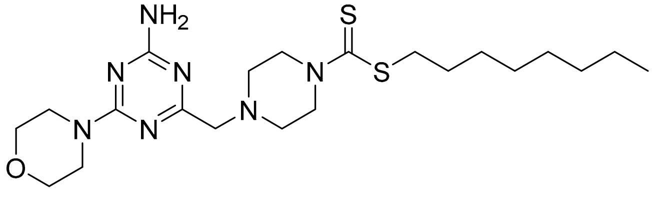

Octyl-4-((4-amino-6-morpholino-1,3,5-triazin-2-yl)methyl)piperazine-1-carbodithioate (C21): Yield:62.87%; white solid; m. p. 68.1-68.4 °C; ^{1}H NMR (400 MHz, $CDCl_3$) δ 5.33 – 4.98 (m, 2H, $-NH_2$), 4.22 (d, *J* = 164.6 Hz, 4H, Piperazine-H), 3.79 (s, 4H, Morpholine-H), 3.71 (dd, *J* = 5.6, 3.8 Hz, 4H, $-CH_2-O-CH_2-$), 3.44 (s, 2H, $-S-CH_2$), 3.32 – 3.27 (m, 2H, $-CH_2-N-$), 2.69 (t, *J* = 5.1 Hz, 4H, Piperazine-H), 1.69 (s, 2H, $-S-CH_2-\underline{CH_2}-$), 1.42 (td, *J* = 8.7, 5.0 Hz, 2H, $-\underline{CH2}-(CH_2)_4-CH_3$), 1.32 – 1.25 (m, 8H, $-(\underline{CH_2})_4-CH_3$), 0.90 – 0.85 (m, 3H, $-CH_2-\underline{CH_3}$). ^{13}C NMR (101 MHz, $CDCl_3$) δ 197.35, 174.37, 166.87, 164.83, 77.39, 77.07, 76.76, 66.72 (2), 63.60 (2), 52.76 (2), 37.33, 31.82, 29.73, 29.19 (2), 29.07, 28.63, 22.67, 14.15. HRMS (ESI): Calculated for $C_{21}H_{38}N_7OS_2$ $[M+H]^+$: 468.25738, found: 468.25558.

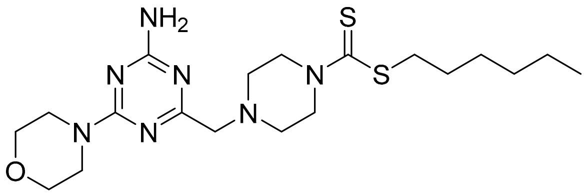

Hexyl-4-((4-amino-6-morpholino-1,3,5-triazin-2-yl)methyl)piperazine-1-carbodithioate (C22): Yield:55.26%; white solid; m. p. 68.1-68.4 °C; ^{1}H NMR (500 MHz, $CDCl_3$) δ 5.21 (s, 2H, $-NH_2$), 4.19 (d, *J* = 206.8 Hz, 4H, Piperazine-H), 3.76 (s, 4H, Morpholine-H), 3.69 – 3.66 (m, 4H, $-CH_2-O-CH_2-$), 3.40 (s, 2H, $-S-CH_2$), 3.28 – 3.24 (m, 2H, $-CH_2-N-$), 2.66 (s, 4H, Piperazine-H), 1.66 (p, *J* = 7.5 Hz, 2H, $-S-CH_2-\underline{CH_2}-$), 1.40 (d, *J* = 6.9 Hz, 2H, $-\underline{CH2}-(CH_2)_2-CH_3$), 1.30 – 1.24 (m, 4H, $-(\underline{CH_2})_2-CH_3$), 0.88 – 0.84 (m, 3H, $-CH_2-\underline{CH_3}$). ^{13}C NMR (126 MHz, $CDCl_3$) δ 197.42, 174.44, 166.97, 164.91, 66.79 (2), 63.67 (2), 52.83

(2), 50.90, 49.62, 43.54, 37.39, 31.46, 28.80, 28.66, 22.62, 14.14. HRMS (ESI): Calculated for $C_{21}H_{34}N_7OS_2$ [M+H]$^+$: 440.22608, found: 440.22421.

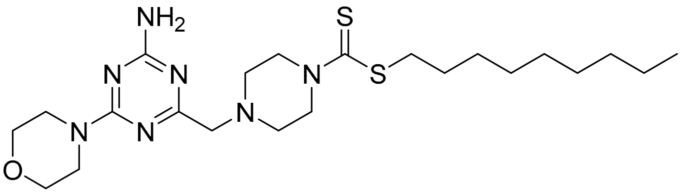

Dodecyl-4-((4-amino-6-morpholino-1,3,5-triazin-2-yl)methyl)piperazine-1-carbodithioate (C23): Yield: 48.11%; white solid; m. p. 67.2-67.5 °C; ^{1}H NMR (500 MHz, $CDCl_3$) δ 5.26 (s, 2H, $-NH_2$), 4.43 (s, 4H, Piperazine-H), 3.79 (s, 4H, Morpholine-H), 3.71 (t, J = 4.5 Hz, 4H, $-CH_2-O-CH_2-$), 3.44 (s, 2H, $-S-CH_2$), 3.29 (t, J = 7.5 Hz, 2H, $-CH_2-N-$), 2.70 (s, 4H, Piperazine-H), 1.70 (p, J = 7.6 Hz, 2H, $-S-CH_2-\underline{CH_2}-$), 1.40 (q, J = 7.4 Hz, 2H, $-\underline{CH2}-(CH_2)_5-CH_3$), 1.28 (dd, J = 12.7, 5.6 Hz, 10H, $-(\underline{CH_2})_5-CH_3$), 0.90 – 0.86 (m, 3H,- $CH_2-\underline{CH_3}$). ^{13}C NMR (126 MHz, $CDCl_3$) δ 197.42, 174.45, 166.99, 164.92, 66.79 (2), 63.68 (2), 50.91 (2), 49.62, 43.54, 37.39, 31.95, 29.54, 29.34, 29.29, 29.13, 28.70, 22.76, 14.22. HRMS (ESI): Calculated for $C_{22}H_{40}N_7OS_2$ [M+H]$^+$: 482.27303, found: 482.27127.

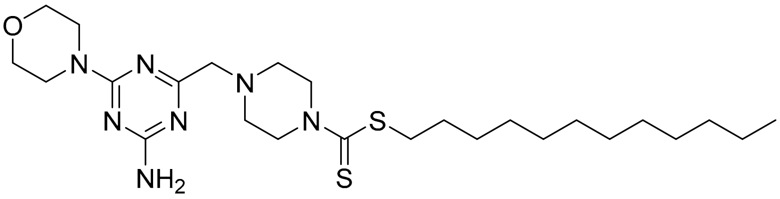

Dodecyl-4-((4-amino-6-morpholino-1,3,5-triazin-2-yl)methyl)piperazine-1-carbodithioate (C24): Yield: 44.94%; white solid; m. p. 70.8-71.3 °C; ^{1}H NMR (500 MHz, $CDCl_3$) δ 5.23 (s, 2H, $-NH_2$), 4.19 (d, J = 207.5 Hz, 4H, Piperazine-

H), 3.75 (s, 4H, Morpholine-H), 3.67 (d, J = 4.3 Hz, 4H, $-CH_2-O-CH_2-$), 3.40 (s, 2H, $-S-CH_2$), 3.25 (t, J = 7.5 Hz, 2H, $-CH_2-N-$), 2.66 (s, 4H, Piperazine-H), 1.66 (t, J = 7.5 Hz, 2H, $-S-CH_2-\underline{CH_2}-$), 1.37 (t, J = 7.7 Hz, 2H, $-\underline{CH2}-(CH_2)_8-CH_3$), 1.22 (s, 16H, $-(\underline{CH_2})_8-CH_3$), 0.84 (t, J = 6.8 Hz, 3H, $-CH_2-\underline{CH_3}$). ^{13}C NMR (126 MHz, $CDCl_3$) δ 197.42, 174.42, 166.95, 164.90, 66.79 (2), 63.66 (2), 52.83 (2), 50.90, 49.61, 43.55, 37.39, 32.00, 29.74, 29.72, 29.59, 29.44, 29.30, 29.14, 28.70, 22.78, 14.24. HRMS (ESI): Calculated for $C_{25}H_{46}N_7OS_2$ $[M+H]^+$: 524.31998, found: 524.31848.

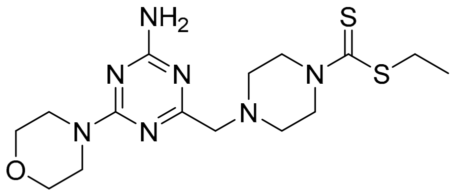

Ethyl-4-((4-amino-6-morpholino-1,3,5-triazin-2-yl)methyl)piperazine-1-carbodithioate (C25): Yield: 62.33%; white solid; m. p. 114.1-114.7 °C; ^1H NMR (500 MHz, DMSO-d_6) δ 6.90 (d, J = 66.0 Hz, 2H, $-NH_2$), 4.06 (d, J = 172.4 Hz, 4H, Piperazine-H), 3.68 – 3.64 (m, 4H, Morpholine-H), 3.61 – 3.58 (m, 4H, $-CH_2-O-CH_2-$), 3.31 (s, 2H, $-S-CH_2$), 3.21 (d, J = 7.3 Hz, 2H, $-CH_2-N-$), 2.63 (t, J = 5.2 Hz, 4H, Piperazine-H), 1.25 (t, J = 7.4 Hz, 3H, $-CH_2-\underline{CH_3}$). ^{13}C NMR (126 MHz, DMSO-d_6) δ 195.57, 174.19, 167.29, 165.09, 66.46 (2), 63.44 (2), 52.50 (2), 51.32, 50.16, 43.58, 31.02, 14.40. HRMS (ESI): Calculated for $C_{15}H_{26}N_7OS_2$ $[M+H]^+$: 384.16348, found: 384.16171.

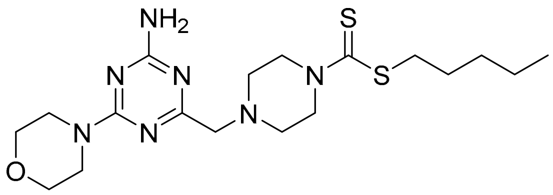

Pentyl-4-((4-amino-6-morpholino-1,3,5-triazin-2-yl)methyl)piperazine-1-carbodithioate (C26): Yield: 55.17%; white solid; m. p. 84.1-84.9 °C; ^{1}H NMR (500 MHz, $CDCl_3$) δ 5.15 (s, 2H, $-NH_2$), 4.19 (d, *J* = 207.6 Hz, 4H, Piperazine-H), 3.76 (s, 4H, Morpholine-H), 3.70 – 3.67 (m, 4H, $-CH_2$-O-CH_2-), 3.41 (s, 2H, -S-CH_2), 3.29 – 3.24 (m, 2H, $-CH_2$-N-), 2.66 (s, 4H, Piperazine-H), 1.67 (p, *J* = 7.4 Hz, 2H, -S-CH_2-$\underline{CH_2}$-), 1.40 – 1.28 (m, 4H, -$(\underline{CH_2})_2$-CH_3), 0.87 (t, *J* = 7.2 Hz, 3H, $-CH_2$-$\underline{CH_3}$). ^{13}C NMR (126 MHz, $CDCl_3$) δ 197.41, 174.49, 167.00, 164.92, 66.80 (2), 63.70 (2), 52.84 (2), 50.92, 49.62, 43.54, 37.35, 31.27, 28.41, 22.39, 14.09. HRMS (ESI): Calculated for $C_{18}H_{32}N_7OS_2$ [M+H]$^+$: 426.21043, found: 426.20877.

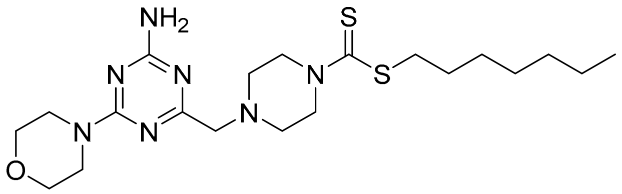

Heptyl-4-((4-amino-6-morpholino-1,3,5-triazin-2-yl)methyl)piperazine-1-carbodithioate (C27): Yield: 64.05%; white solid; m. p. 69.6-69.9 °C; ^{1}H NMR (500 MHz, $CDCl_3$) δ 5.15 (s, 2H, $-NH_2$), 4.20 (d, *J* = 207.4 Hz, 4H, Piperazine-H), 3.76 (s, 4H, Morpholine-H), 3.69 (dd, *J* = 5.7, 3.8 Hz, 4H, $-CH_2$-O-CH_2-), 3.41 (s, 2H, -S-CH_2), 3.31 – 3.23 (m, 2H, $-CH_2$-N-), 2.67 (s, 4H, Piperazine-H), 1.71 – 1.63 (m, 2H, -S-CH_2-$\underline{CH_2}$-), 1.44 – 1.33 (m, 2H, -$\underline{CH2}$-$(CH_2)_3$-CH_3), 1.32 – 1.22 (m, 6H, -$(\underline{CH_2})_3$-CH_3), 0.85 (t, *J* = 6.9 Hz, 3H, $-CH_2$-$\underline{CH_3}$). ^{13}C NMR (126 MHz, $CDCl_3$) δ 197.43, 174.47, 166.97, 164.92, 66.80 (2), 63.69 (2), 52.84 (2), 50.92, 49.62, 43.56, 37.40, 31.79, 29.09, 28.97, 28.71, 22.69, 14.20.

HRMS (ESI): Calculated for $C_{20}H_{36}N_7OS_2$ $[M+H]^+$: 454.24173, found: 454.239 93.

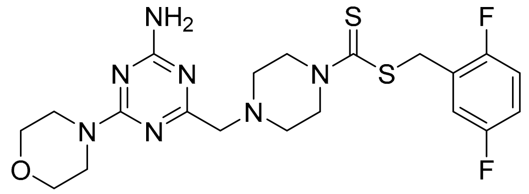

2,5-difluorobenzyl-4-((4-amino-6-morpholino-1,3,5-triazin-2-yl)methyl)piperazine-1-carbodithioate (C28): Yield: 67.28%; white solid; m. p. 94.4-95.0 °C; ^{1}H NMR (500 MHz, $CDCl_3$) δ 7.25 – 7.20 (m, 1H, Ar-H), 6.87 (t, J = 7.8 Hz, 2H, Ar-H), 5.23 (s, 2H, $-NH_2$), 4.54 (s, 2H, $-S-CH_2$), 4.16 (d, J = 239.9 Hz, 4H, Piperazine-H), 3.75 (s, 4H, Morpholine-H), 3.68 (dd, J = 5.6, 3.9 Hz, 4H, $-CH_2-O-CH_2-$), 3.40 (s, 2H, $-CH_2-N-$), 2.66 (s, 4H, Piperazine-H). ^{13}C NMR (126 MHz, $CDCl_3$) δ 195.75, 174.31, 166.87, 164.86, 129.70, 129.62, 111.59, 111.55, 111.43, 111.39, 111.34, 66.77 (2), 63.55 (2), 52.72 (2), 50.92, 49.74, 2 9.54. HRMS (ESI): Calculated for $C_{18}H_{32}N_7OS_2$ $[M+H]^+$: 426.21043, found: 42 6.20877.

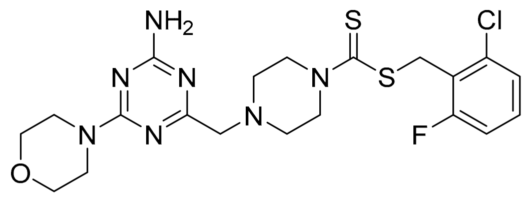

3-chlorobenzyl-4-((4-amino-6-morpholino-1,3,5-triazin-2-yl)methyl)piperazine-1-carbodithioate (C29): Yield: 67.84%; white solid; m. p. 136.0-136.3 °C; ^{1}H NMR (500 MHz, DMSO-d_6) δ 7.41 – 7.32 (m, 2H, Ar-H), 7.24 (dt, J = 11.3, 5.4 Hz, 1H, Ar-H), 6.85 (d, J = 64.5 Hz, 2H, $-NH_2$), 4.48 (d, J = 3.6 Hz, 2H, $-S-CH_2$), 3.99 (d, J = 197.0 Hz, 4H, Piperazine-H), 3.61 (s, 4H, Morpholine-H), 3.54 (d, J = 5.6 Hz, 4H, $-CH_2-O-$

CH_2-), 3.27 (d, J = 3.4 Hz, 2H, -CH_2-N-), 2.59 (d, J = 20.8 Hz, 4H, Piperazine-H). ^{13}C NMR (126 MHz, DMSO-d_6) δ 193.90, 174.21, 167.30, 165.09, 162.56, 160.58, 135.44, 135.40, 131.39, 131.32, 126.30, 126.27, 121.38, 121.23, 115.37, 115.19, 66.46 (2), 63.35 (2), 52.42 (2), 51.47, 50.36, 43.60, 33.46. HRMS (ESI): Calculated for $C_{20}H_{26}ClFN_7OS_2$ $[M+H]^+$: 498.13073, found: 498.12888.

$C_{20}H_{27}ClN_7OS_2$

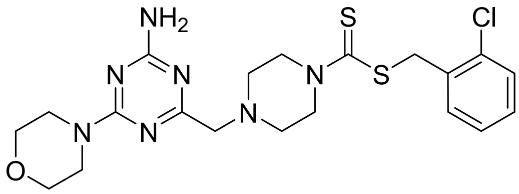

2-chlorobenzyl-4-((4-amino-6-morpholino-1,3,5-triazin-2-yl)methyl)piperazine-1-carbodithioate (C30): Yield: 61.28%; white solid; m. p. 121.3-121.6 °C; ^1H NMR (500 MHz, DMSO-d_6) δ 7.42 (s, 1H, Ar-H), 7.34 – 7.24 (m, 3H, Ar-H), 6.84 (d, J = 61.0 Hz, 2H, -NH_2), 4.52 (s, 2H, -S-CH_2), 4.02 (d, J = 172.5 Hz, 4H, Piperazine-H), 3.61 (s, 4H, Morpholine-H), 3.54 (d, J = 5.1 Hz, 4H, -CH_2-O-CH_2-), 3.27 (s, 2H, -CH_2-N-), 2.59 (s, 4H, Piperazine-H). ^{13}C NMR (126 MHz, DMSO-d_6) δ 194.59, 174.18, 167.30, 165.09, 139.90, 133.44, 130.82, 129.42, 128.43, 127.80, 66.46 (2), 63.40 (2), 52.51 (2), 51.85, 50.36, 43.60, 40.46. HRMS (ESI): Calculated for $C_{20}H_{27}ClN_7OS_2$ $[M+H]^+$: 480.14015, found: 480.13837.

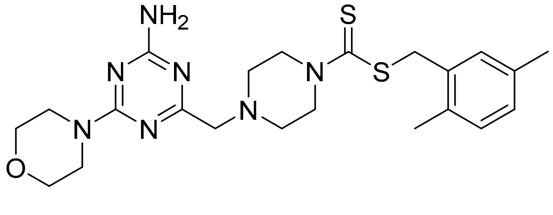

2,5-dimethylbenzyl-4-((4-amino-6-morpholino-1,3,5-triazin-2-yl)methyl)piperazine-1-carbodithioate (C31): Yield: 79.64%; white solid; m. p. 192.2-193.0 °C; ^{1}H NMR (500 MHz, DMSO-*d*6) δ 7.05 (dd, *J* = 8.4, 6.4 Hz, 1H, Ar-H), 6.99 (d, *J* = 7.4 Hz, 2H, Ar-H), 6.86 (d, *J* = 67.9 Hz, 2H, $-NH_2$), 4.33 (s, 2H, $-S-CH_2$), 4.00 (d, *J* = 201.2 Hz, 4H, Piperazine-H), 3.64 – 3.59 (m, 4H, Morpholine-H), 3.56 – 3.53 (m, 4H, $-CH_2-O-CH_2-$), 3.27 (s, 2H, $-CH_2-N-$), 2.58 (d, *J* = 27.3 Hz, 4H, Piperazine-H), 2.26 (s, 6H, $Ar-CH_3$). ^{13}C NMR (126 MHz, DMSO-*d*6) δ 195.25, 174.20, 167.29, 165.09, 137.98 (2), 131.35, 128.66 (2), 128.24, 66.46 (2), 63.44 (2), 52.49 (2), 51.18, 50.22, 43.60, 37.78, 19.89 (2). HRMS (ESI): Calculated for $C_{22}H_{32}N_7OS_2$ $[M+H]^+$: 474.21043, found: 474.20853.

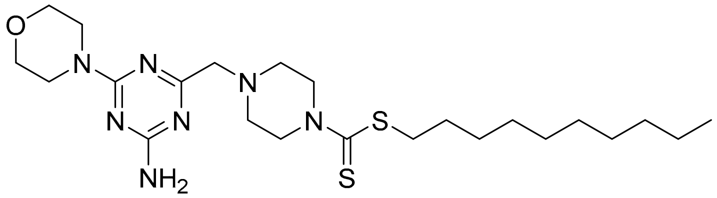

Decyl-4-((4-amino-6-morpholino-1,3,5-triazin-2-yl)methyl)piperazine-1-carbodit hioate (32): Yield: 47.51%; white solid; m. p. 83.2-84.1 °C; ^{1}H NMR (500 M Hz, $CDCl_3$) δ 5.15 (d, *J* = 84.3 Hz, 2H, $-NH_2$), 4.20 (d, *J* = 206.8 Hz, 4H, P iperazine-H), 3.77 (s, 4H, Morpholine-H), 3.69 (dd, *J* = 5.7, 3.8 Hz, 4H, $-CH_2$ $-O-CH_2-$), 3.41 (d, *J* = 1.0 Hz, 2H, $-S-CH_2$), 3.26 (dd, *J* = 8.1, 6.9 Hz, 2H, -CH_2-N-), 2.67 (s, 4H, Piperazine-H), 1.69 – 1.63 (m, 2H, $-S-CH_2-\underline{CH_2}-$), 1.38 (t, *J* = 7.7 Hz, 2H, $-\underline{CH2}-(CH_2)_6-$), 1.24 (d, *J* = 6.2 Hz, 12H, $-(\underline{CH_2})_6-CH_3$), 0. 86 – 0.83 (m, 3H, $-CH_2-\underline{CH_3}$). ^{13}C NMR (126 MHz, $CDCl_3$) δ 197.44, 174.26, 166.73, 164.81, 66.78 (2), 63.57 (2), 52.83 (2), 50.89, 49.59, 43.58, 37.40, 31.

98, 29.63, 29.59, 29.40, 29.30, 29.14, 28.70, 22.77, 14.23. HRMS (ESI): Calculated for $C_{23}H_{42}N_7OS_2$ $[M+H]^+$: 496.28868, found: 496.28705.

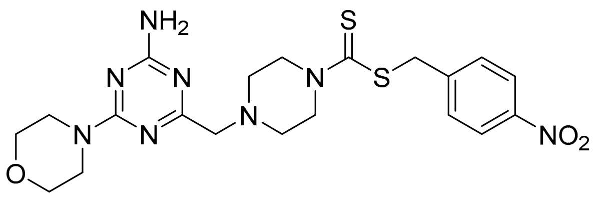

4-nitrobenzyl-4-((4-amino-6-morpholino-1,3,5-triazin-2-yl)methyl)piperazine-1-carbodithioate (C33): Yield: 85.74%; white solid; m. p. 149.8-150.4 °C; ^1H NMR (500 MHz, DMSO-d_6) δ 8.18 (d, J = 8.6 Hz, 2H, Ar-H), 7.66 (d, J = 8.6 Hz, 2H, Ar-H), 6.91 (d, J = 61.4 Hz, 2H, -NH_2), 4.73 (s, 2H, -S-CH_2), 4.08 (d, J = 164.0 Hz, 4H, Piperazine-H), 3.66 (s, 4H, Morpholine-H), 3.59 (s, 4H, -CH_2-O-CH_2-), 3.32 (s, 2H, -CH_2-N-), 2.64 (s, 4H, Piperazine-H). HRMS (ESI): Calculated for $C_{20}H_{27}N_8O_3S_2$ $[M+H]^+$: 491.16420, found: 491.16257. ^{13}C NMR (126 MHz, $CDCl_3$) δ 194.98, 174.30, 166.95, 164.88, 147.19, 144.78, 130.22 (2), 123.79 (2), 66.77 (2), 63.59 (2), 52.77 (2), 51.63, 49.89, 43.55, 40.57. HRMS (ESI): Calculated for $C_{23}H_{42}N_7OS_2$ $[M+H]^+$: 496.28868, found: 496.28705.

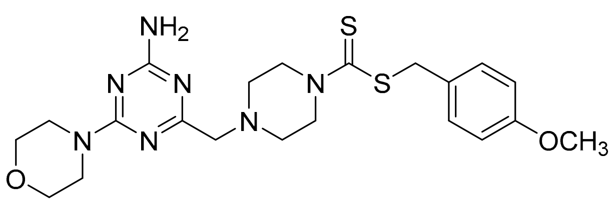

4-methoxybenzyl-4-((4-amino-6-morpholino-1,3,5-triazin-2-yl)methyl)piperazine-1-carbodithioate (C34): Yield: 61.32%; white solid; m. p. 113.4-114.2 °C; ^1H NMR (500 MHz, DMSO-d_6) δ 7.29 – 7.23 (m, 2H, Ar-H), 6.84 – 6.81 (m, 2

H, Ar-H), 4.40 (d, J = 3.6 Hz, 2H, $-NH_2$), 4.19 (s, 4H, Piperazine-H), 3.68 (d, J = 3.5 Hz, 2H $-S-CH_2$), 3.61 (s, 4H, Morpholine-H), 3.54 (d, J = 5.3 Hz, 4 H, $-CH_2-O-CH_2-$), 3.28 (s, 2H, $-CH_2-N-$), 2.59 (s, 4H, Piperazine-H). ^{13}C NMR (126 MHz, DMSO-d_6) δ 195.25, 174.10, 167.25, 165.06, 159.15, 131.00 (2), 128.21, 114.45 (2), 66.45 (2), 63.28 (2), 55.62 (2), 52.48, 51.34, 50.19, 43.59, 41.02. HRMS (ESI): Calculated for $C_{21}H_{30}N_7O_2S_2$ $[M+H]^+$: 476.18969, found: 4 76.18790.

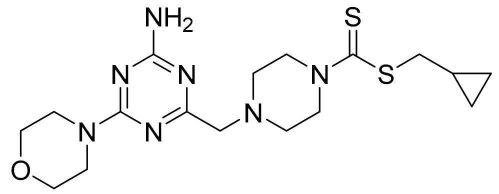

Cyclopropylmethyl-4-((4-amino-6-morpholino-1,3,5-triazin-2-yl)methyl)piperazine-1-carbodithioate (C35): Yield: 71.40%; white solid; m. p. 104.2-105.1 °C; ^1H NMR (500 MHz, DMSO-d_6) δ 6.91 (d, J = 65.6 Hz, 2H, $-NH_2$), 4.07 (d, J = 160.5 Hz, 4H, Piperazine-H), 3.66 (s, 4H, Morpholine-H), 3.60 (d, J = 4. 8 Hz, 4H, $-CH_2-O-CH_2-$), 3.32 (s, 2H, $-S-CH_2$), 3.18 (d, J = 7.3 Hz, 2H, $-CH_2-N-$), 2.66 – 2.61 (m, 4H, Piperazine-H), 1.11 (s, 1H, $-\underline{CH}-(CH_2)_2$), 0.55 – 0.5 1 (m, 2H, $-\underline{CH_2}-CH_2-$), 0.31 – 0.27 (m, 2H, $-CH_2-\underline{CH_2}-$). ^{13}C NMR (126 MHz, DMSO) δ 195.80, 174.11, 167.21, 165.01, 66.3 (2)9, 63.37 (2), 52.46 (2), 51.2 5, 50.13, 43.53, 42.85, 10.19, 6.10. HRMS (ESI): Calculated for $C_{17}H_{28}N_7OS_2$ $[M+H]^+$: 410.17913, found: 410.17749.

1. ¹H, ¹³C, ¹⁹F NMR, HRMS data

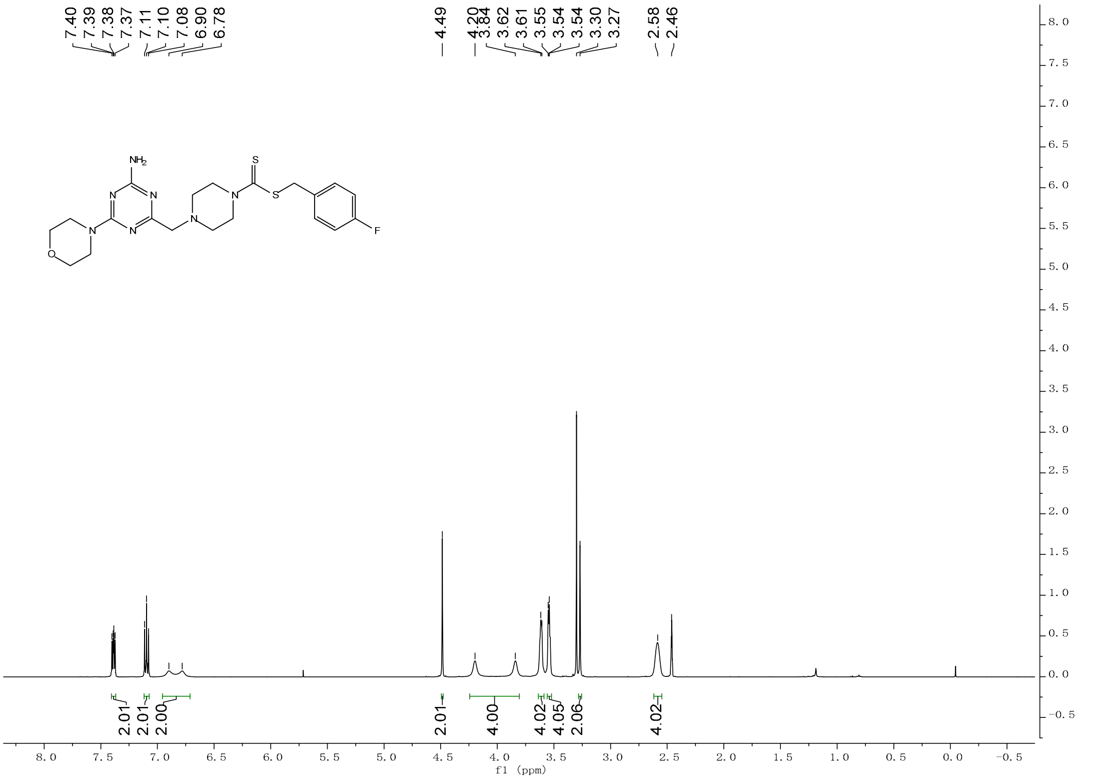

Figure S1. ¹H NMR (500 MHz, DMSO-d_6) spectrum of compound **C1**.

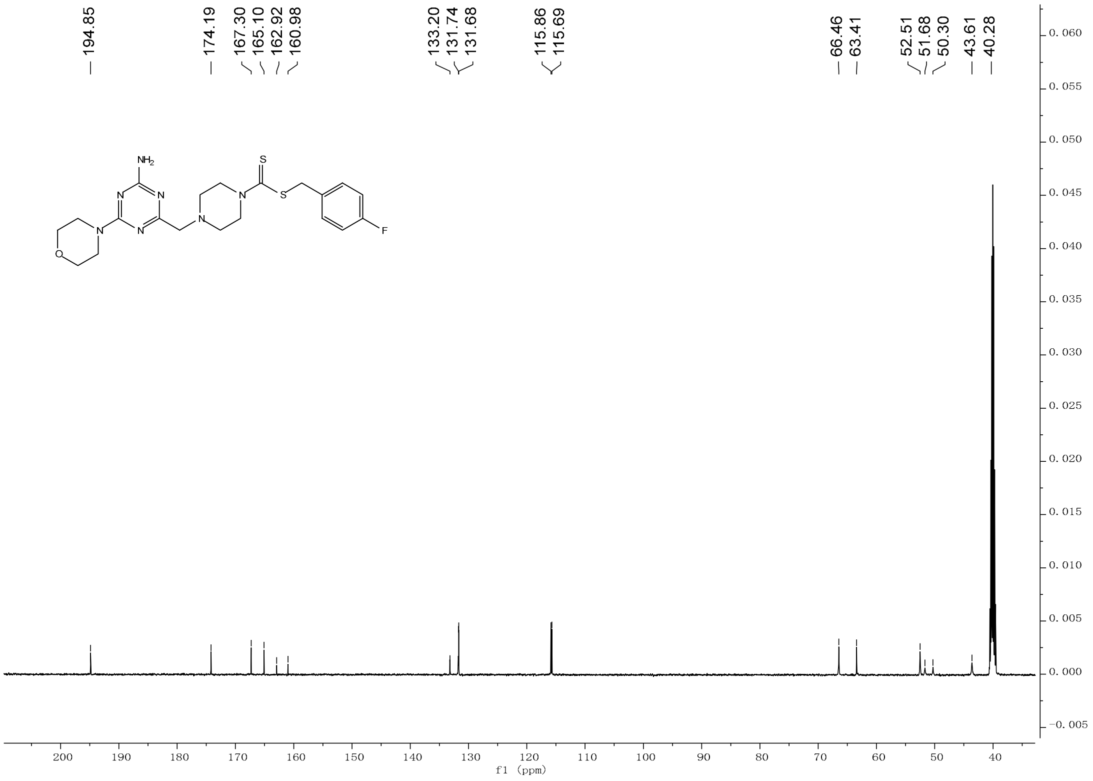

Figure S2. ¹³C NMR (126 MHz, DMSO-d_6) spectrum of compound **C1**.

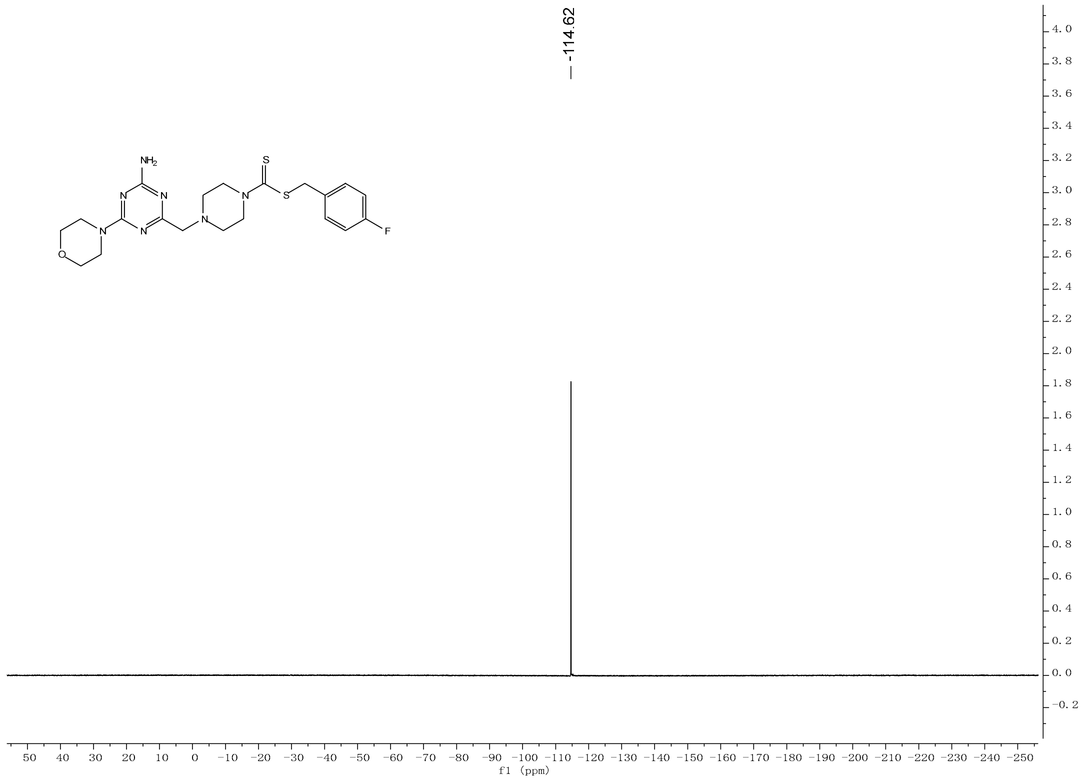

Figure S3. ^{19}F NMR (471 MHz, $CDCl_3$) spectrum of compound **C1**

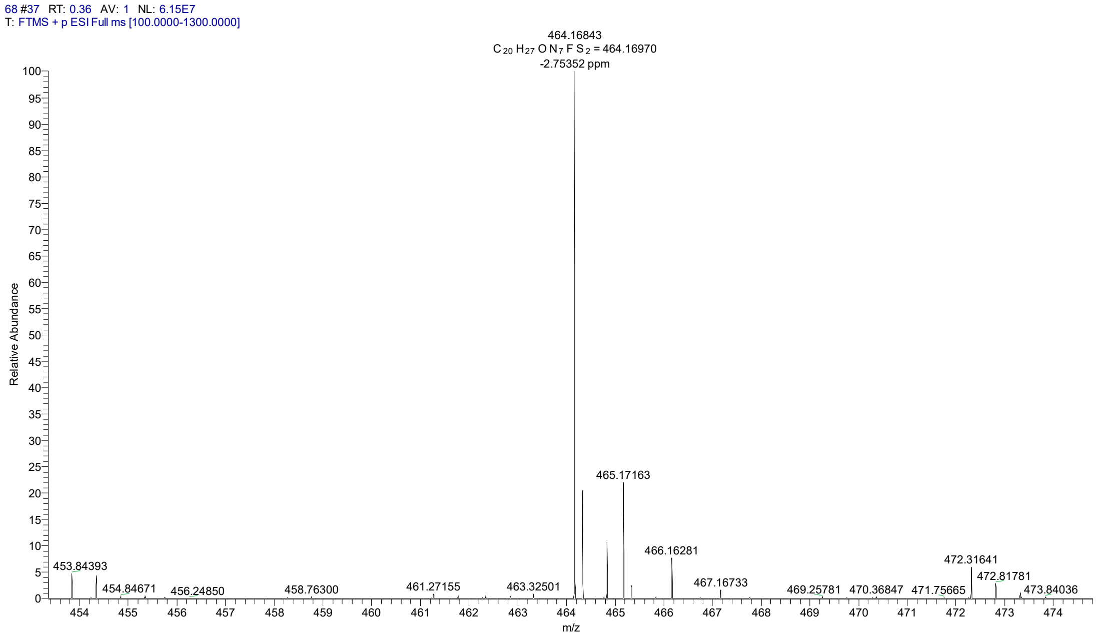

Figure S4. HRMS of compound **C1**.

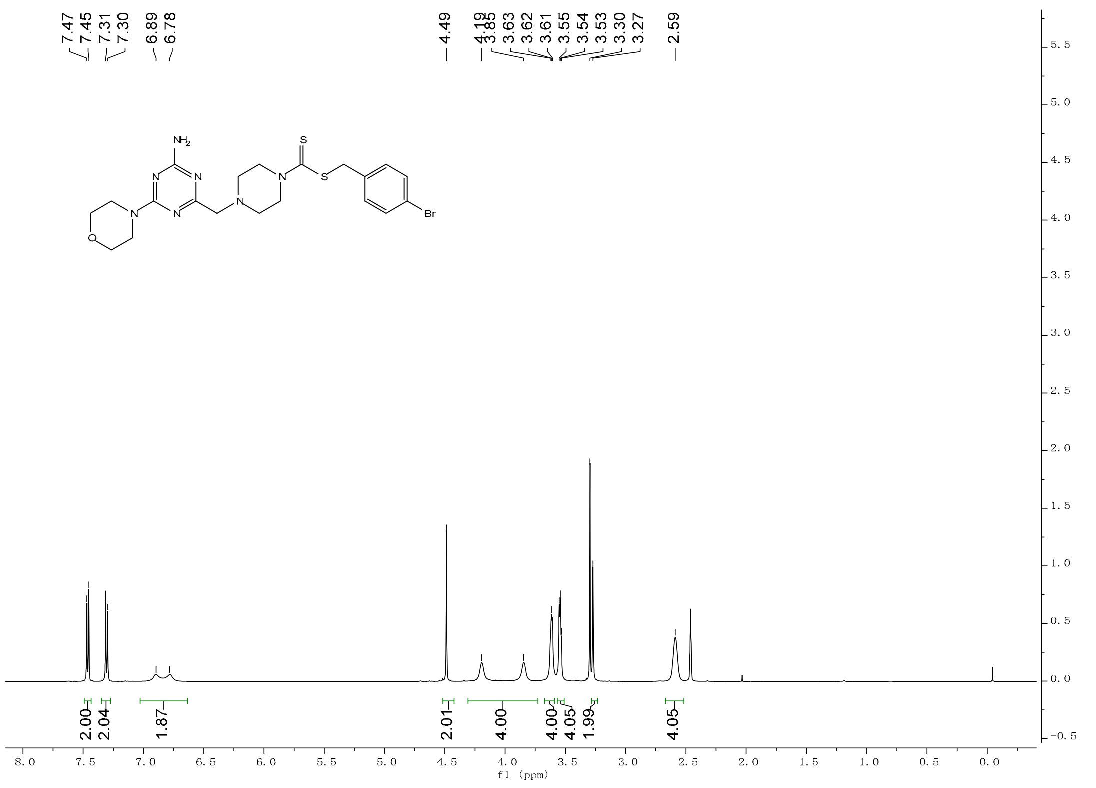

Figure S5. ^{1}H NMR (500 MHz, DMSO-d_6) spectrum of compound **C2**.

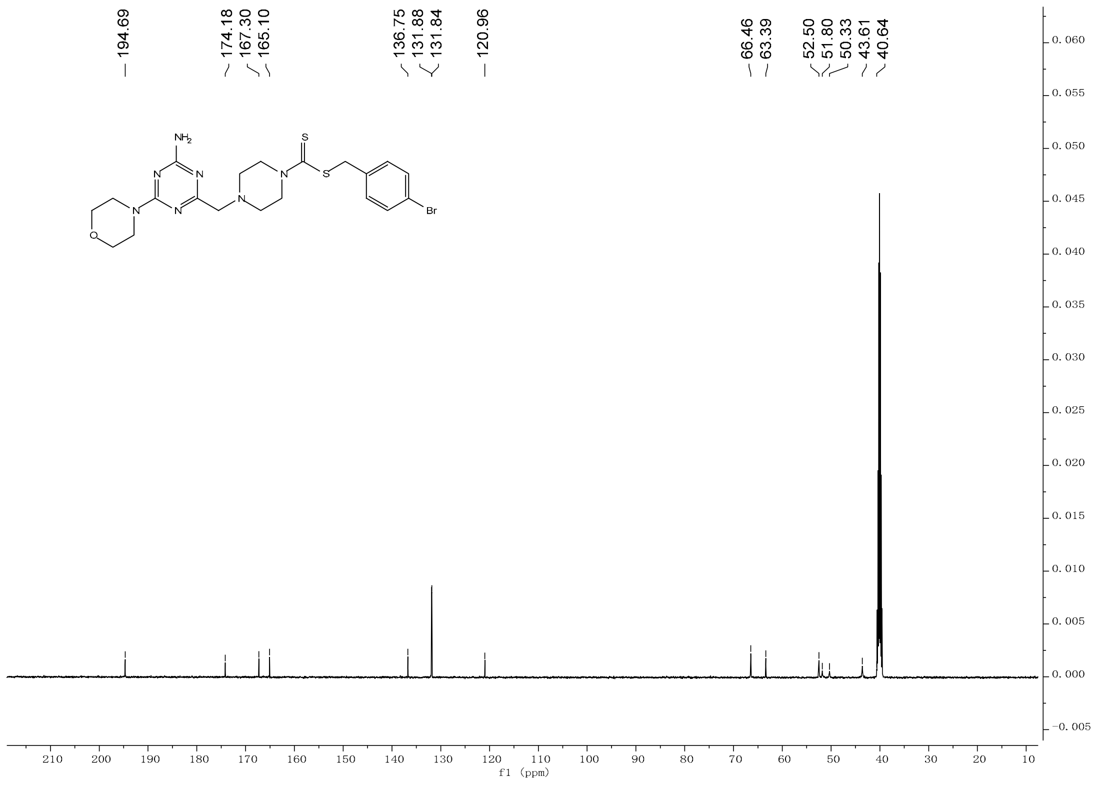

Figure S6. ^{13}C NMR (126 MHz, DMSO-d_6) spectrum of compound **C2**.

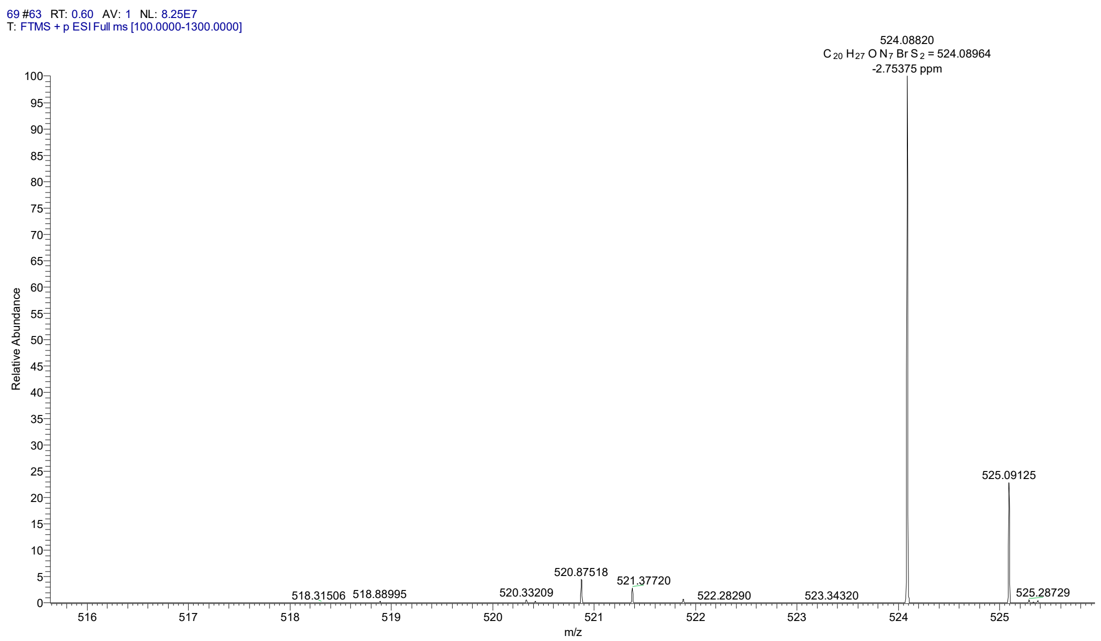

Figure S7. HRMS of compound **C2**.

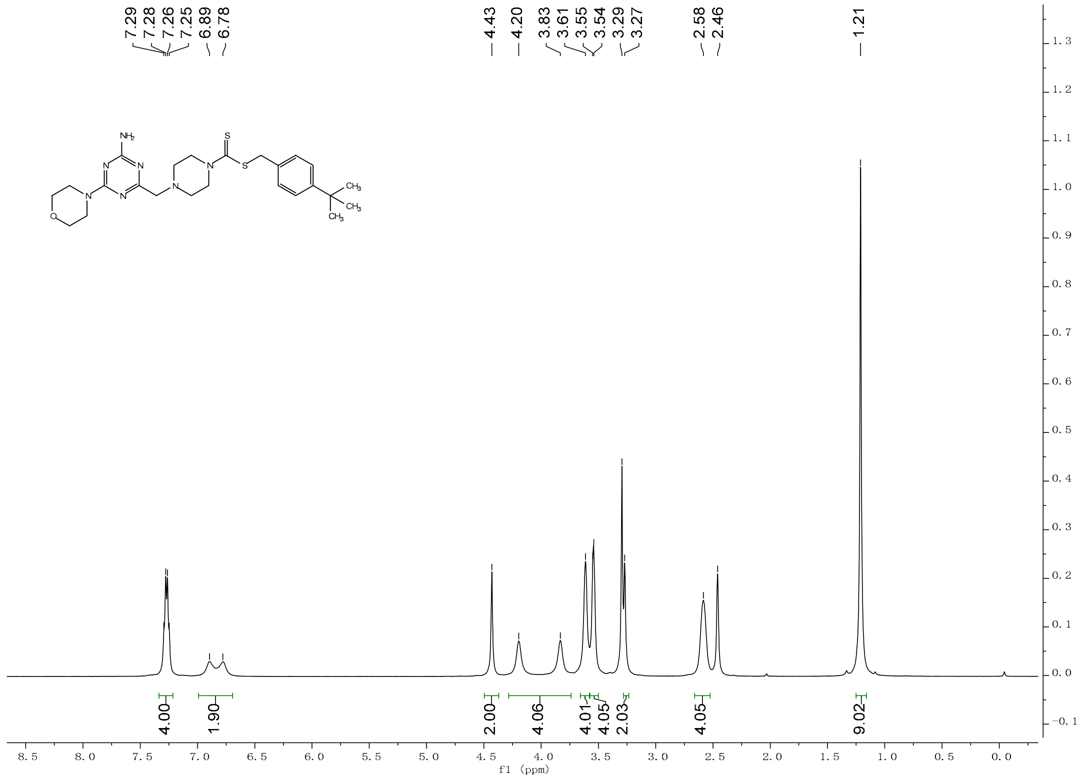

Figure S8. ^{1}H NMR (500 MHz, DMSO-d_6) spectrum of compound **C3**.

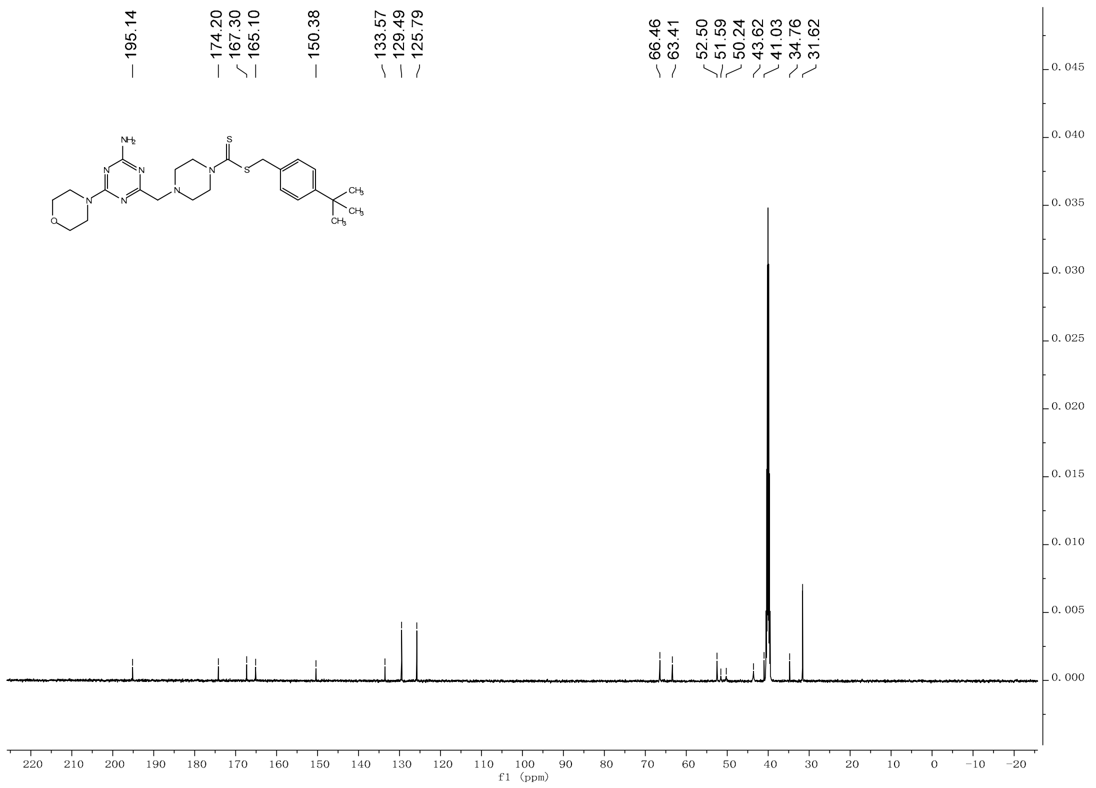

Figure S9. ^{13}C NMR (126 MHz, DMSO-d_6) spectrum of compound **C3**.

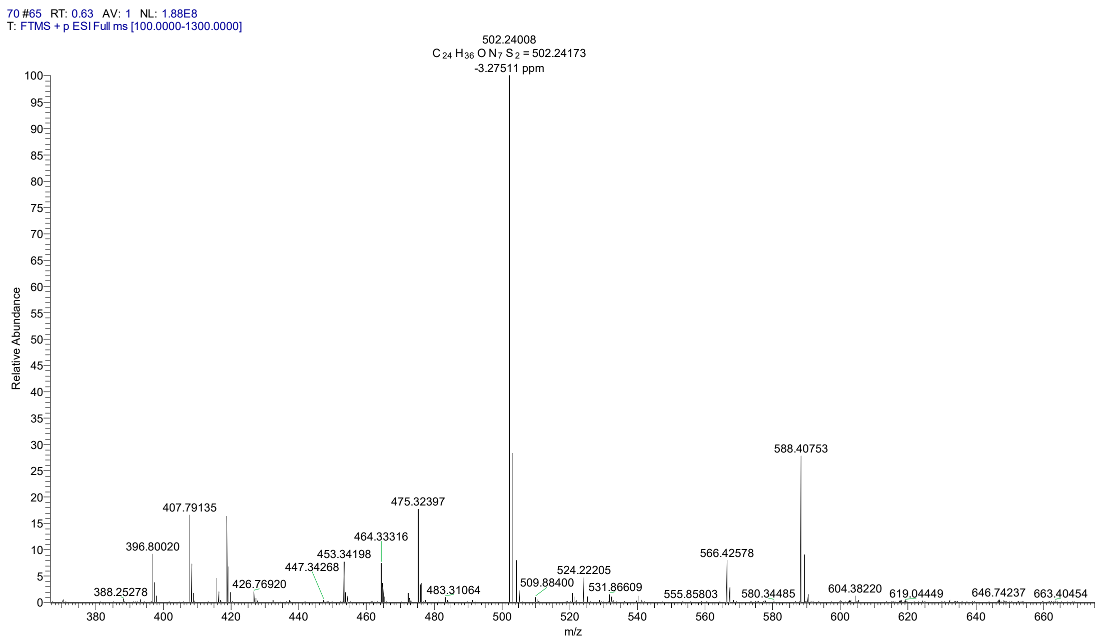

Figure S10. HRMS of compound **C3**.

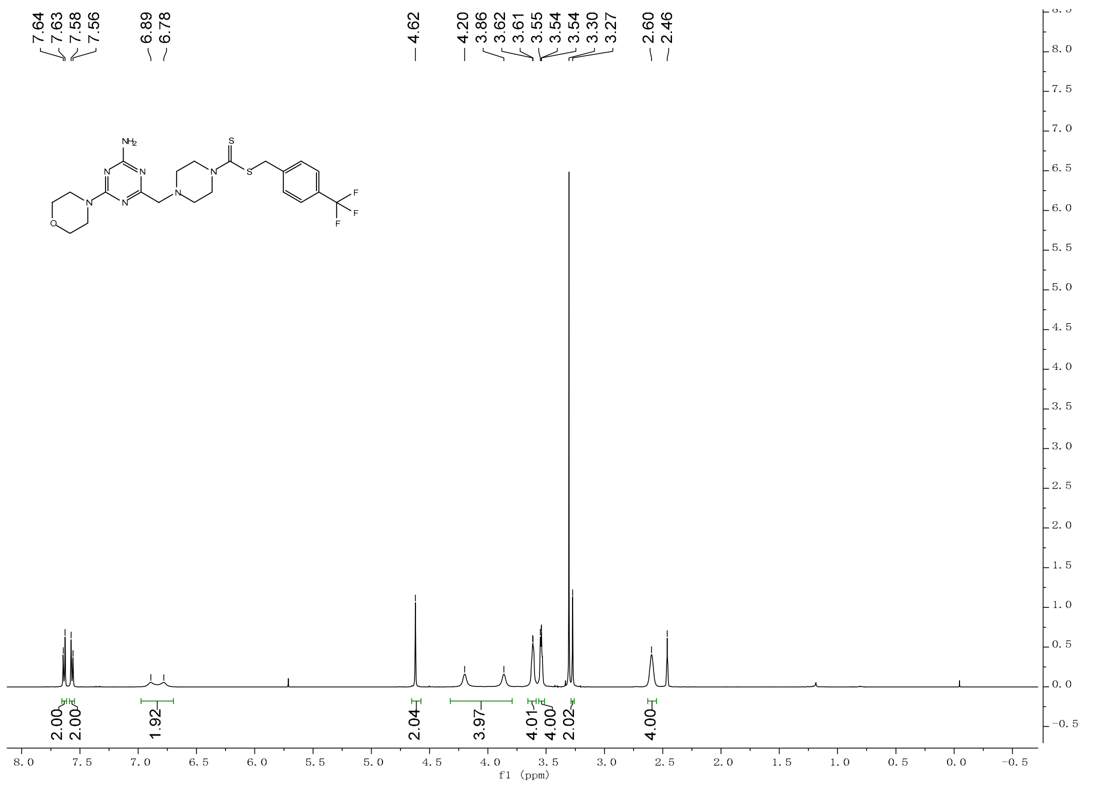

Figure S11. ^{1}H NMR (500 MHz, DMSO-d_6) spectrum of compound **C4**.

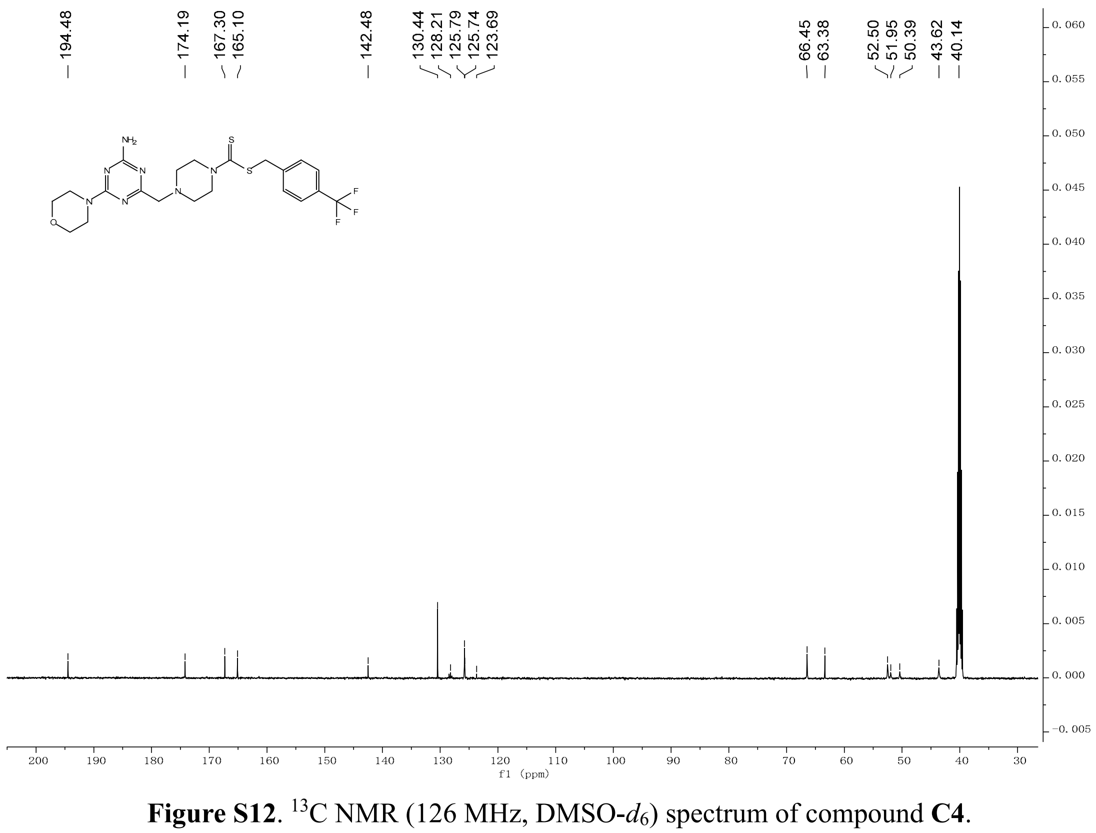

Figure S12. ^{13}C NMR (126 MHz, DMSO-d_6) spectrum of compound **C4**.

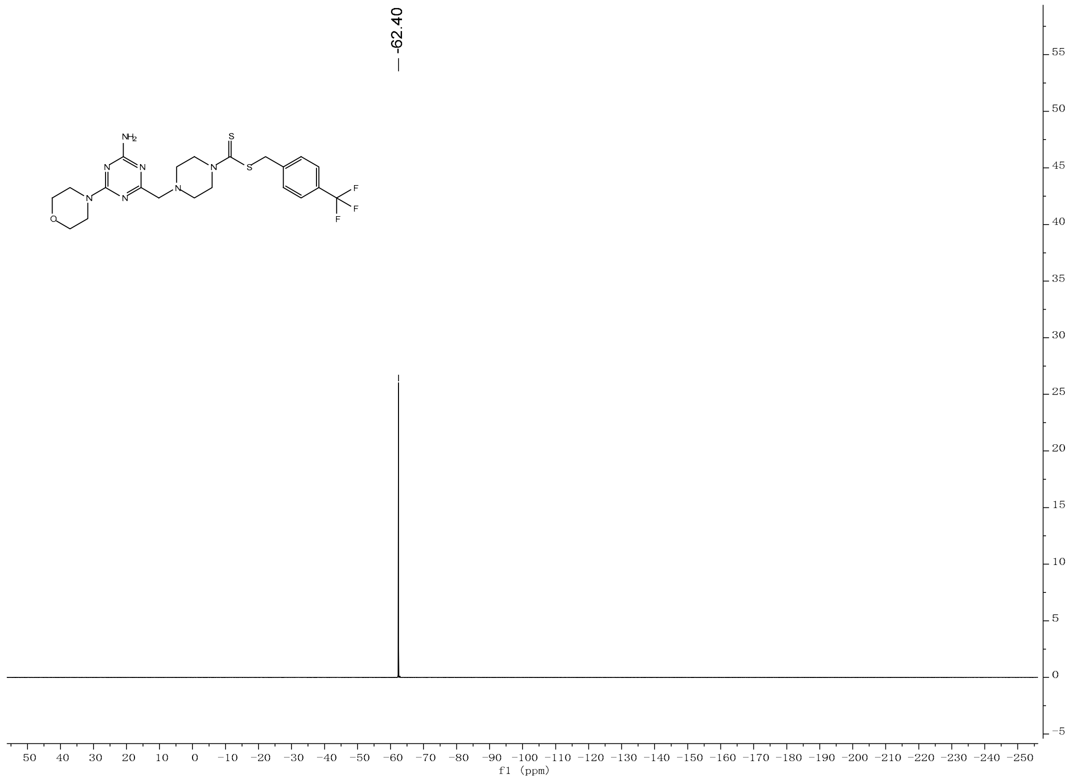

Figure S13. ^{19}F NMR (471 MHz, $CDCl_3$) spectrum of compound **C4**.

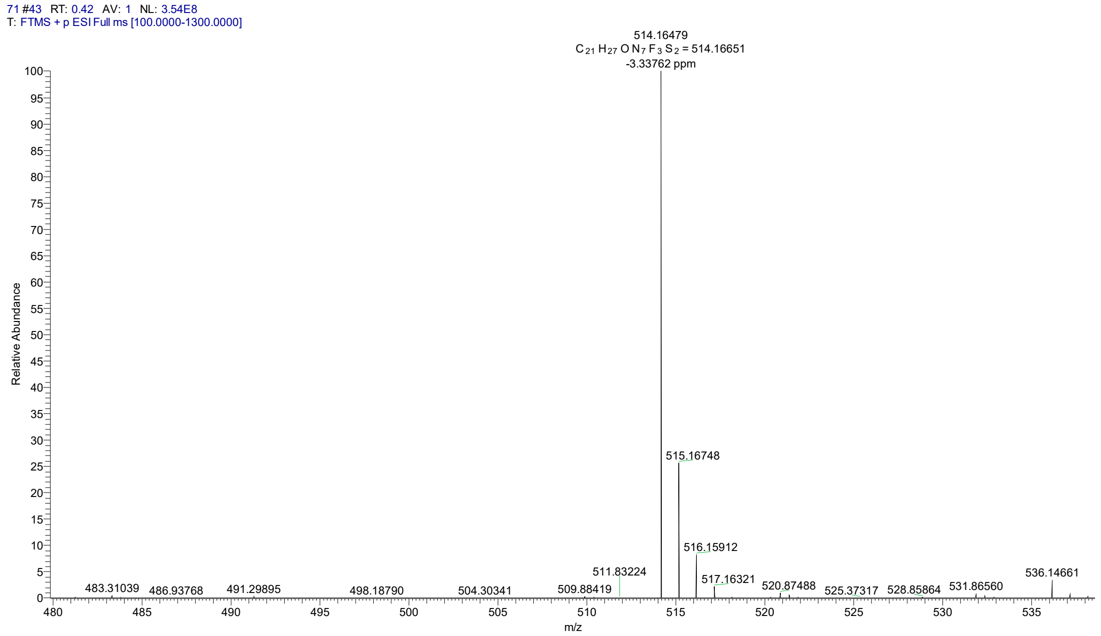

Figure S14. HRMS of compound **C4**.

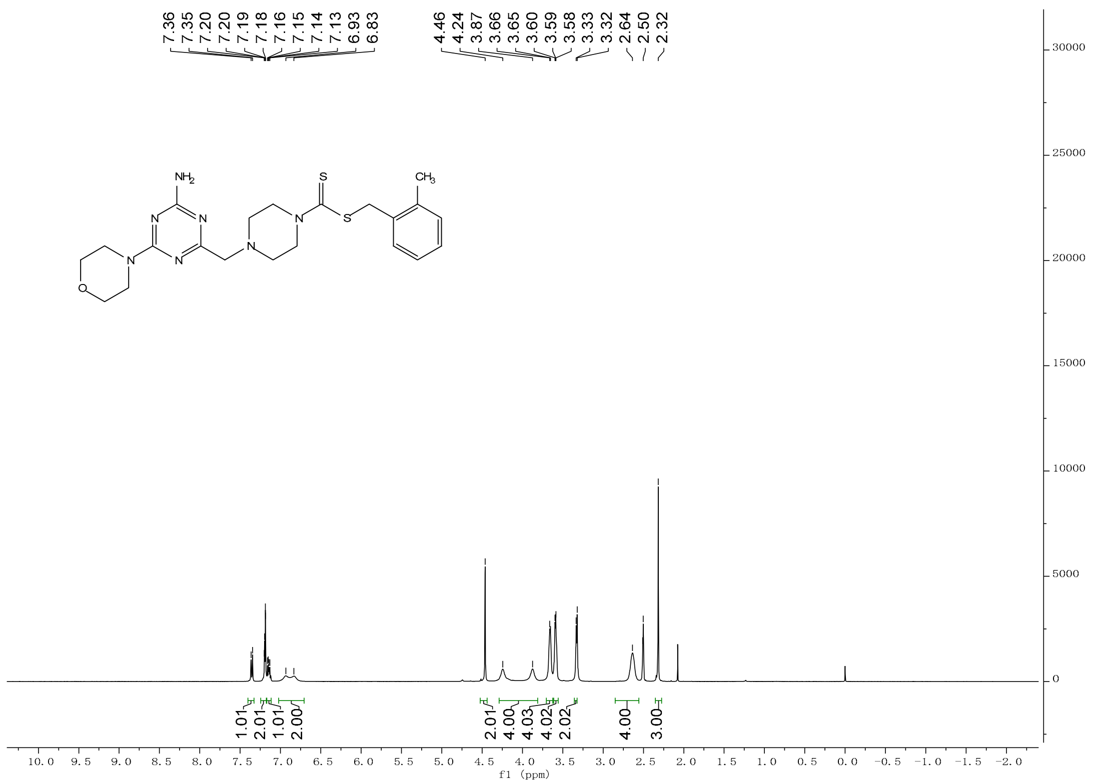

Figure S15. ^{1}H NMR (500 MHz, DMSO-d_6) spectrum of compound **C5**.

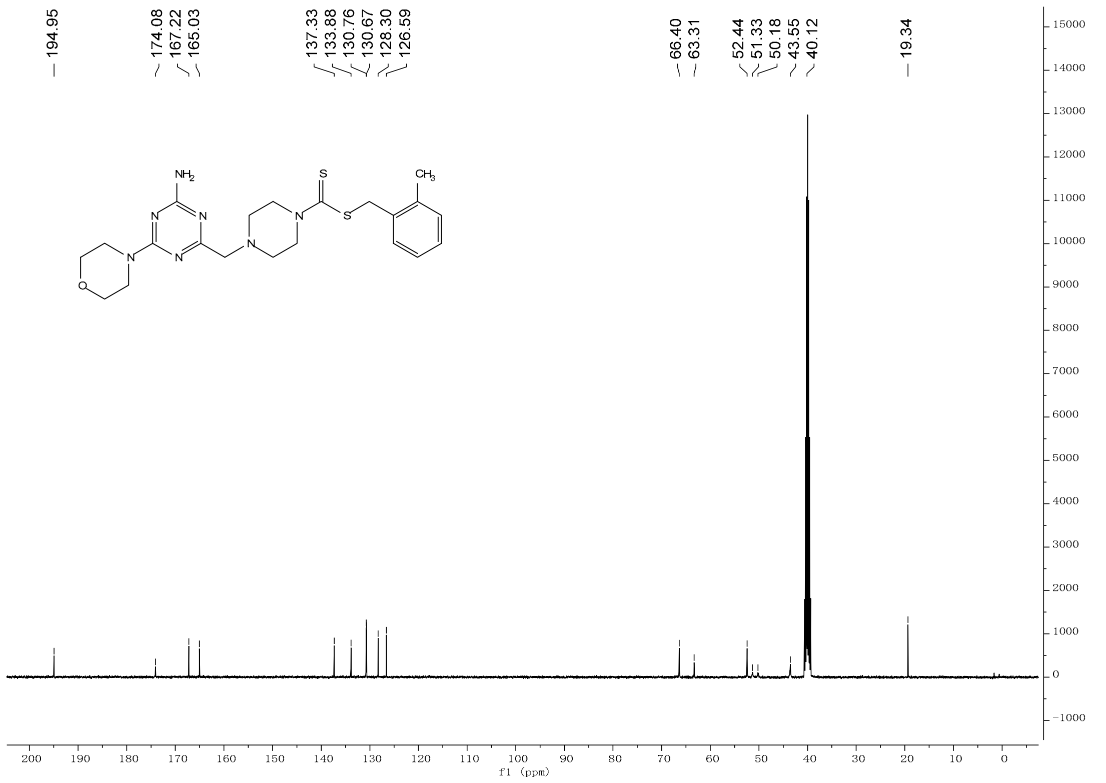

Figure S16. ^{13}C NMR (101 MHz, DMSO-d_6) spectrum of compound **C5**.

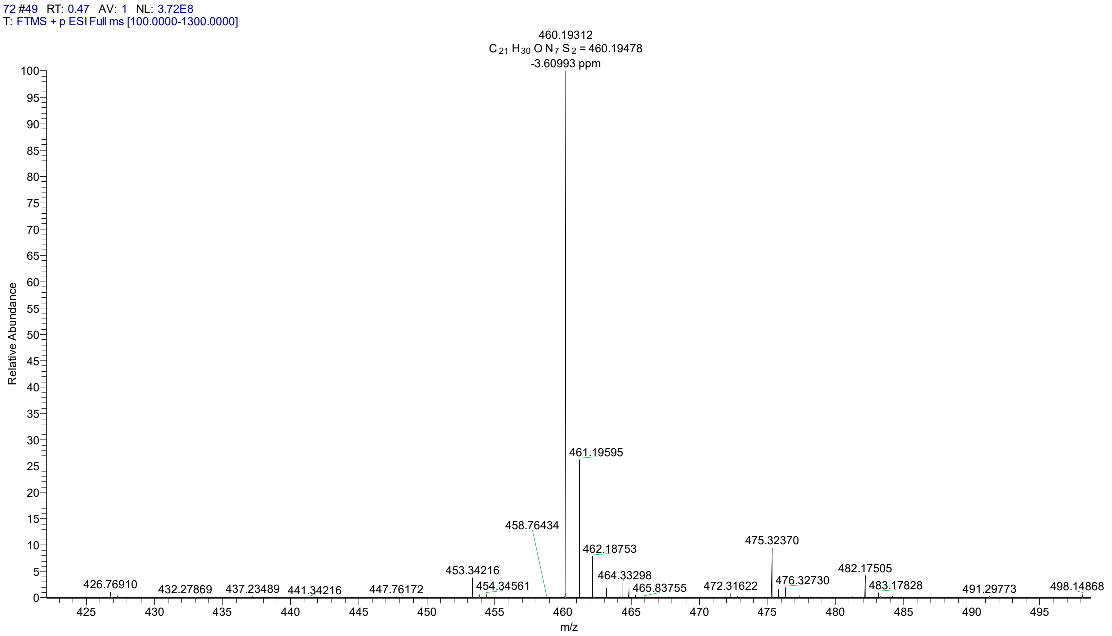

Figure S17. HRMS of compound **C5**.

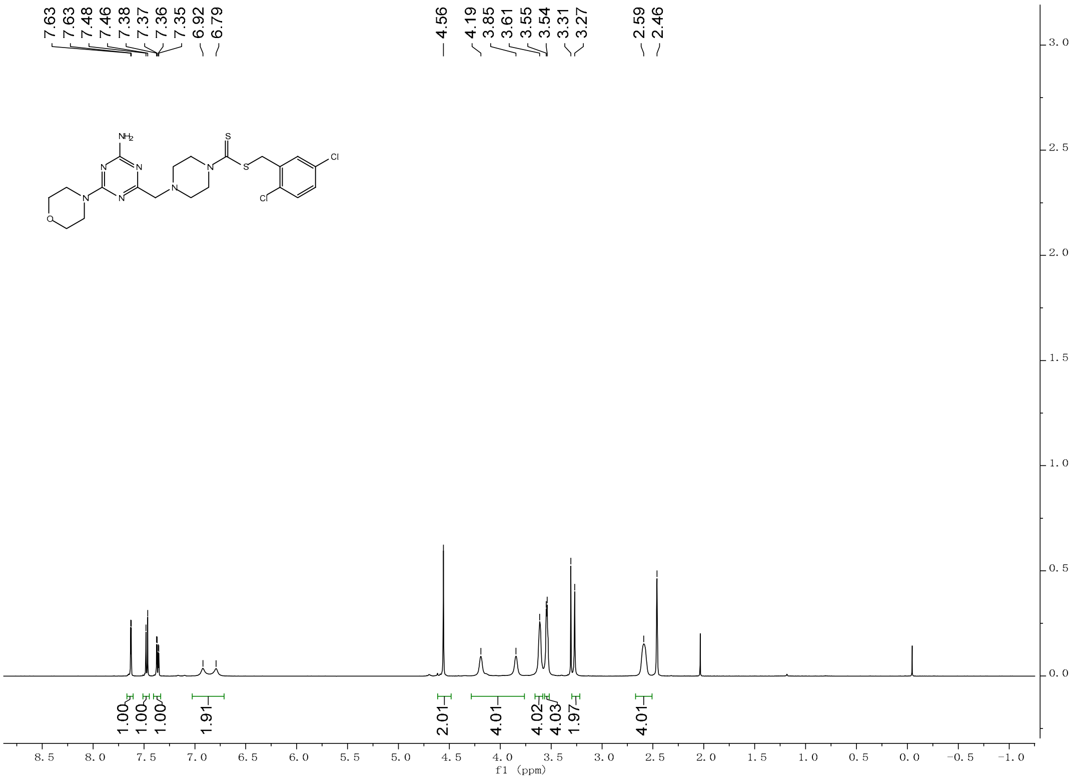

Figure S18. ^{1}H NMR (500 MHz, DMSO-d_6) spectrum of compound **C6**.

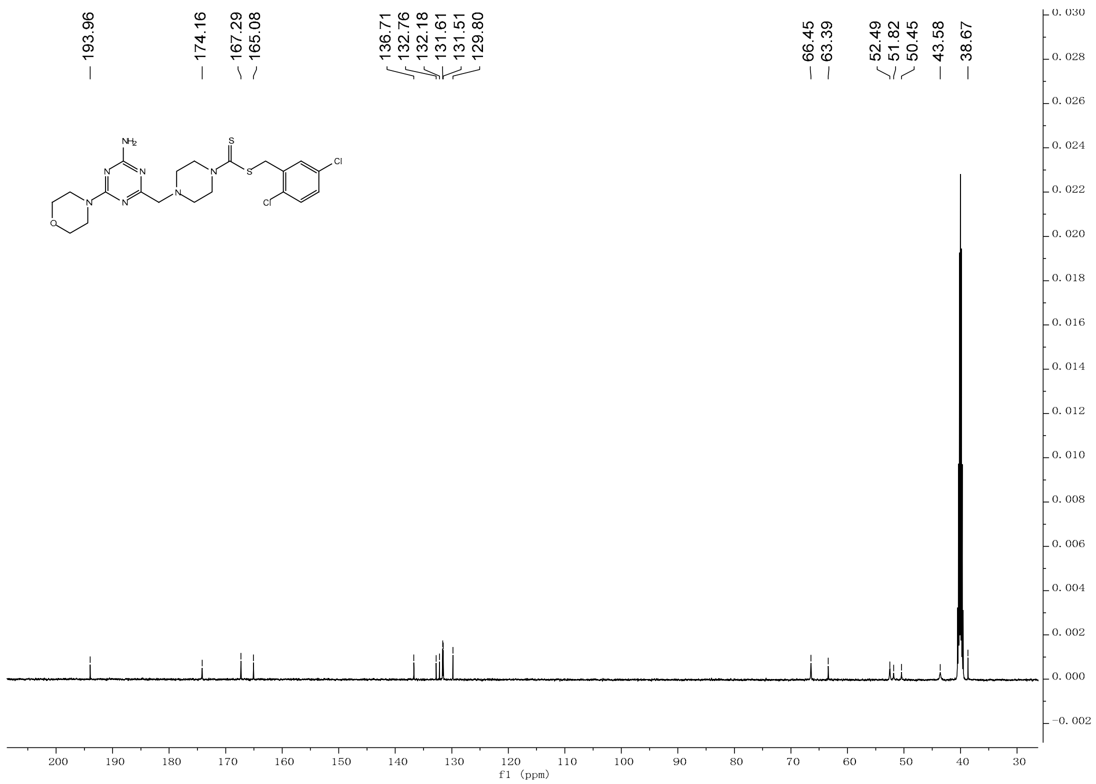

Figure S19. ^{13}C NMR (126 MHz, DMSO-d_6) spectrum of compound **C6**.

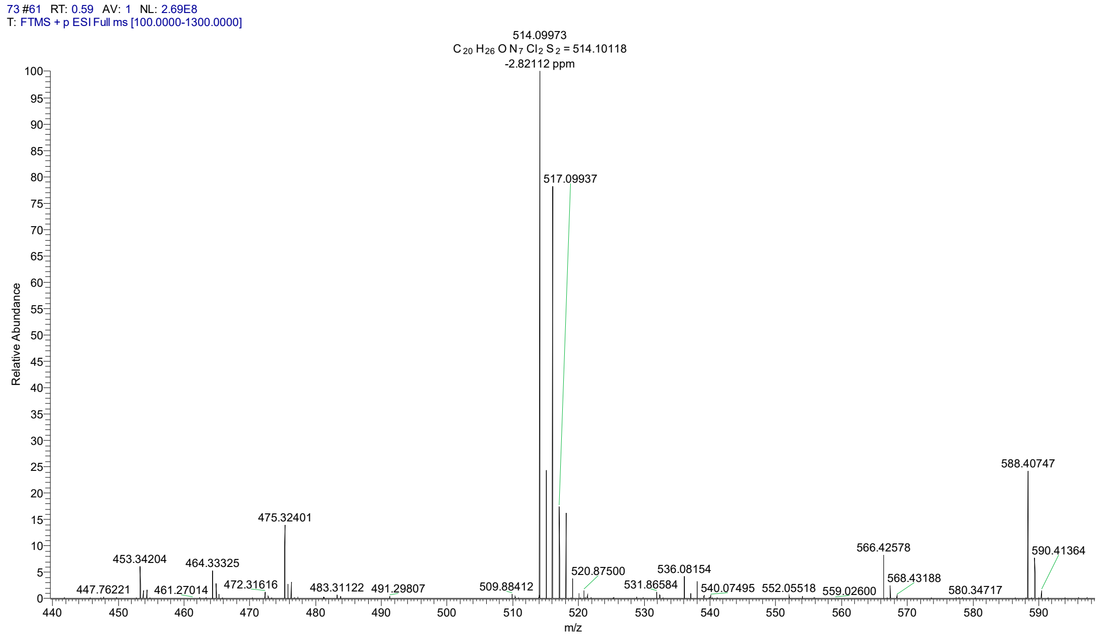

Figure S20. HRMS of compound **C6.**

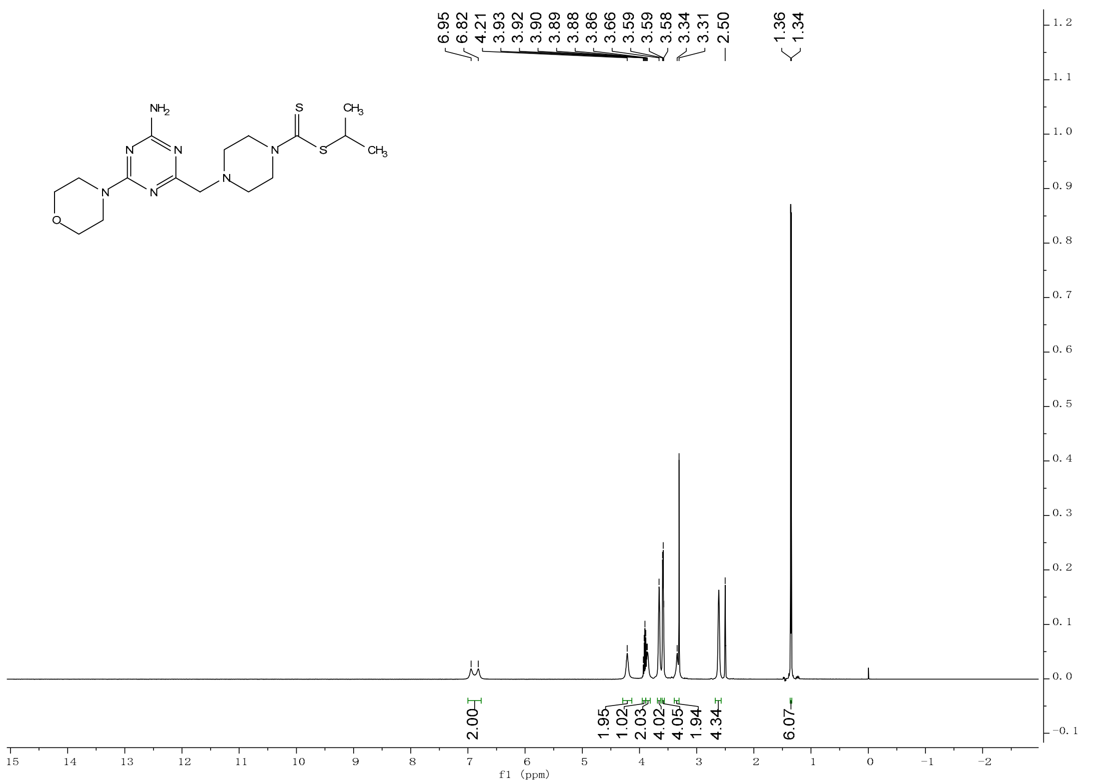

Figure S21. ^{1}H NMR (500 MHz, DMSO-d_6) spectrum of compound **C7**.

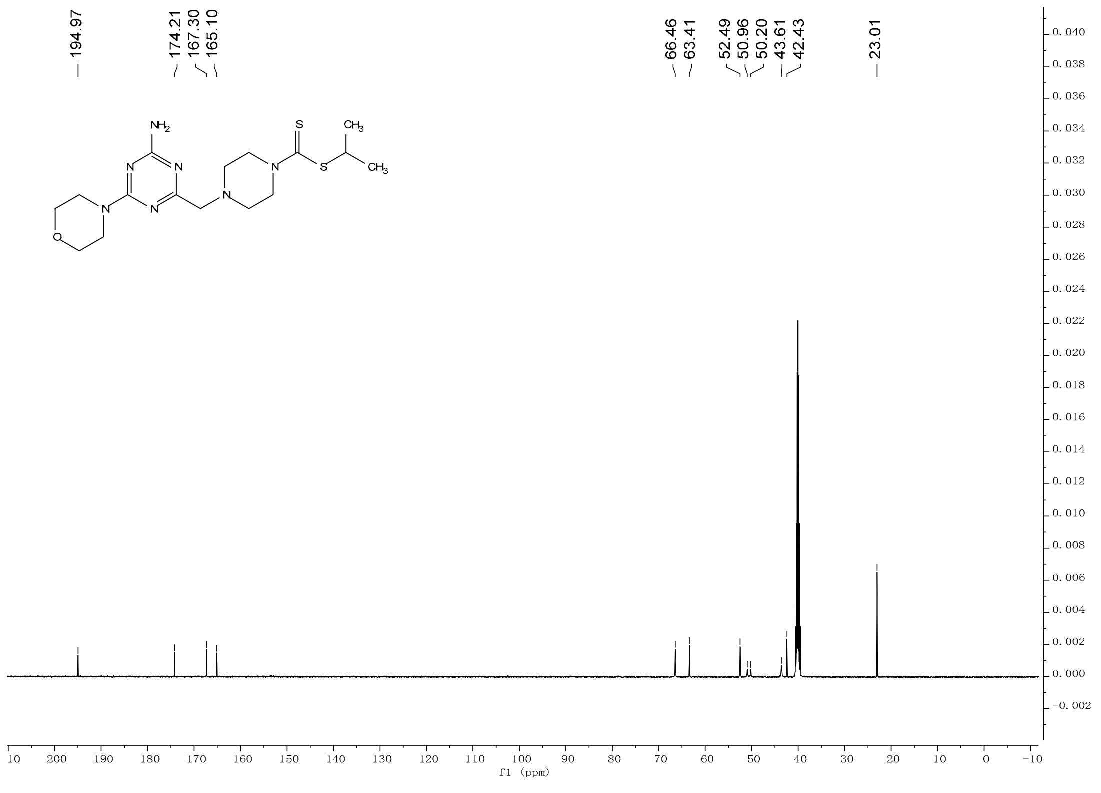

Figure S22. ^{13}C NMR (126 MHz, DMSO-d_6) spectrum of compound **C7**.

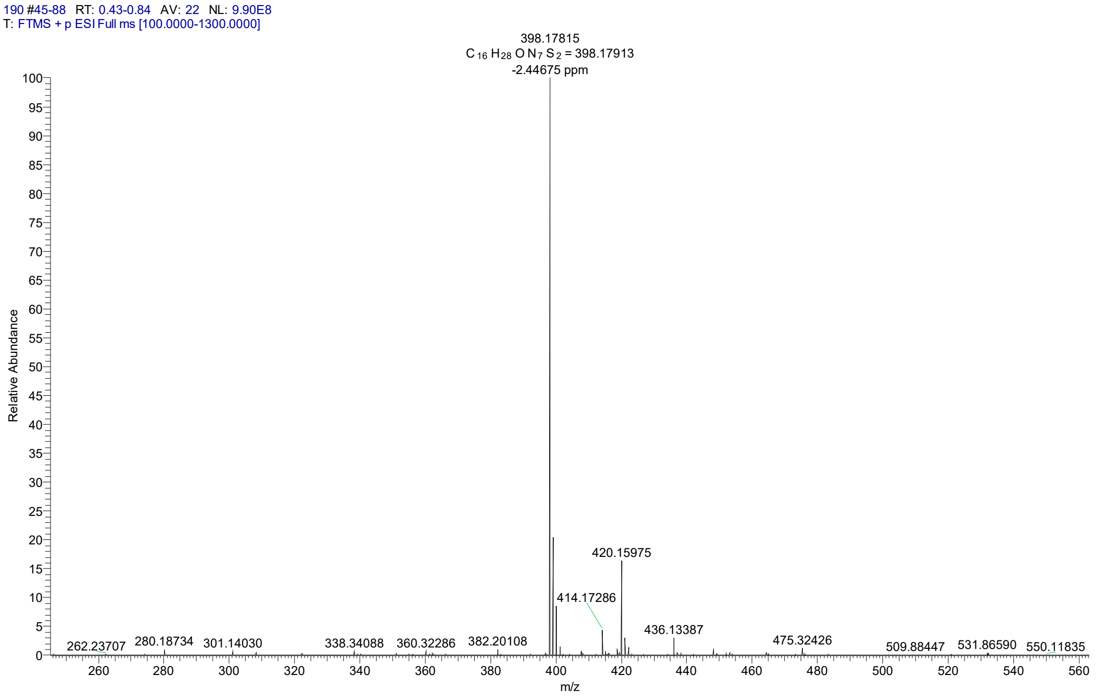

Figure S23. HRMS of compound **C7.**

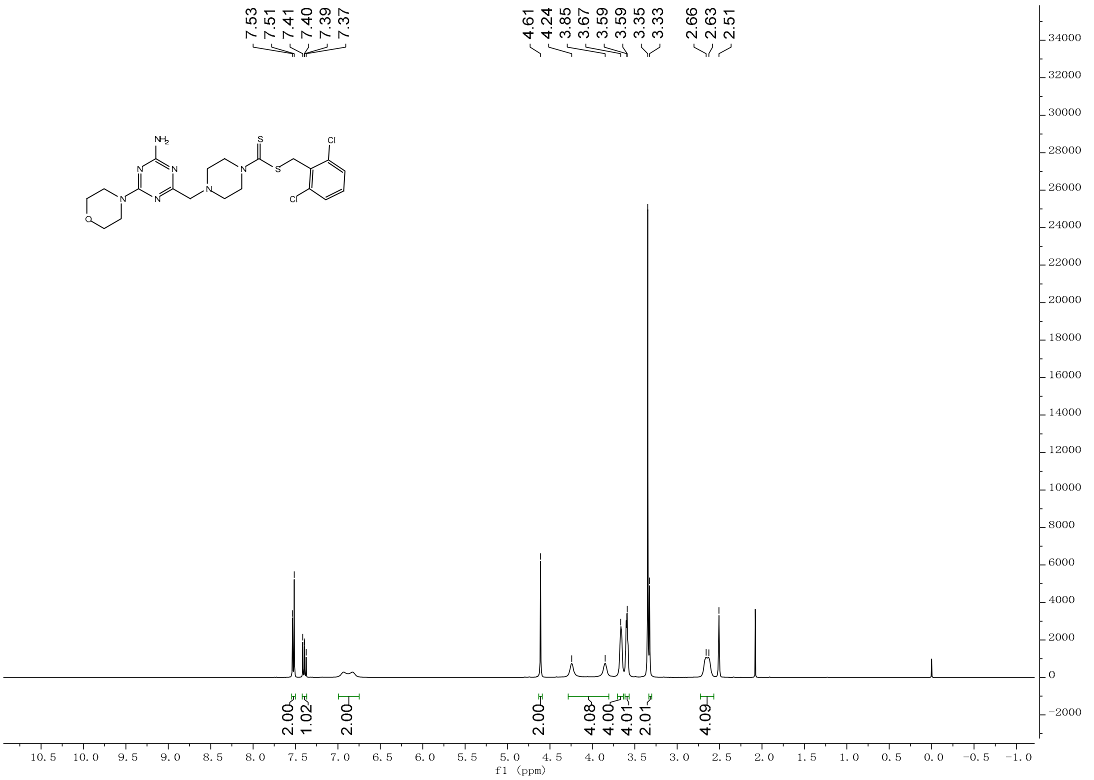

Figure S24. ^{1}H NMR (500 MHz, DMSO-d_6) spectrum of compound **C8**.

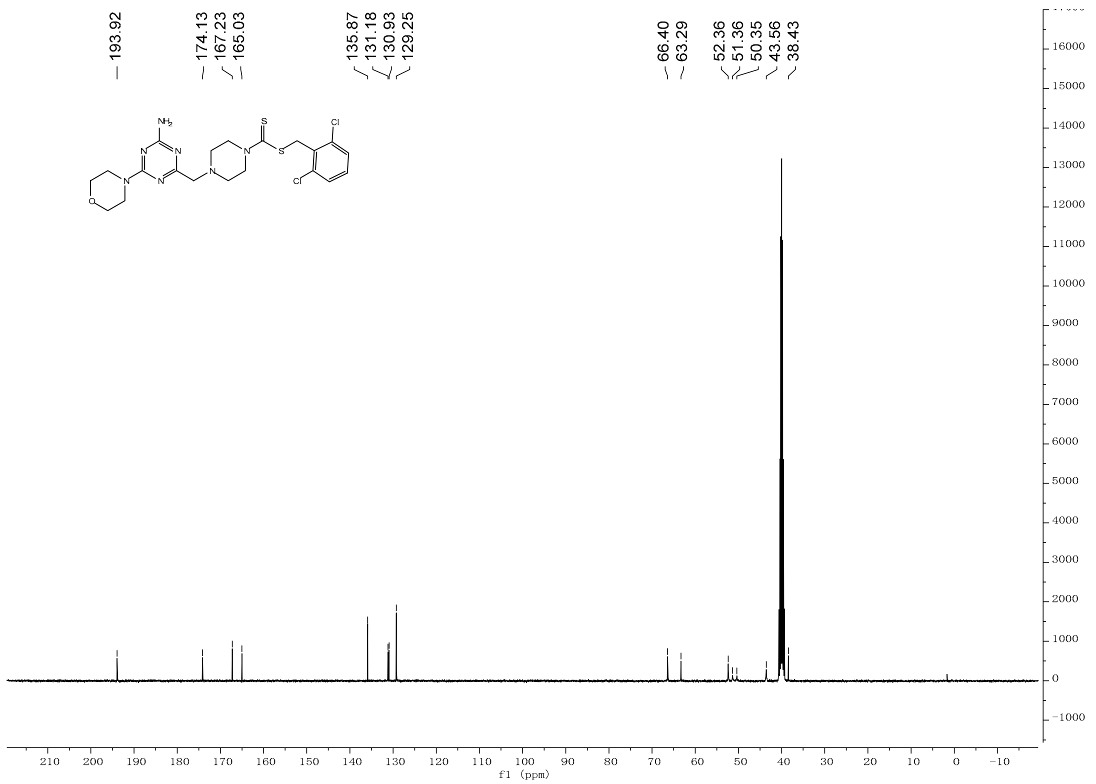

Figure S25. ^{13}C NMR (101 MHz, DMSO-d_6) spectrum of compound **C8**.

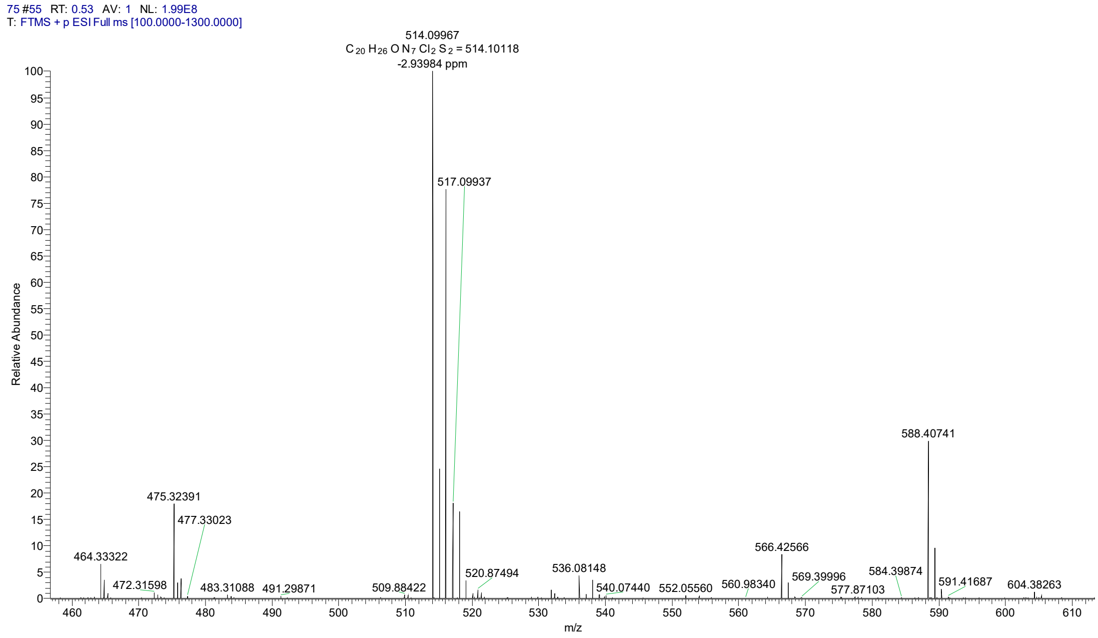

Figure S26. HRMS of compound **C8**.

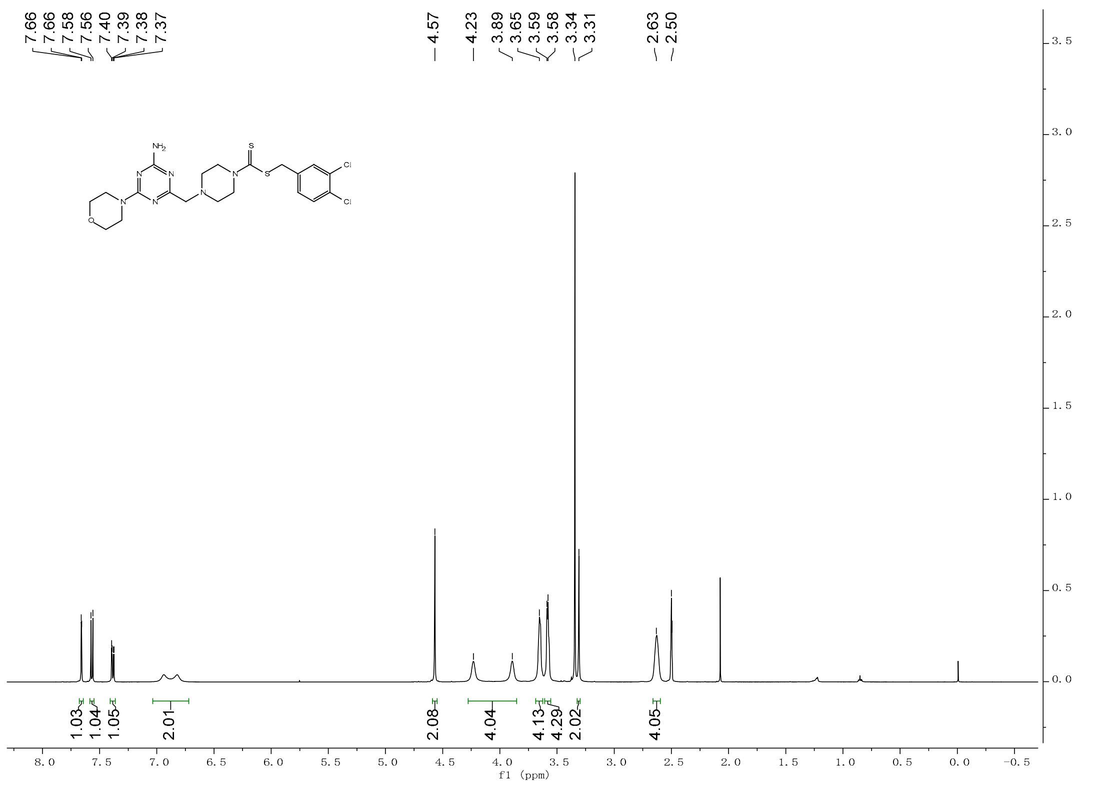

Figure S27. ^{1}H NMR (500 MHz, DMSO-d_6) spectrum of compound **C9**.

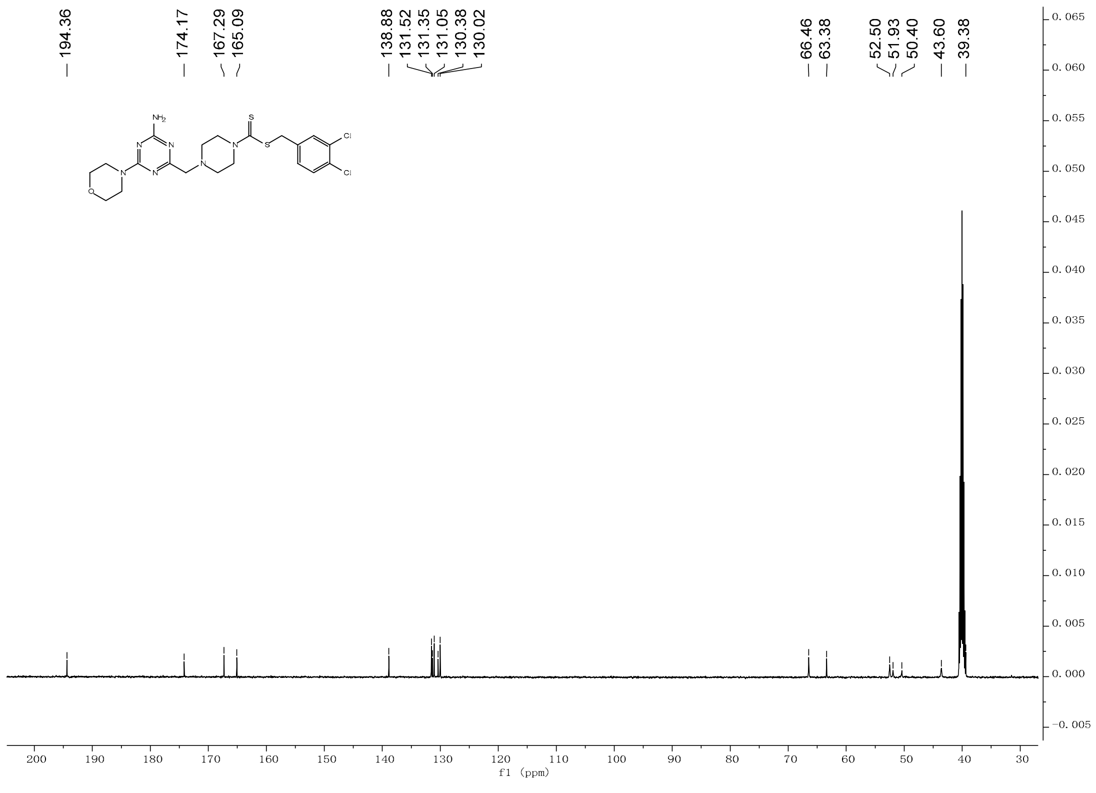

Figure S28. ^{13}C NMR (126 MHz, DMSO-d_6) spectrum of compound **C9**.

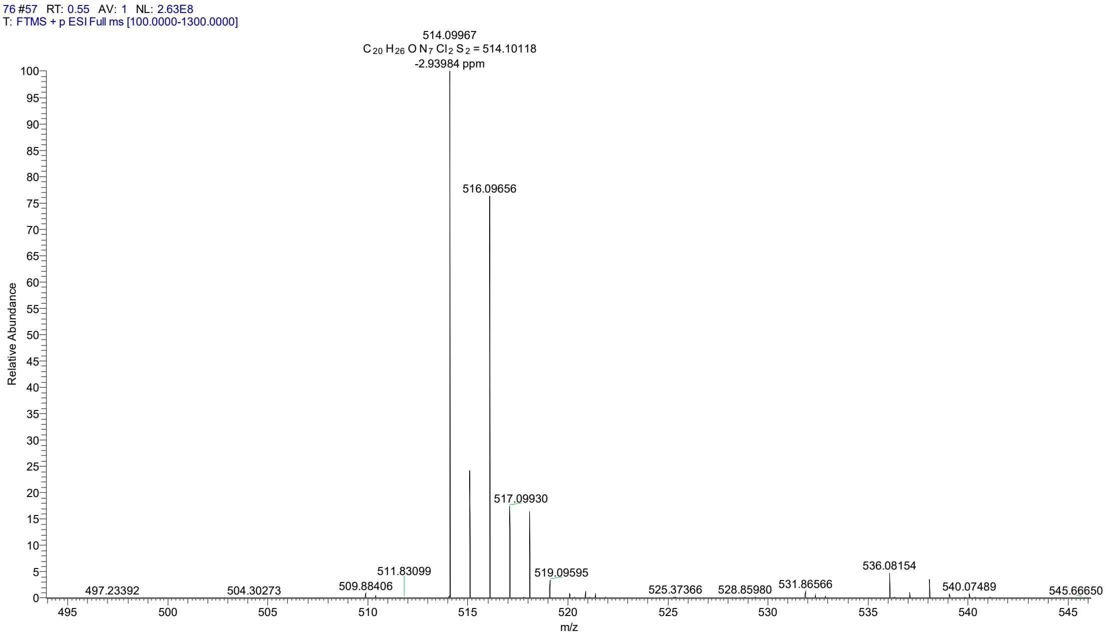

Figure S29. HRMS of compound **C9**.

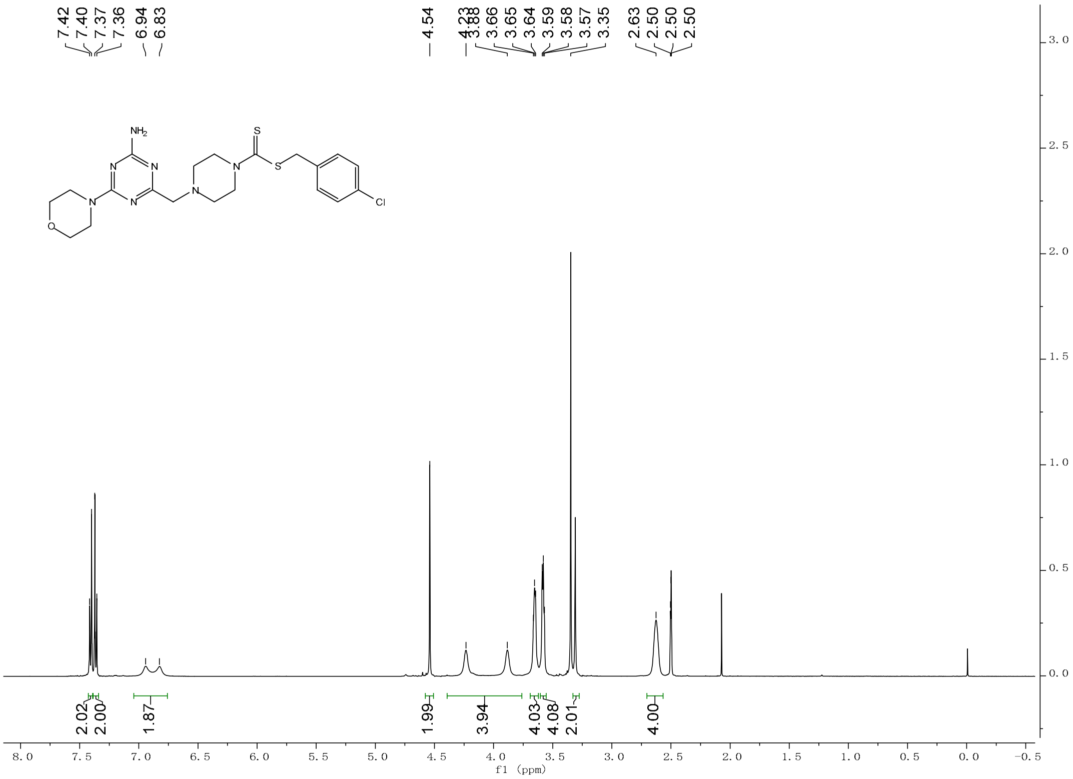

Figure S30. ^{1}H NMR (500 MHz, DMSO-d_6) spectrum of compound **C10**.

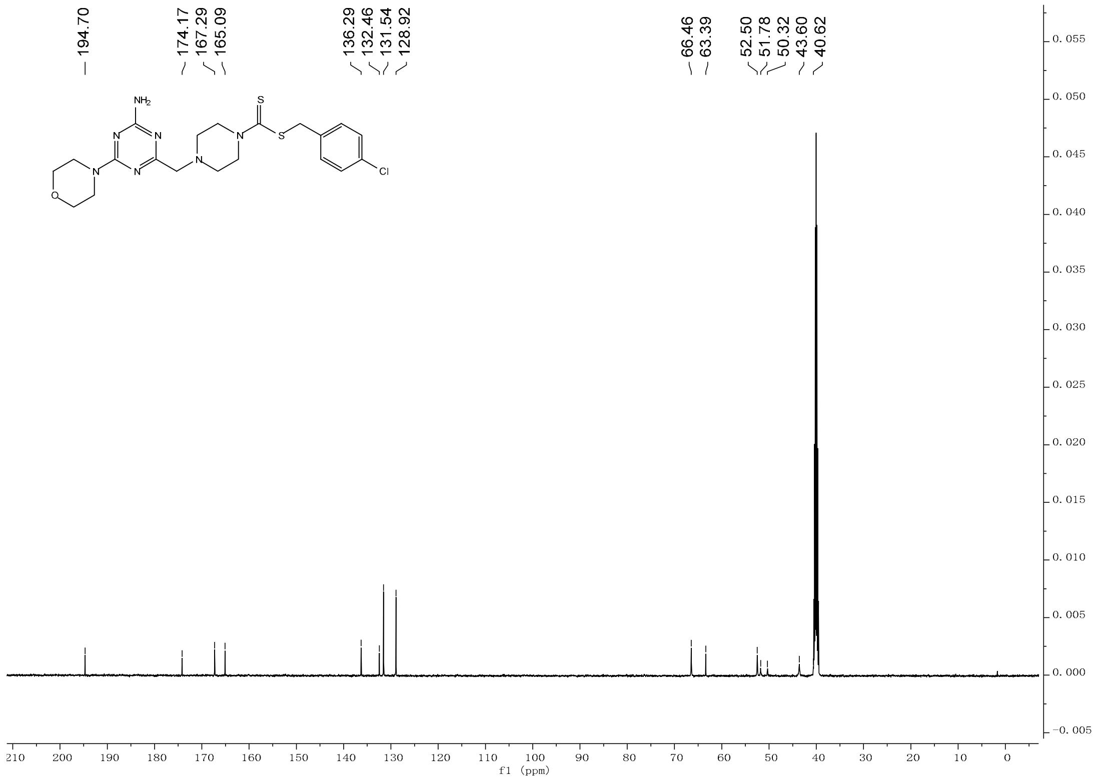

Figure S31. ^{13}C NMR (126 MHz, DMSO-d_6) spectrum of compound **C10**.

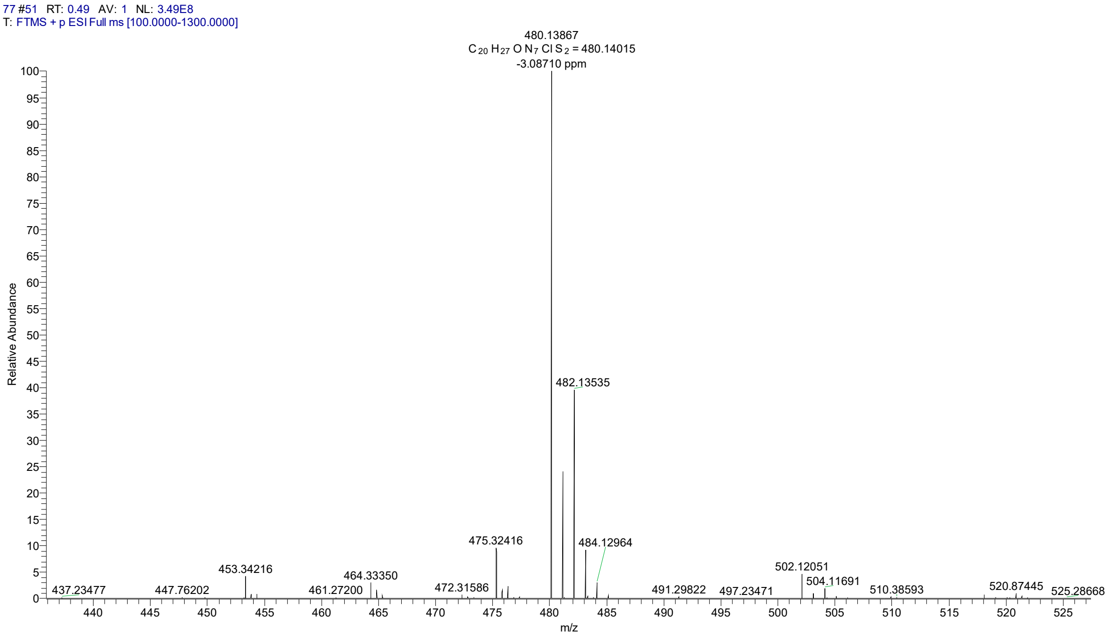

Figure S32. HRMS of compound **C10**.

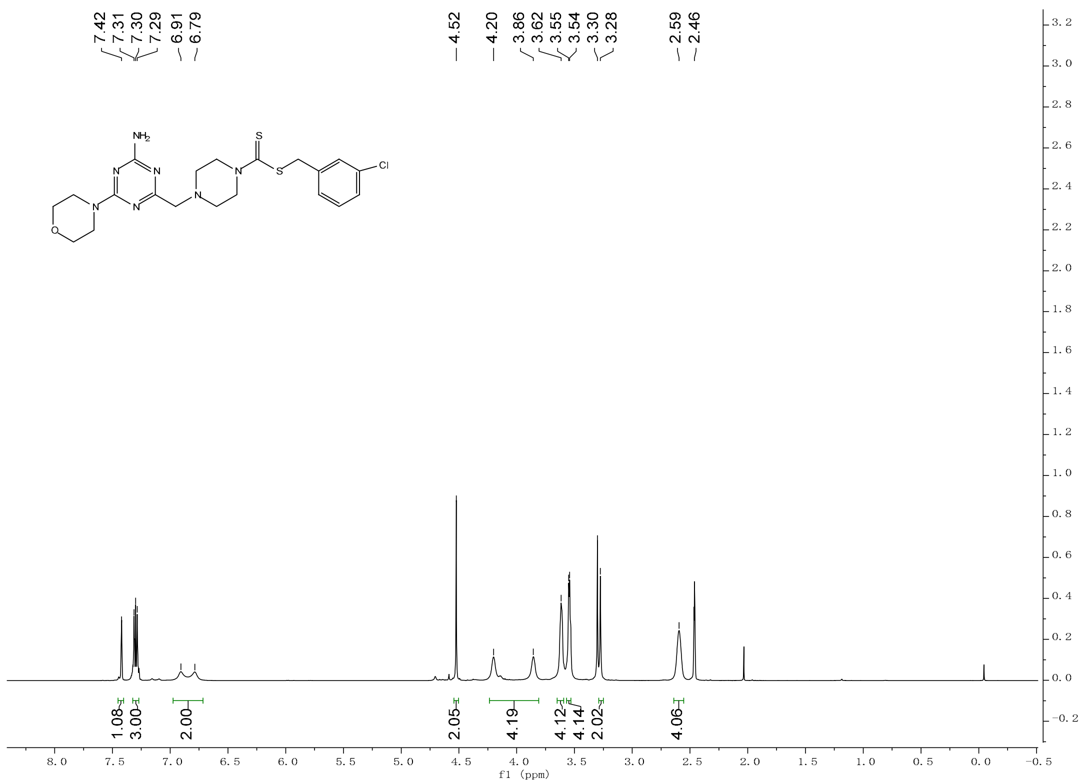

Figure S33. ^{1}H NMR (500 MHz, DMSO-d_6) spectrum of compound **C11**.

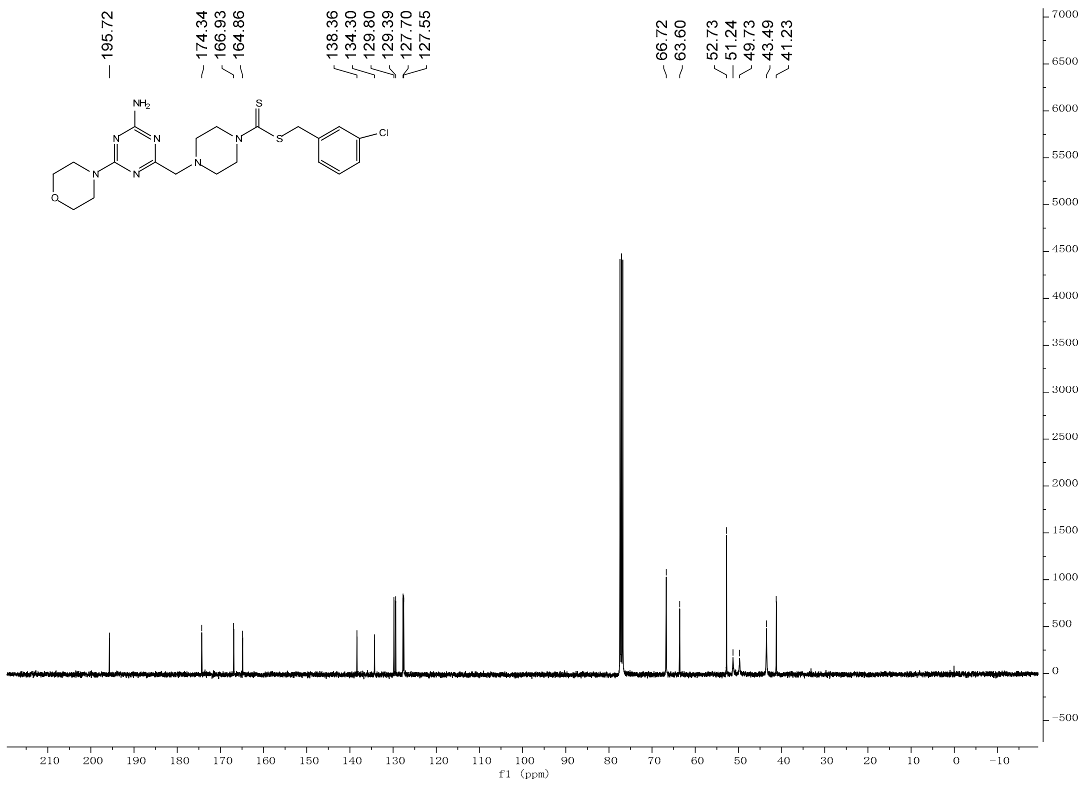

Figure S34. ^{13}C NMR (101 MHz, DMSO-d_6) spectrum of compound **C11**.

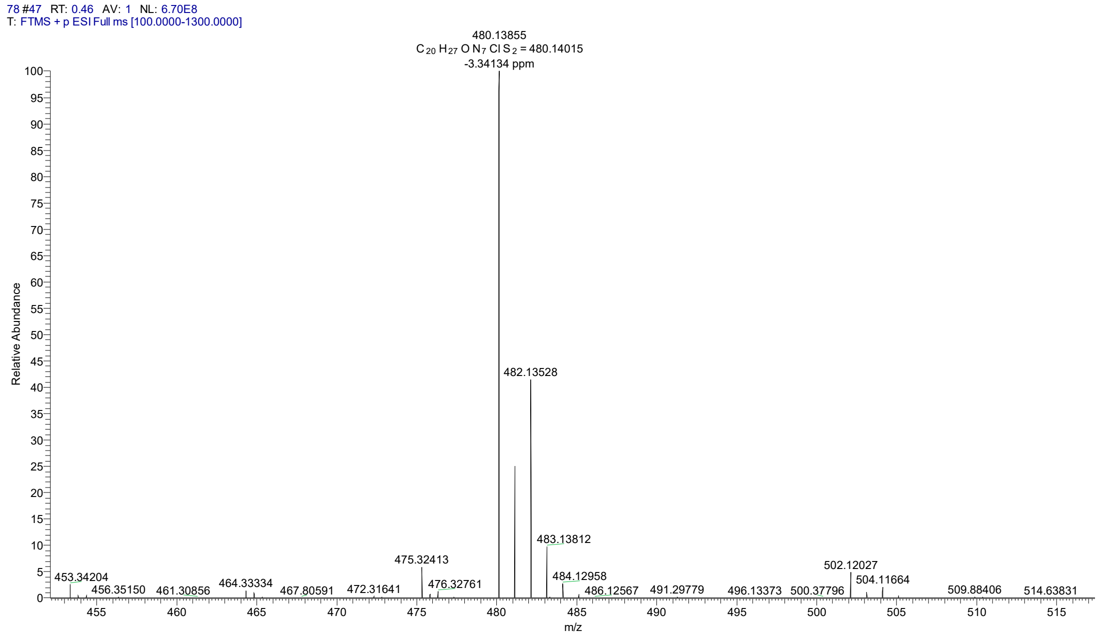

Figure S35. HRMS of compound **C11**.

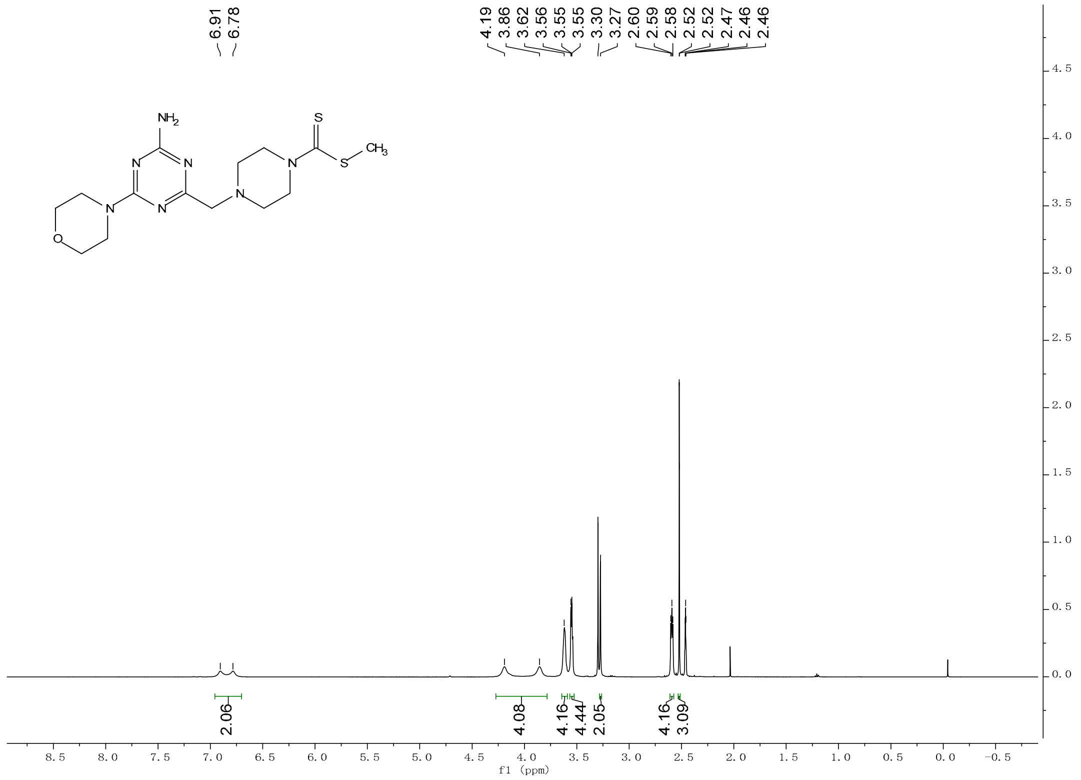

Figure S36. ^{1}H NMR (500 MHz, DMSO-d_6) spectrum of compound **C12**.

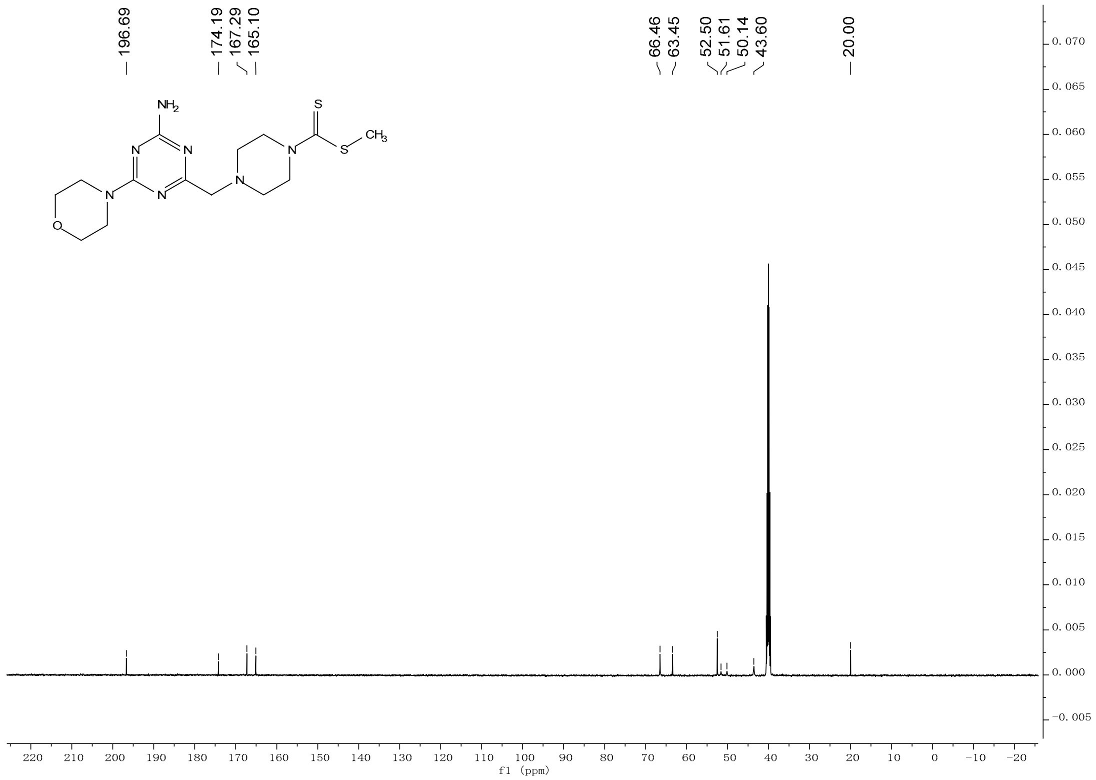

Figure S37. ^{13}C NMR (126 MHz, DMSO-d_6) spectrum of compound **C12**.

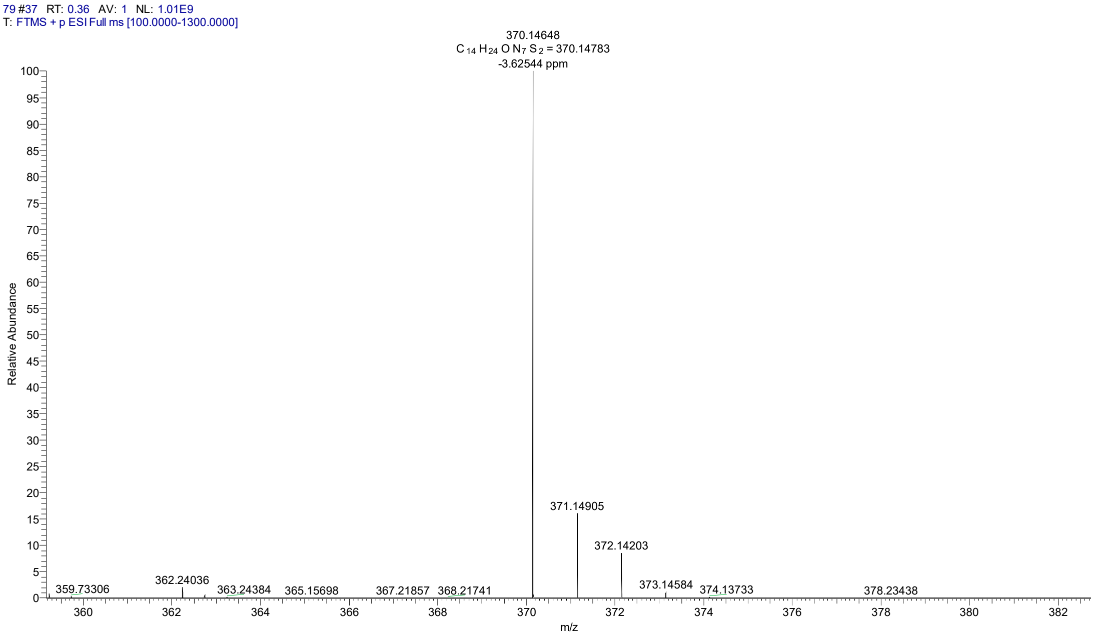

Figure S38. HRMS of compound **C12**.

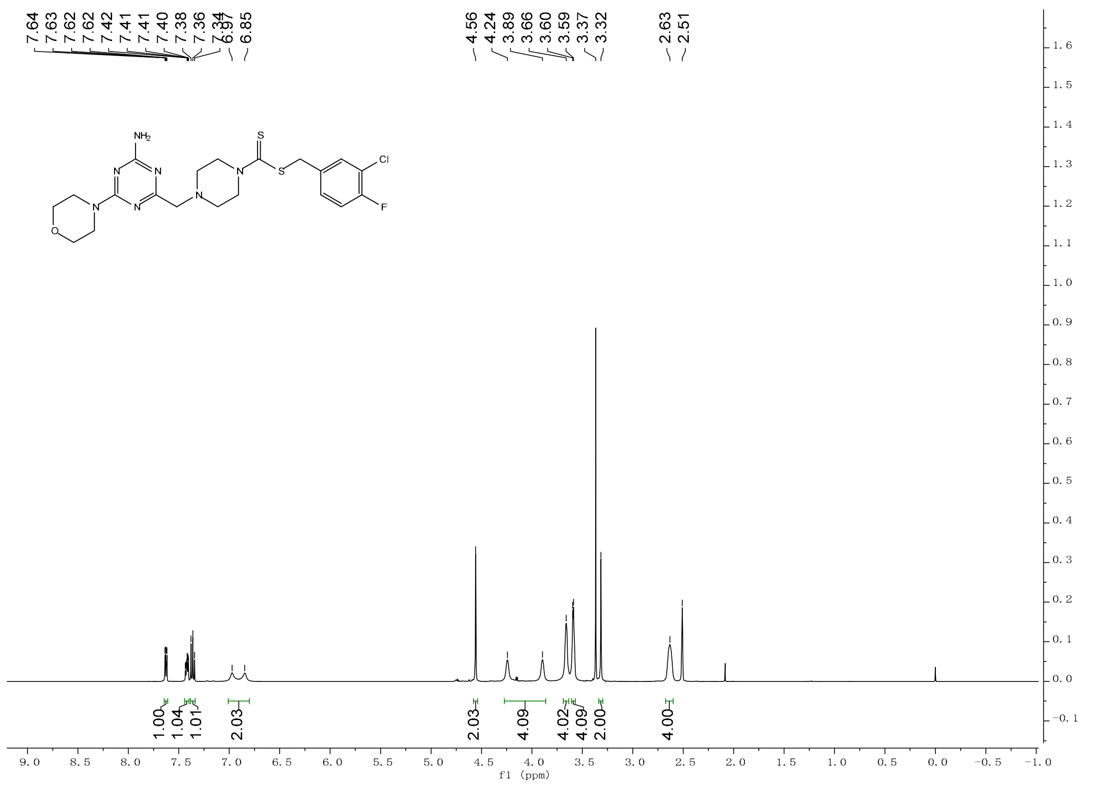

Figure S39. ^{1}H NMR (500 MHz, DMSO-d_6) spectrum of compound **C13**.

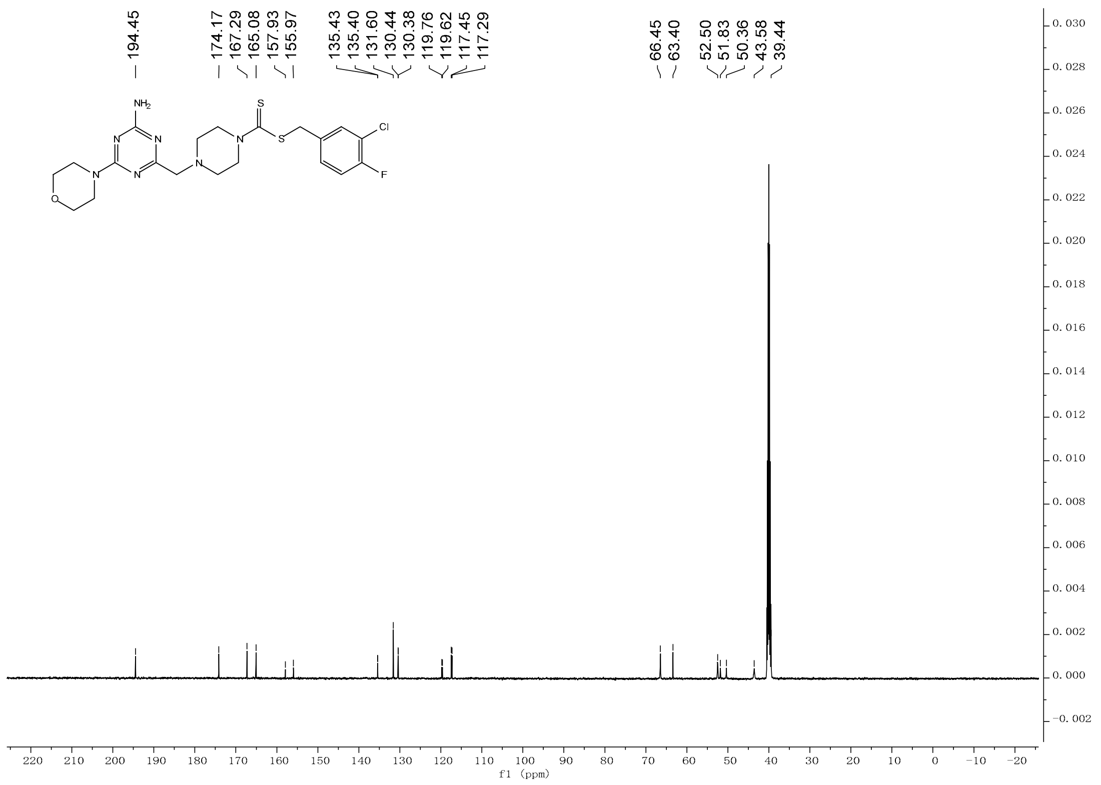

Figure S40. ^{13}C NMR (126 MHz, DMSO-d_6) spectrum of compound **C13**.

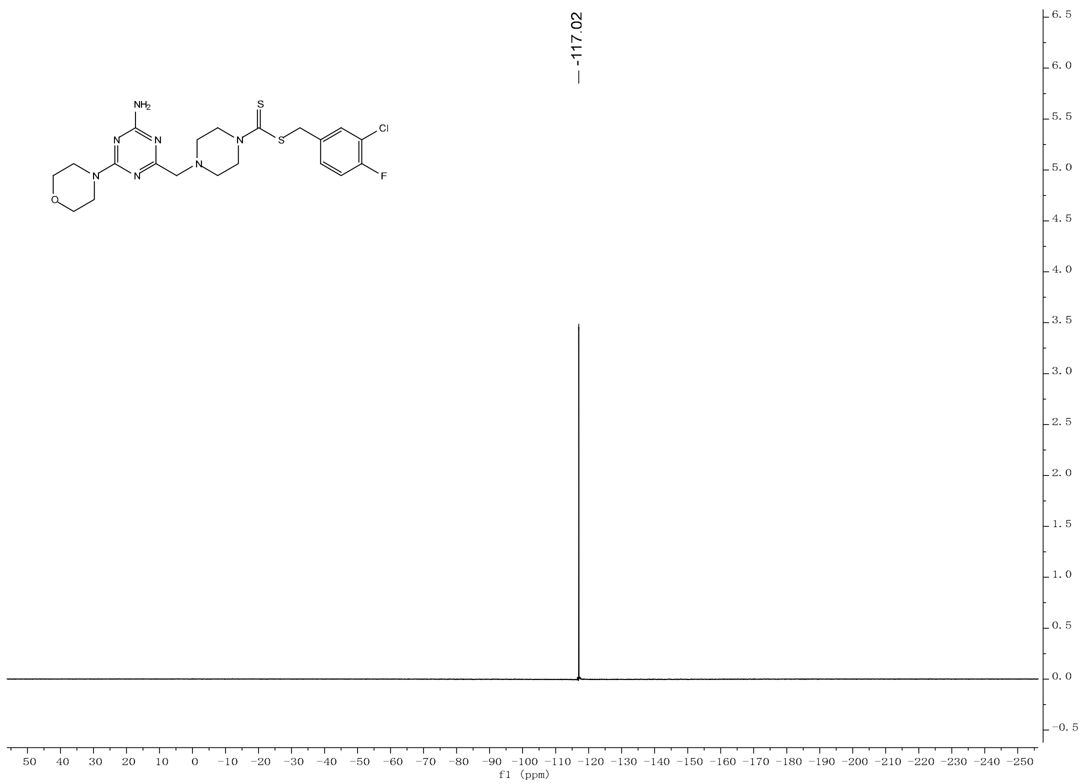

Figure S41. ^{19}F NMR (471 MHz, $CDCl_3$) spectrum of compound **C13**.

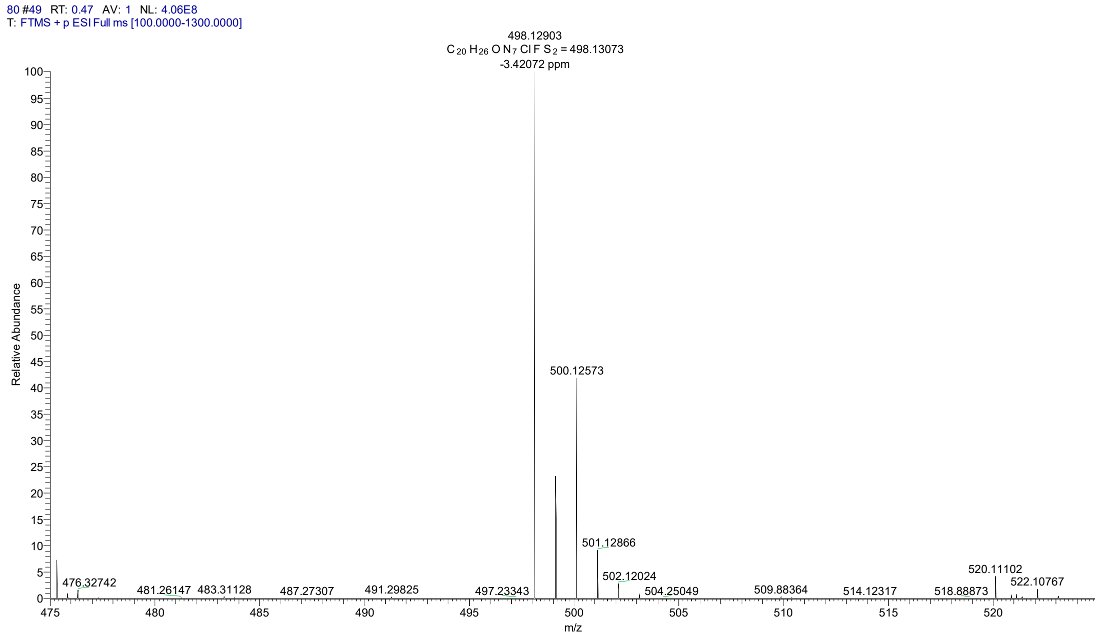

Figure S42. HRMS of compound **C13**.

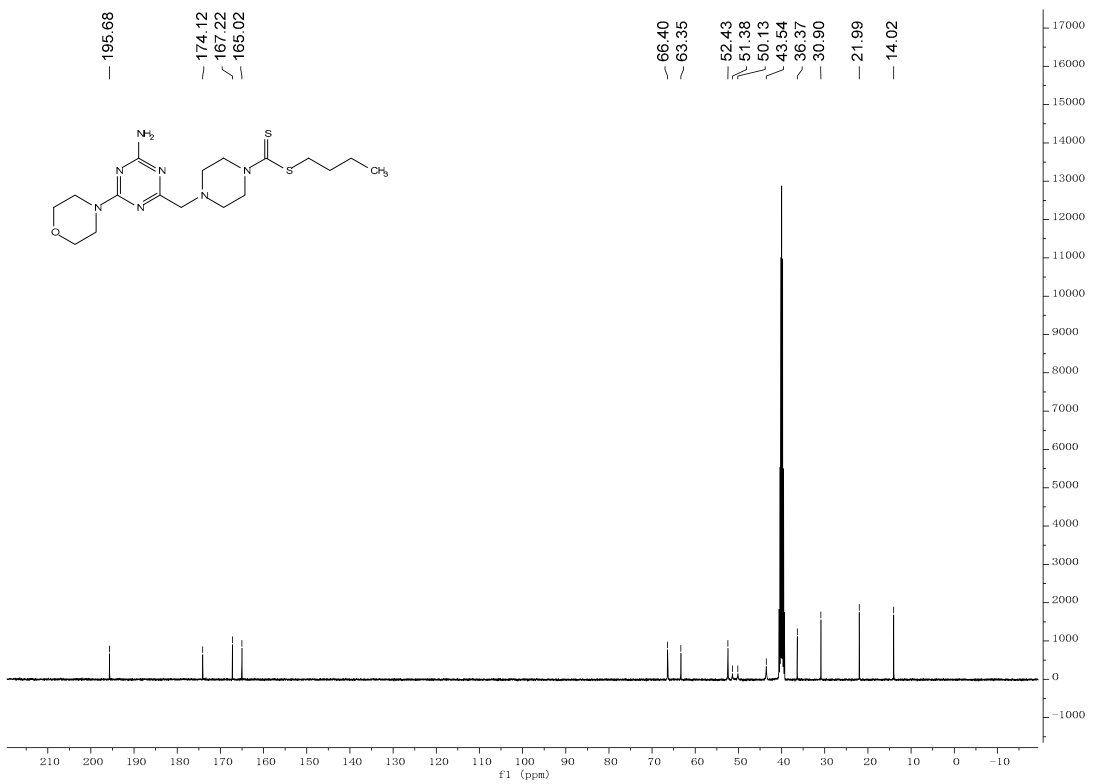

Figure S43. ^{1}H NMR (500 MHz, DMSO-d_6) spectrum of compound **C14**.

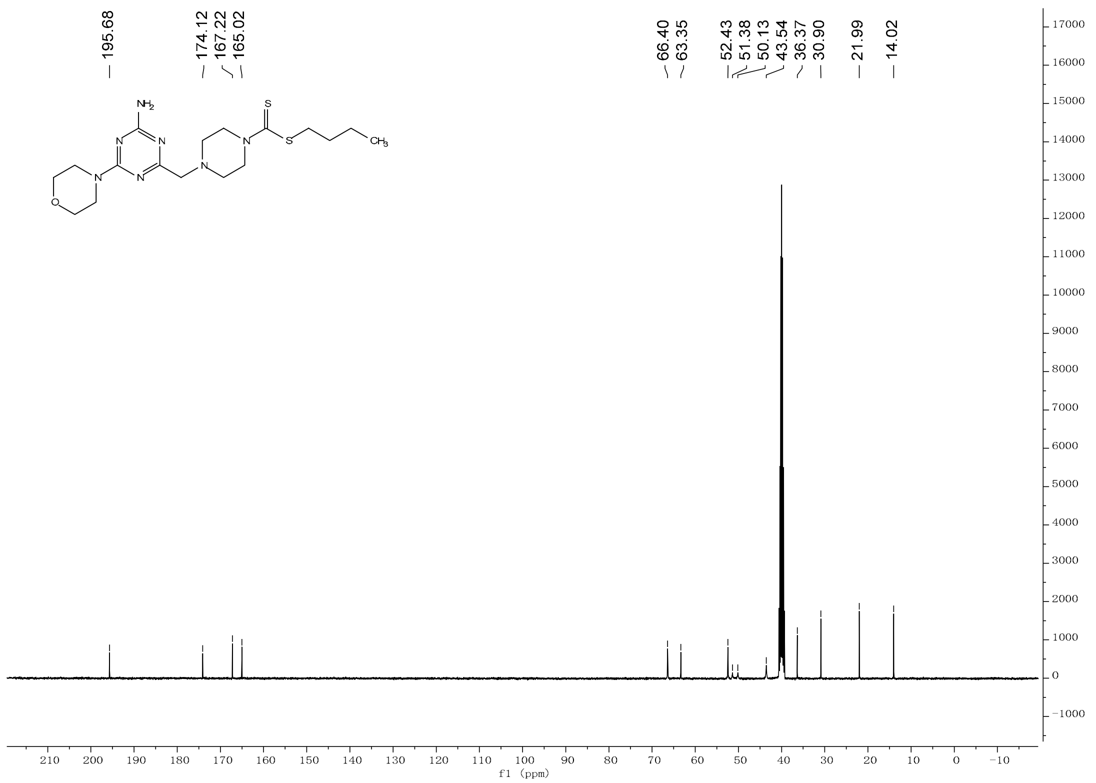

Figure S44. ^{13}C NMR (101 MHz, DMSO-d_6) spectrum of compound **C14**.

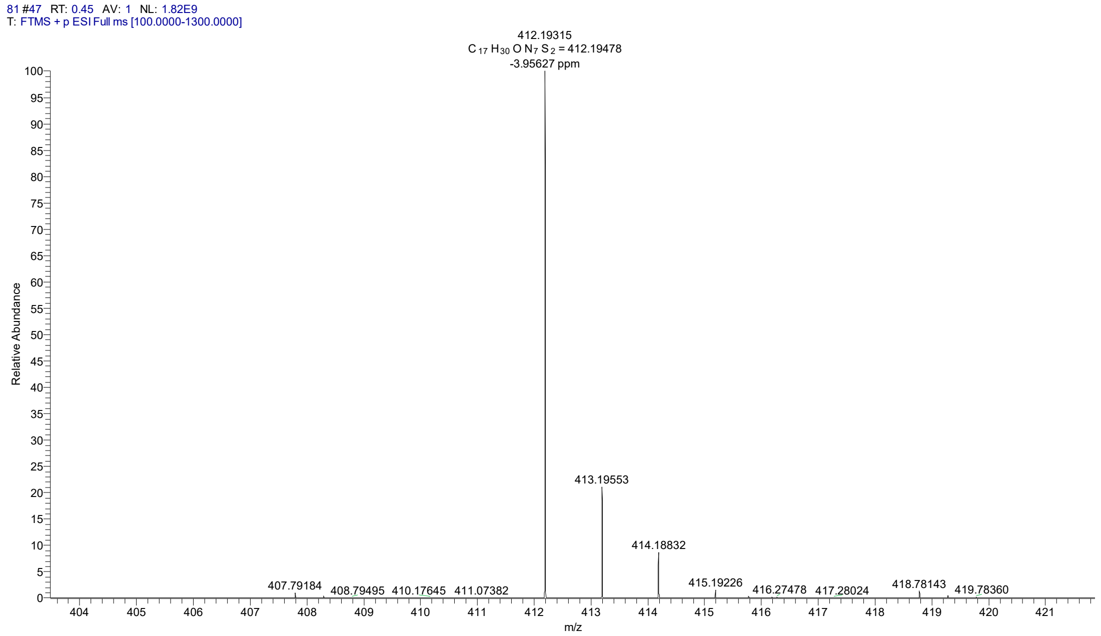

Figure S45. HRMS of compound **C14**.

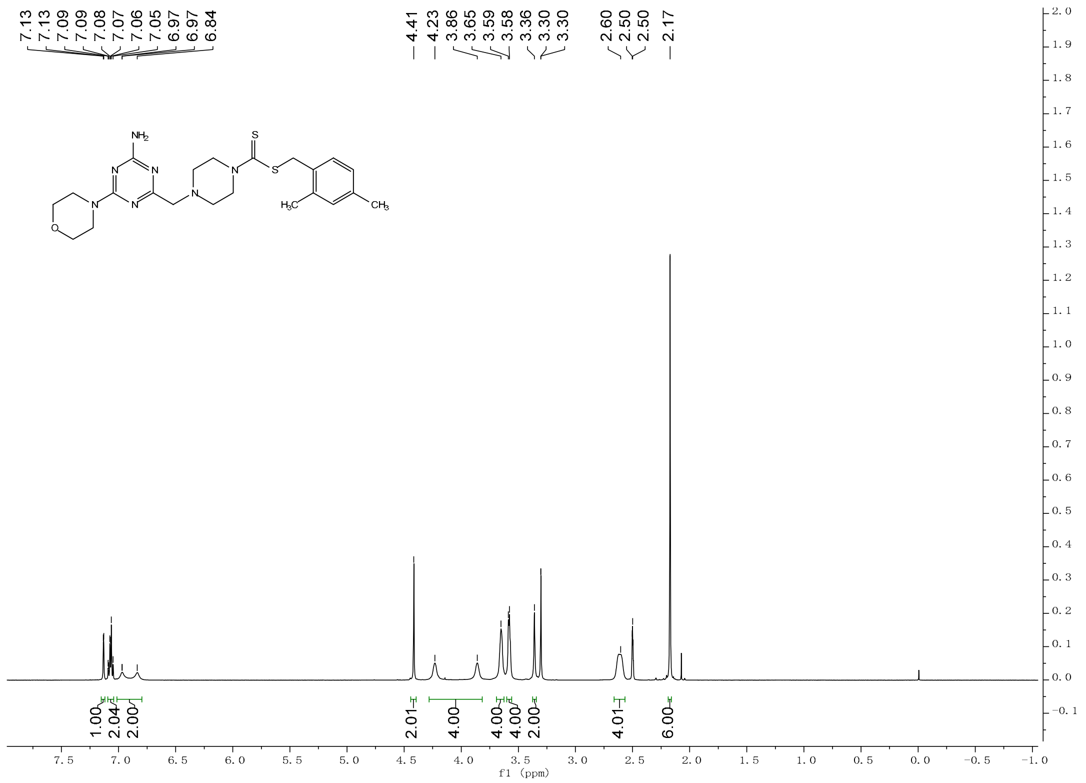

Figure S46. ^{1}H NMR (500 MHz, DMSO-d_6) spectrum of compound **C15**.

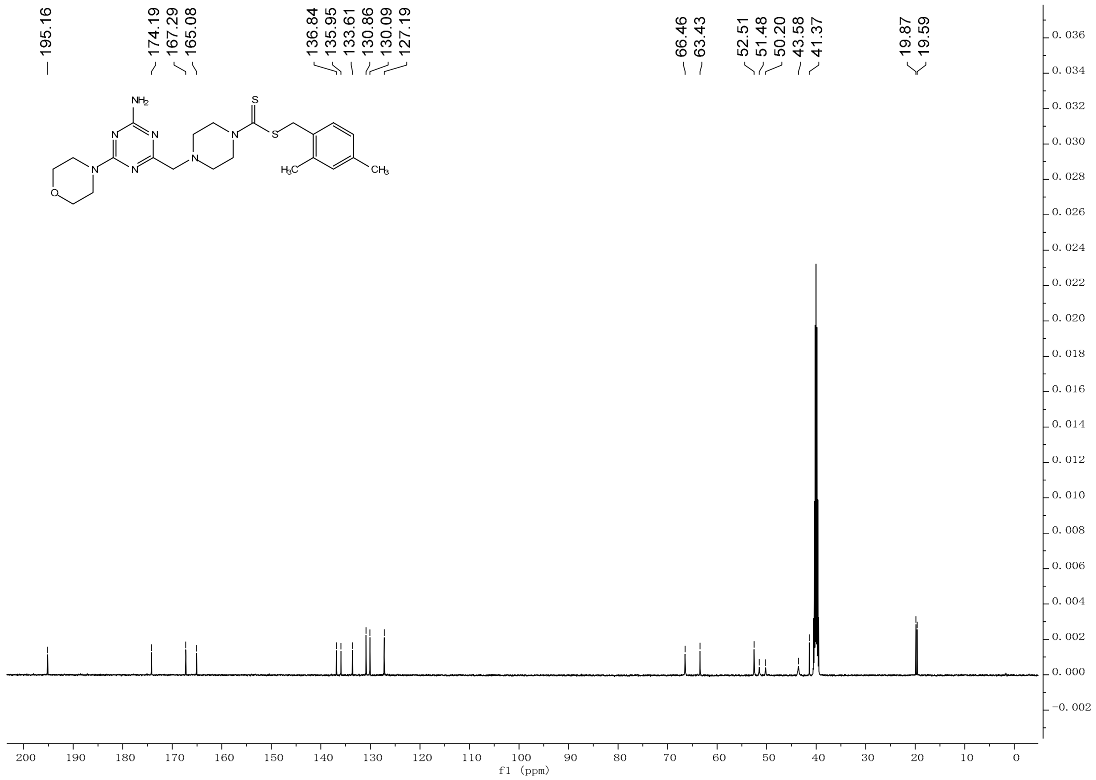

Figure S47. ^{13}C NMR (126 MHz, DMSO-d_6) spectrum of compound **C15**.

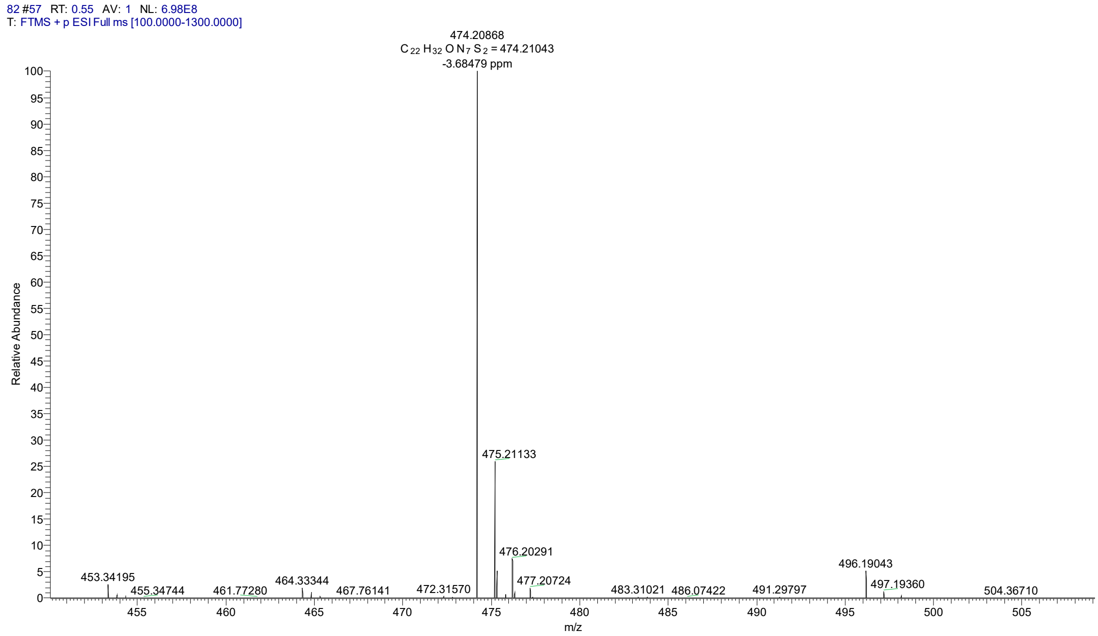

Figure S48. HRMS of compound **C15**.

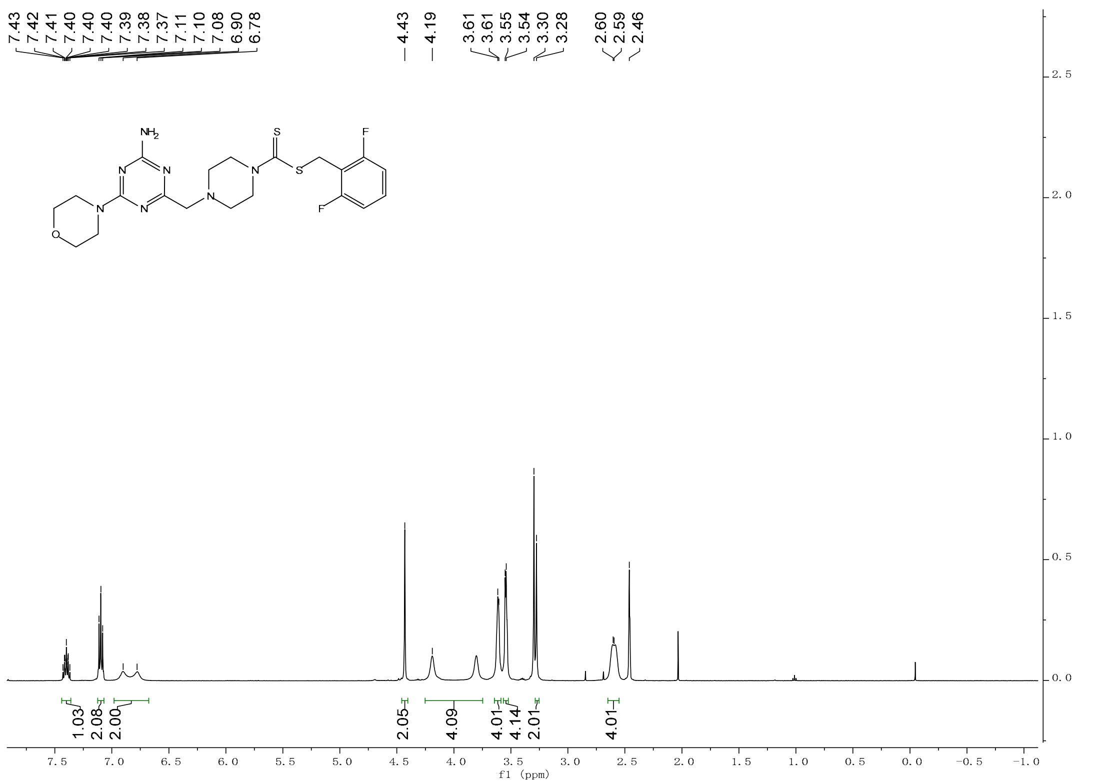

Figure S49. ^{1}H NMR (500 MHz, DMSO-d_6) spectrum of compound **C16**.

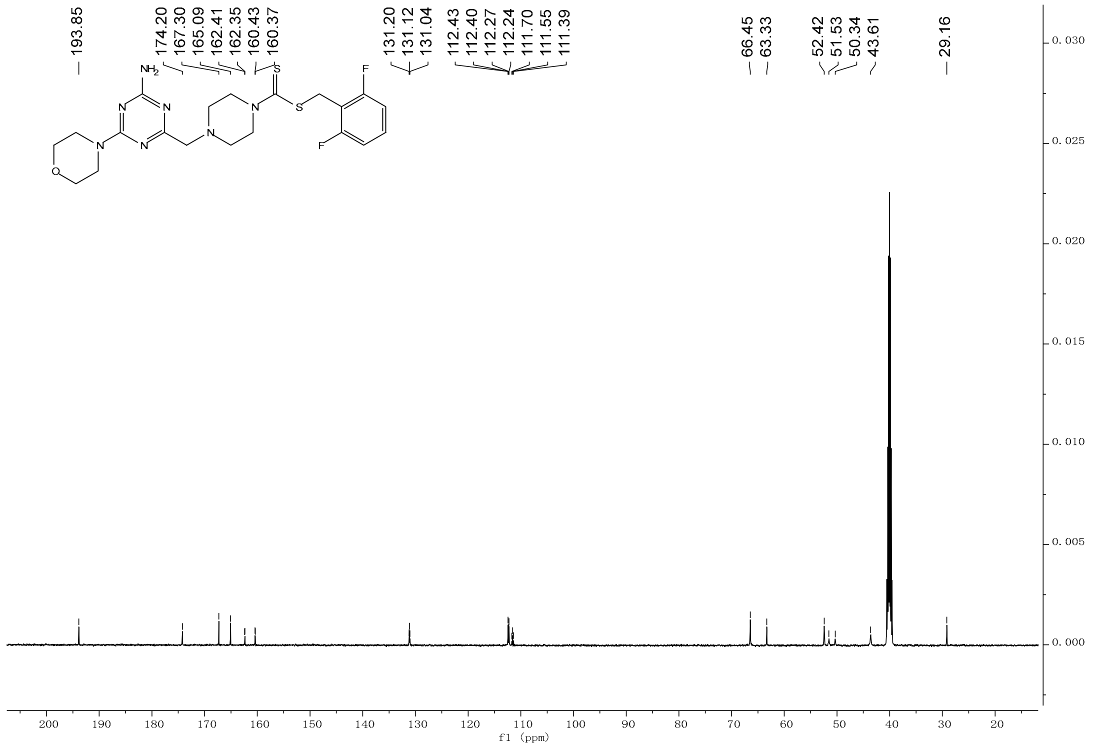

Figure S50. ^{13}C NMR (126 MHz, DMSO-d_6) spectrum of compound **C16**.

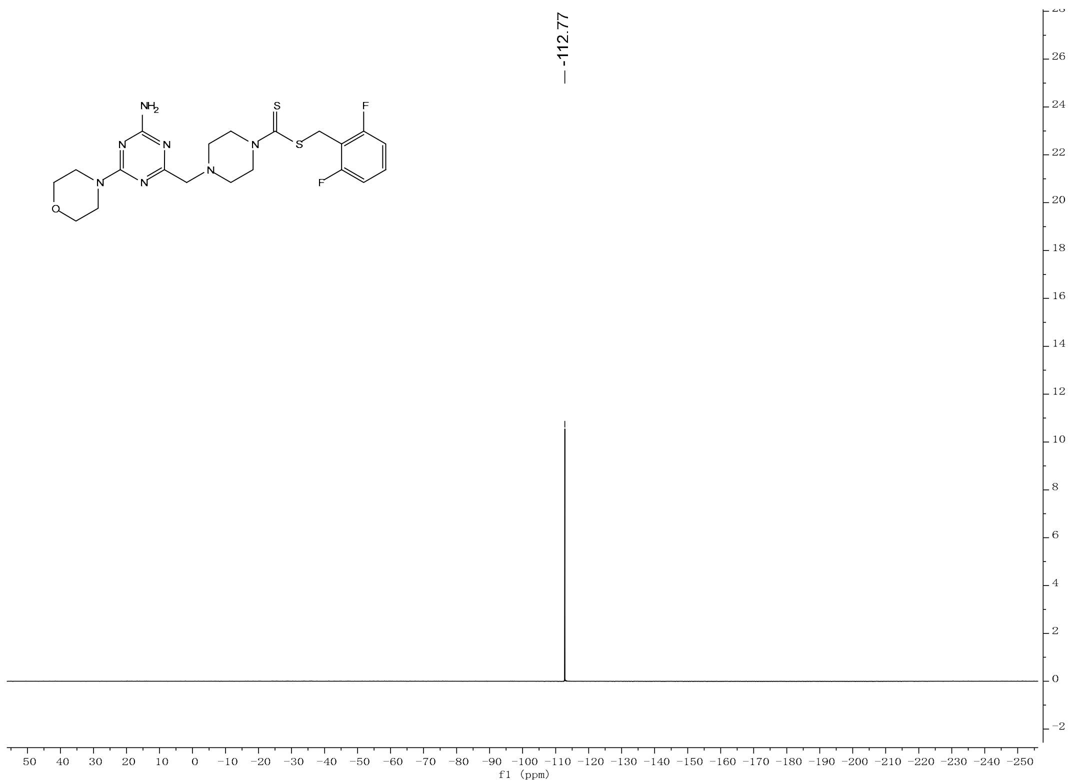

Figure S51. ^{19}F NMR (471 MHz, $CDCl_3$) spectrum of compound **C16**.

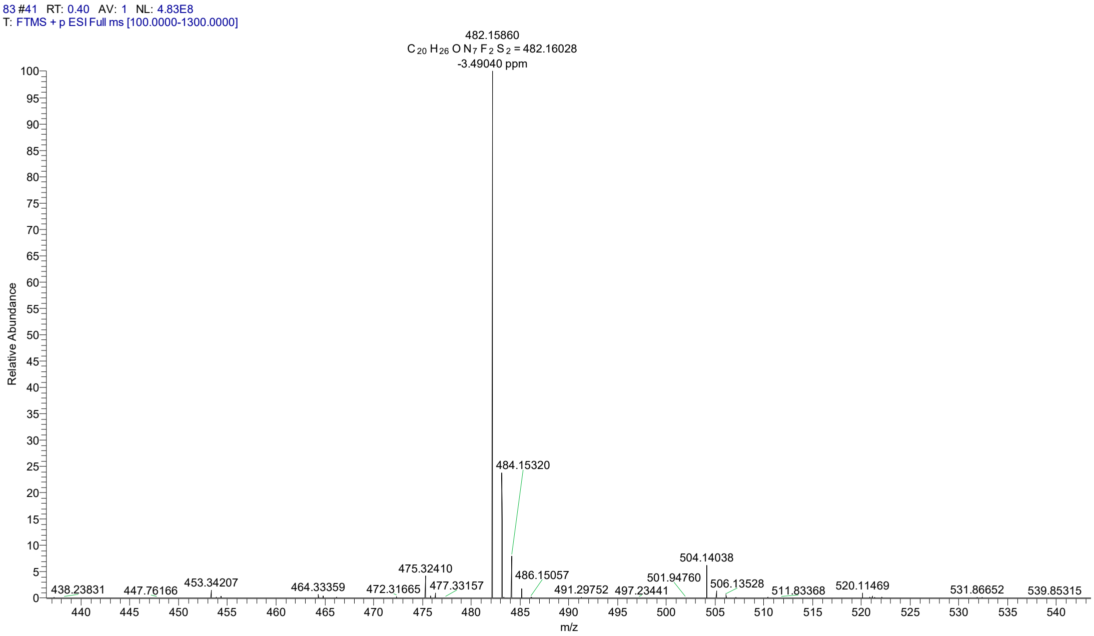

Figure S52. HRMS of compound **C16**.

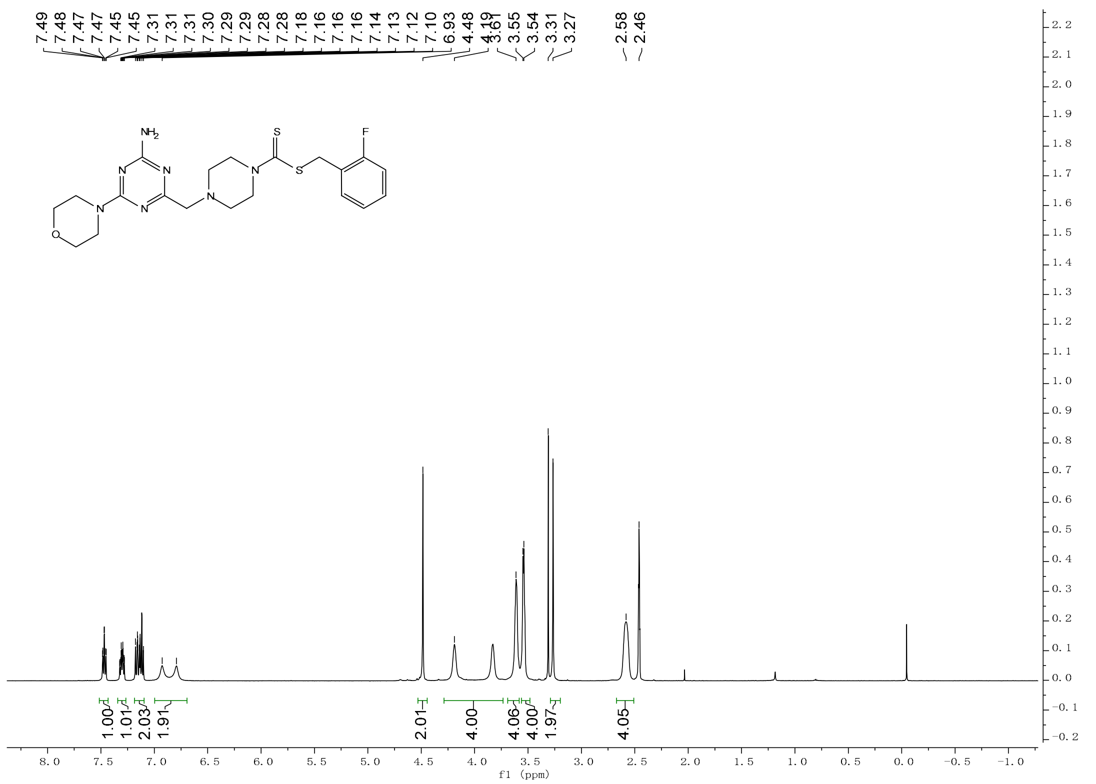

Figure S53. ^{1}H NMR (500 MHz, DMSO-d_6) spectrum of compound **C17**.

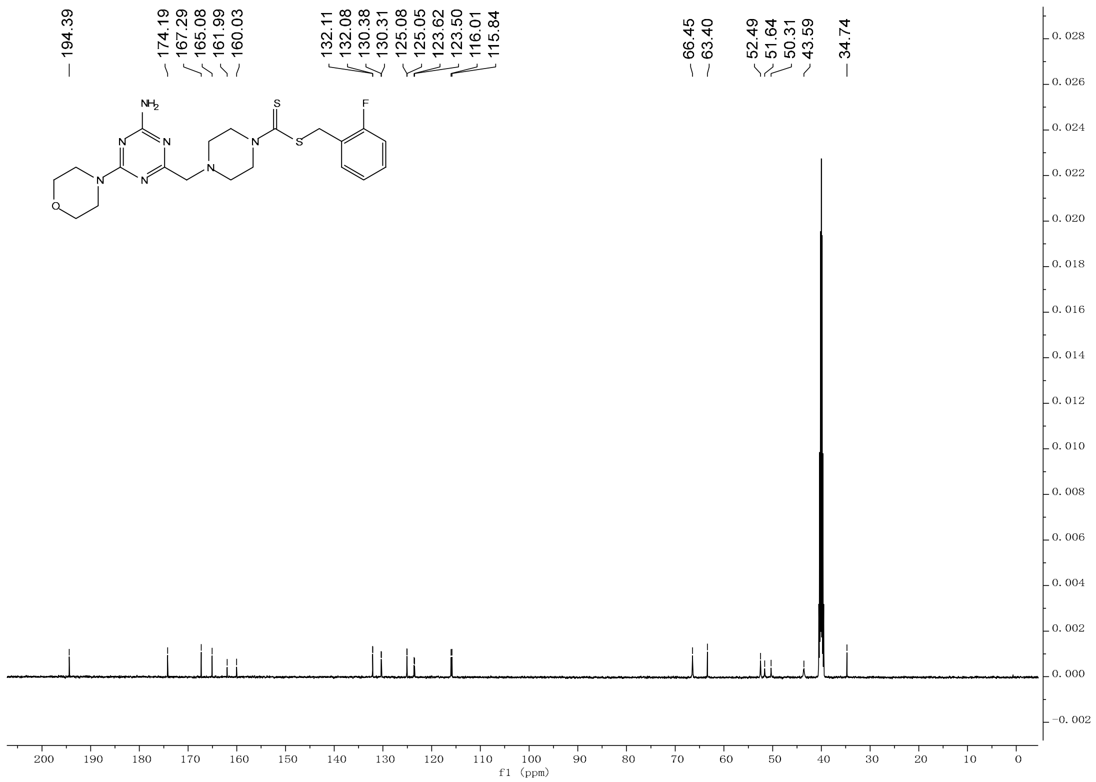

Figure S54. ^{13}C NMR (126 MHz, DMSO-d_6) spectrum of compound **C17**.

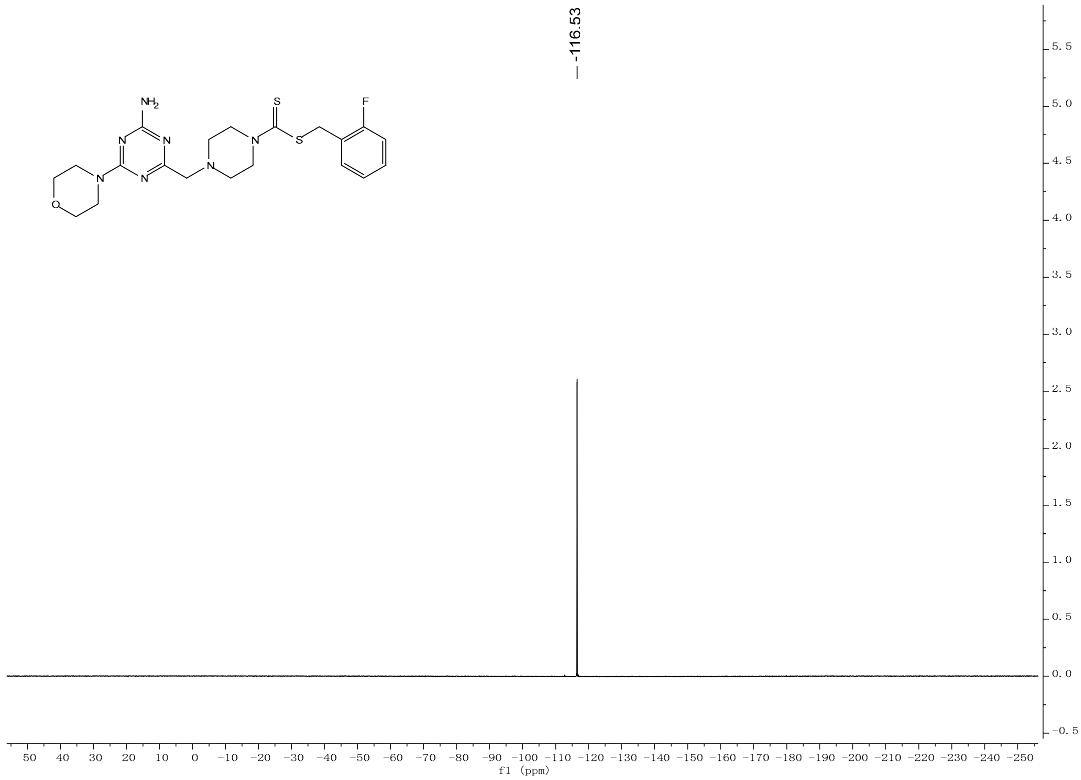

Figure S55. ^{19}F NMR (471 MHz, $CDCl_3$) spectrum of compound **C17**.

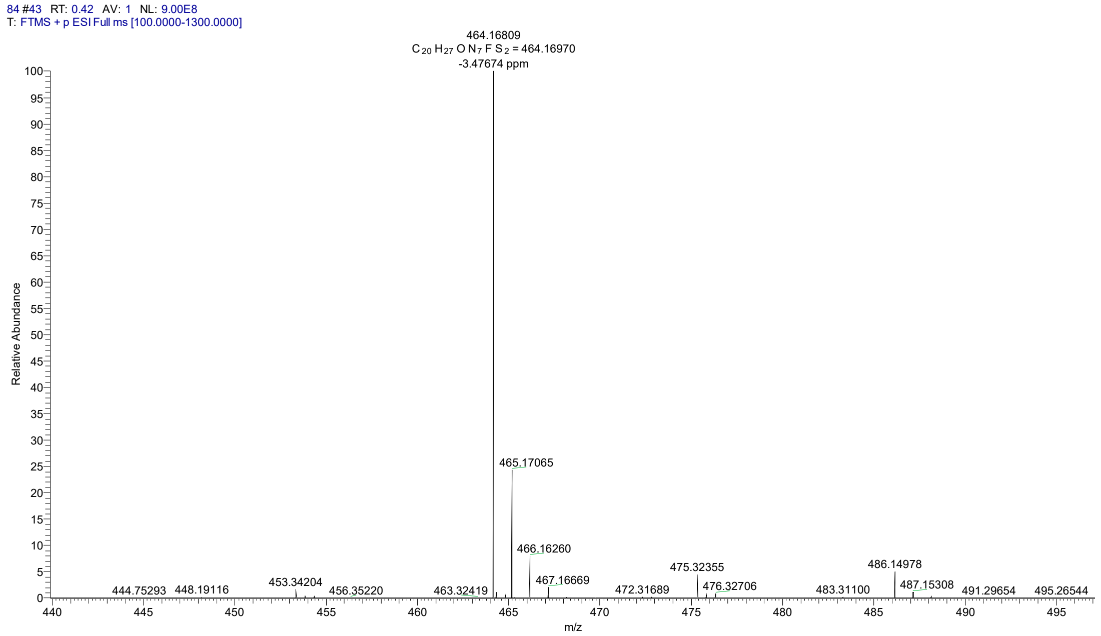

Figure S56. HRMS of compound **C17**.

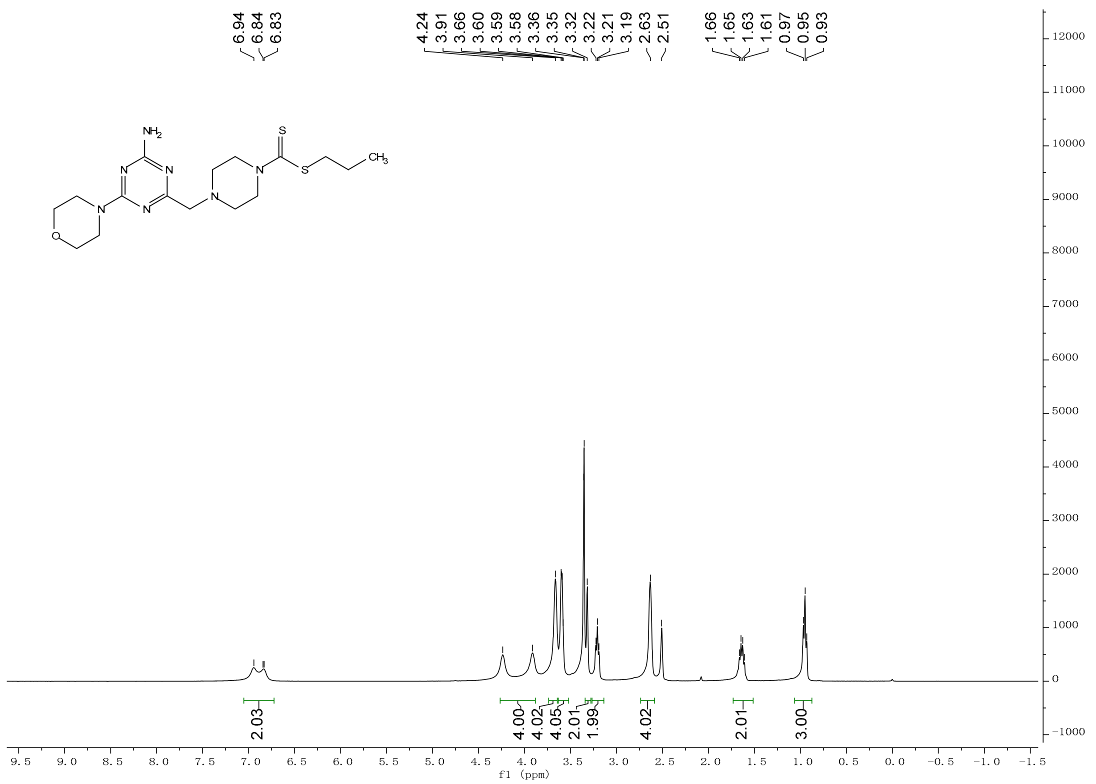

Figure S57. ^{1}H NMR (500 MHz, DMSO-d_6) spectrum of compound **C18**.

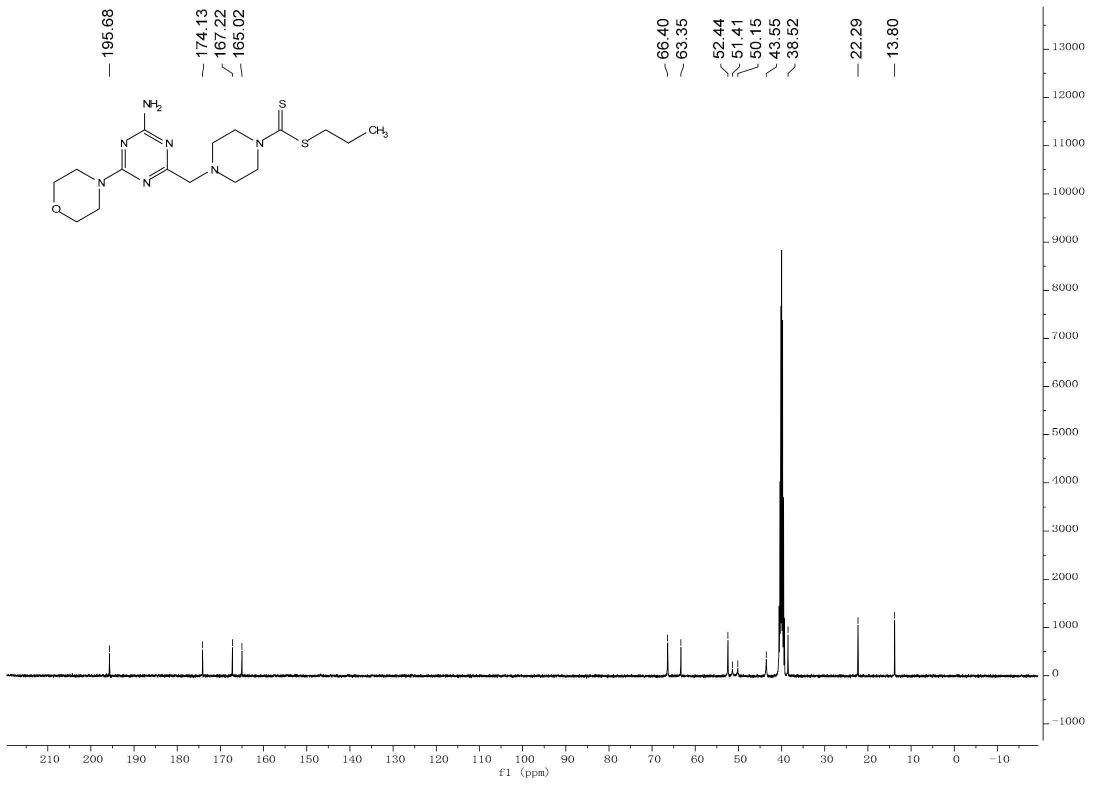

Figure S58. ^{13}C NMR (126 MHz, DMSO-d_6) spectrum of compound **C18**.

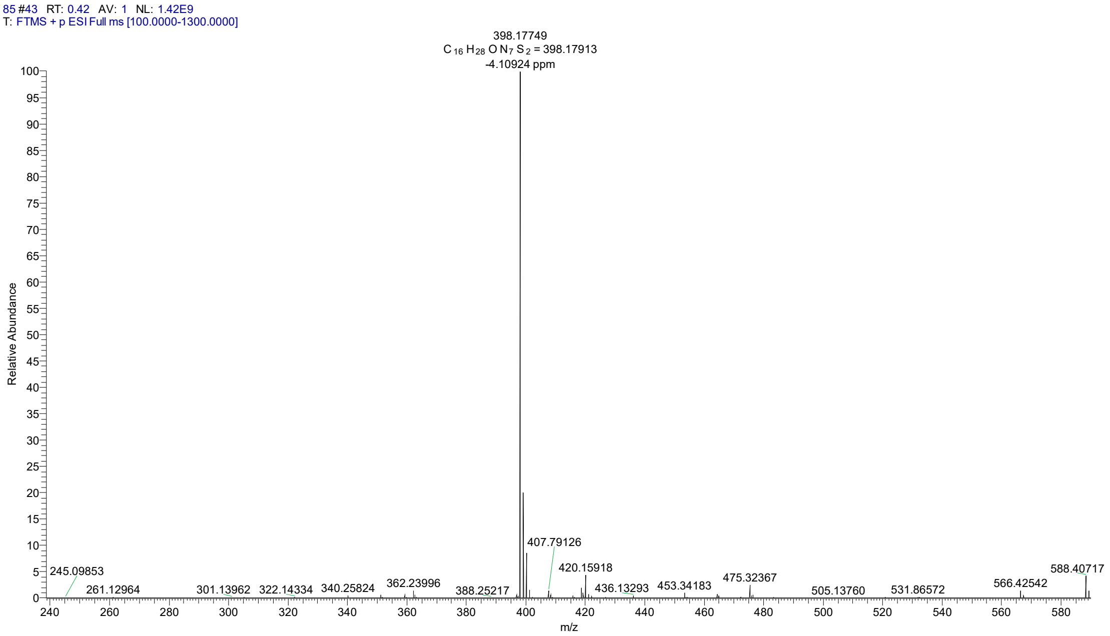

Figure S59. HRMS of compound **C18**.

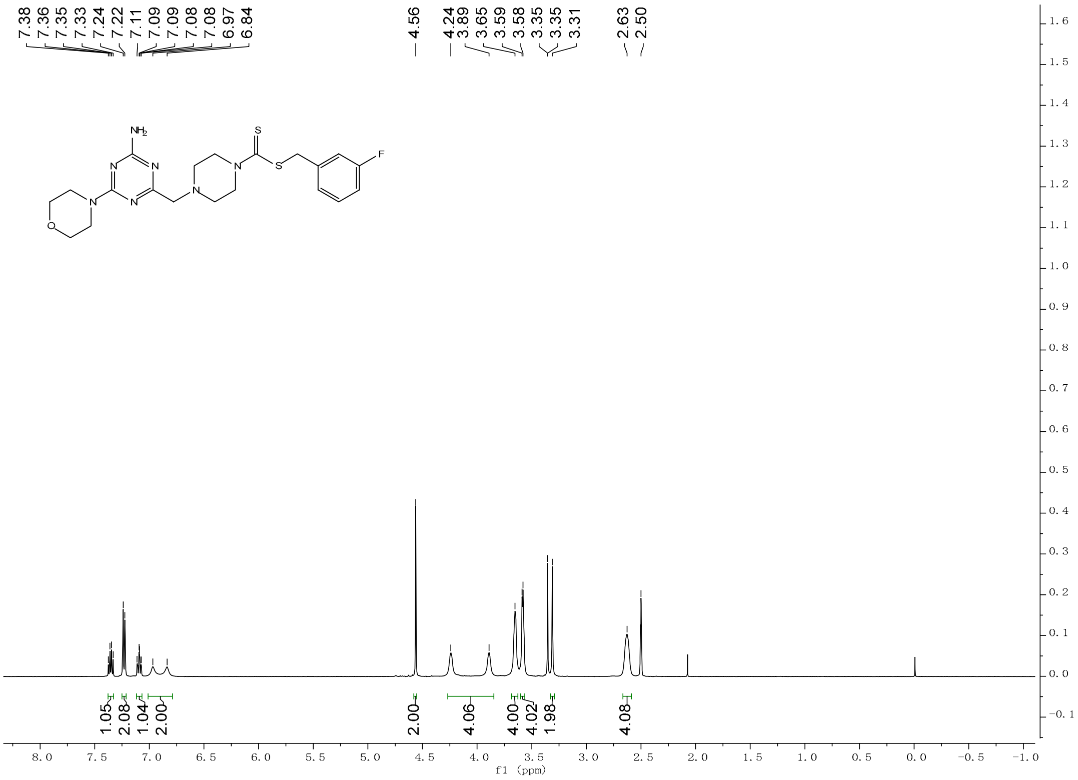

Figure S60. ^{1}H NMR (500 MHz, DMSO-d_6) spectrum of compound **C19**.

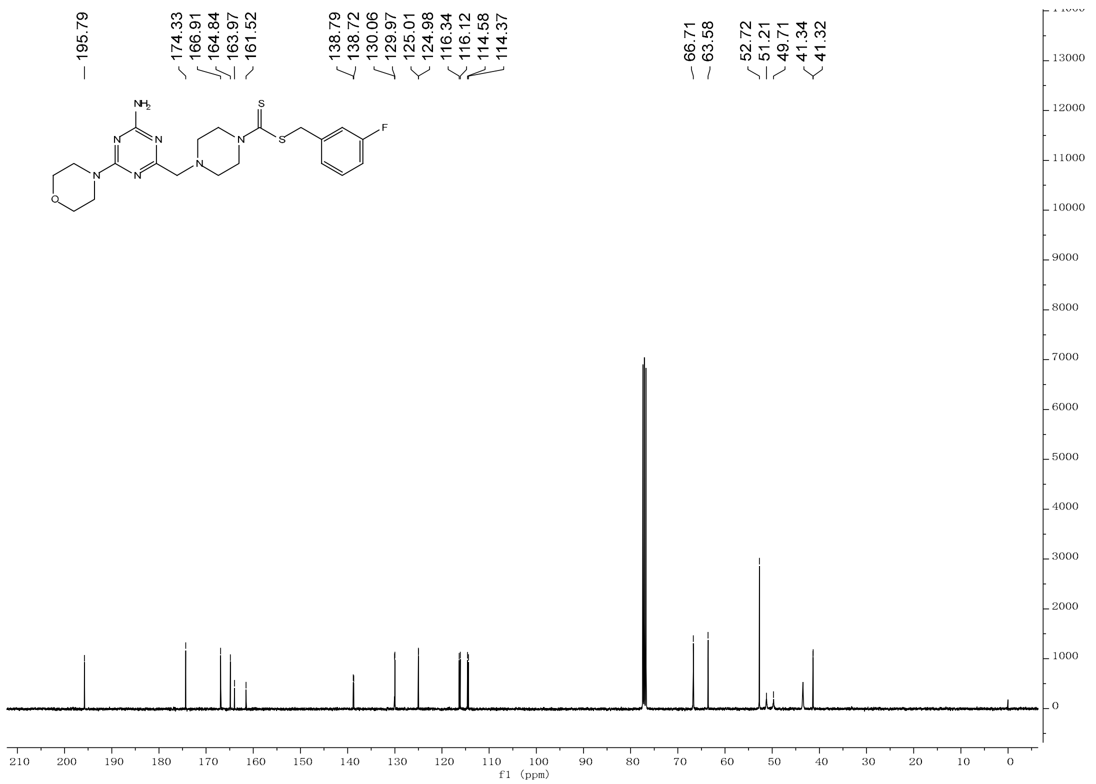

Figure S61. ^{13}C NMR (126 MHz, DMSO-d_6) spectrum of compound **C19**.

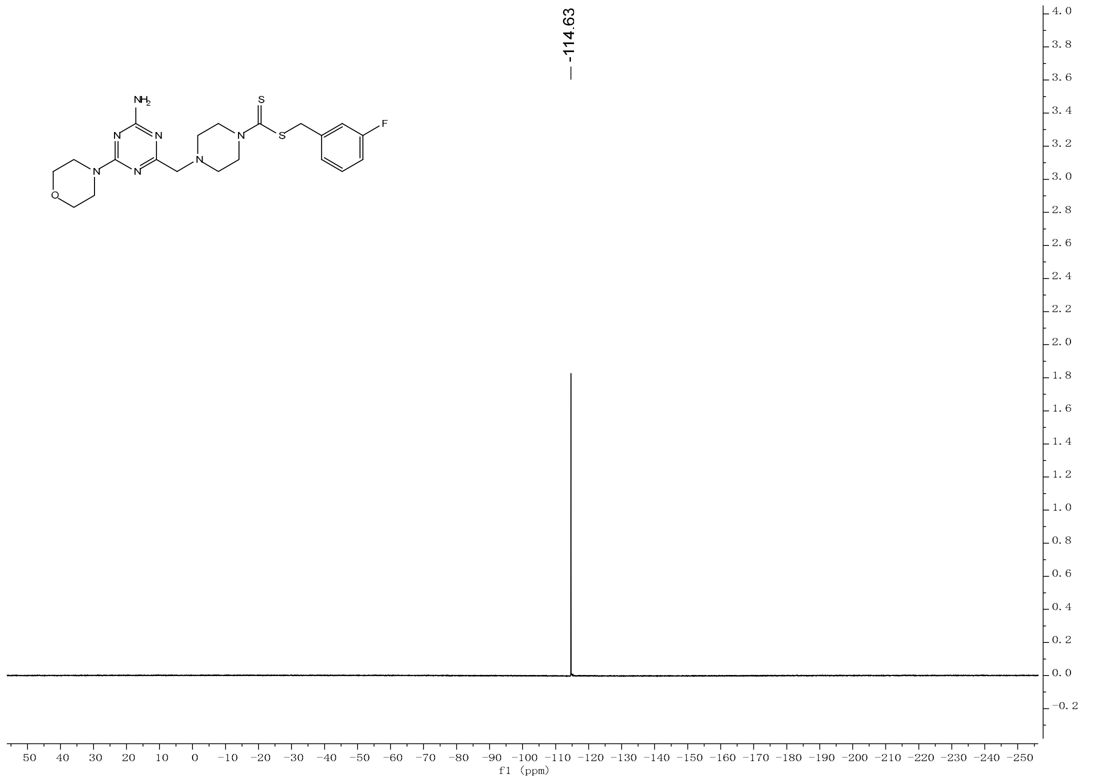

Figure S62. ^{19}F NMR (471 MHz, $CDCl_3$) spectrum of compound **C19**.

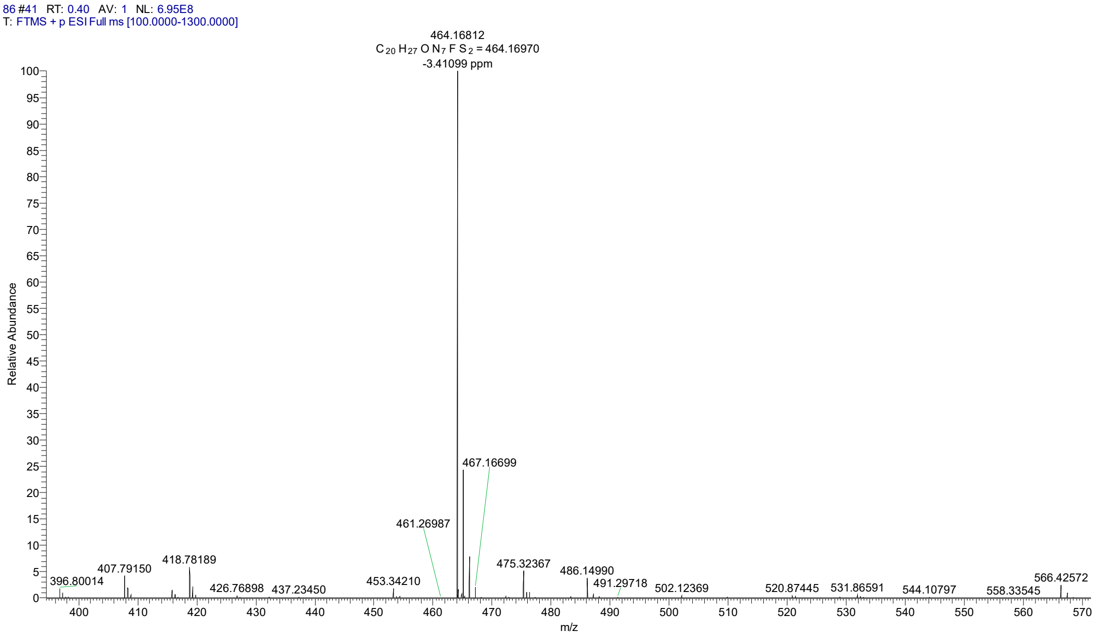

Figure S63. HRMS of compound **C19**.

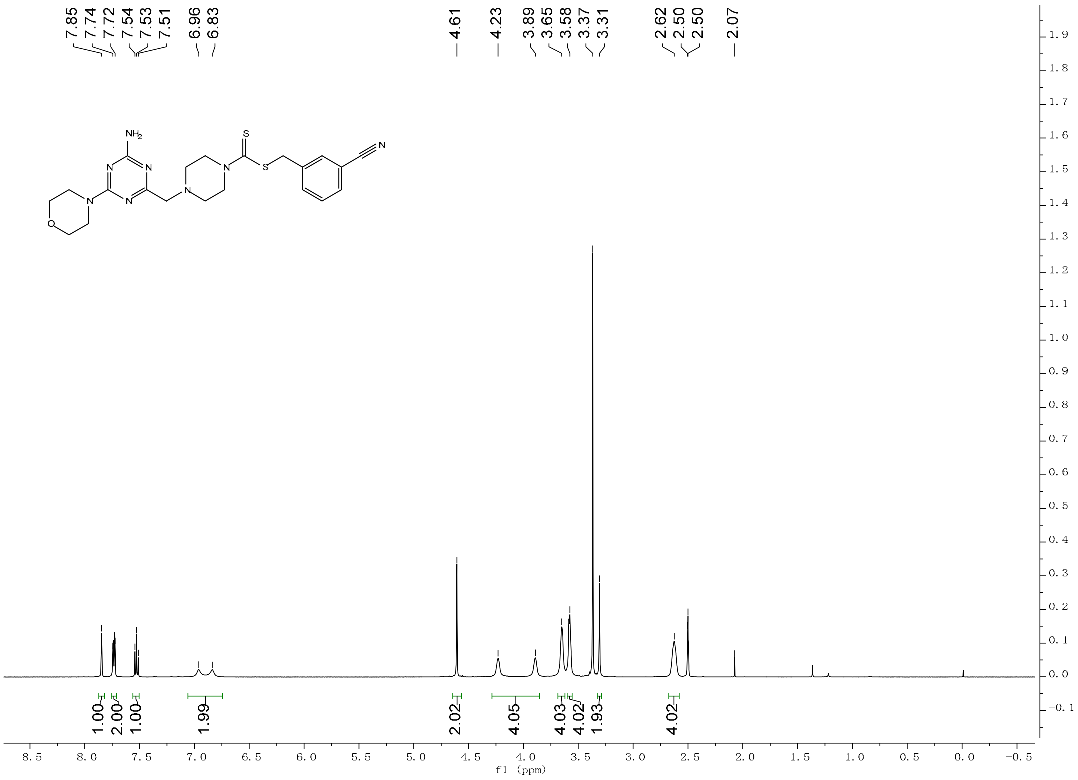

Figure S64. ^{1}H NMR (500 MHz, DMSO-d_6) spectrum of compound **C20**.

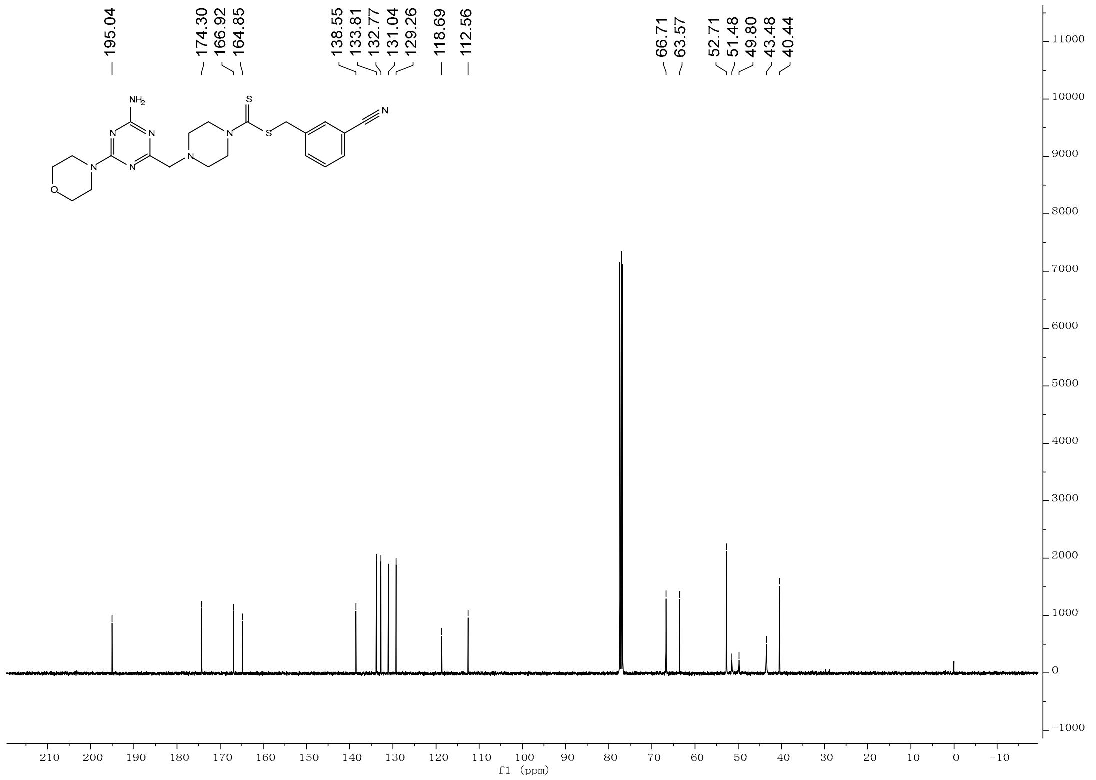

Figure S65. ^{13}C NMR (126 MHz, DMSO-d_6) spectrum of compound **C20**.

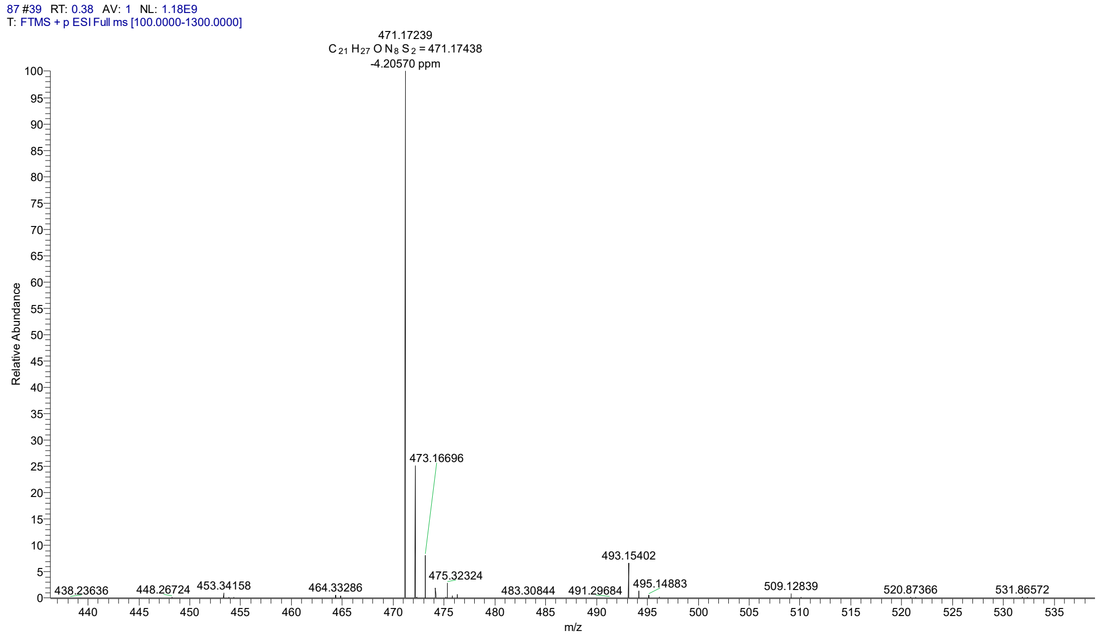

Figure S66. HRMS of compound **C20**.

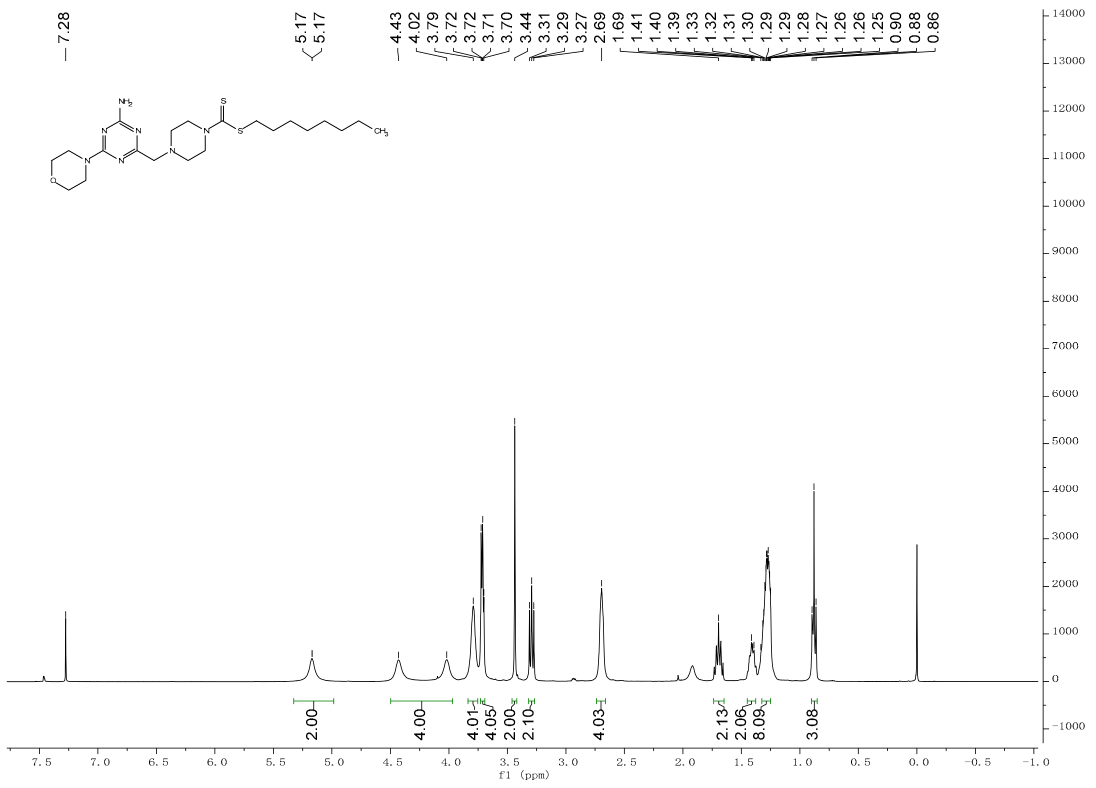

Figure S67. ^{1}H NMR (500 MHz, DMSO-d_6) spectrum of compound **C21**.

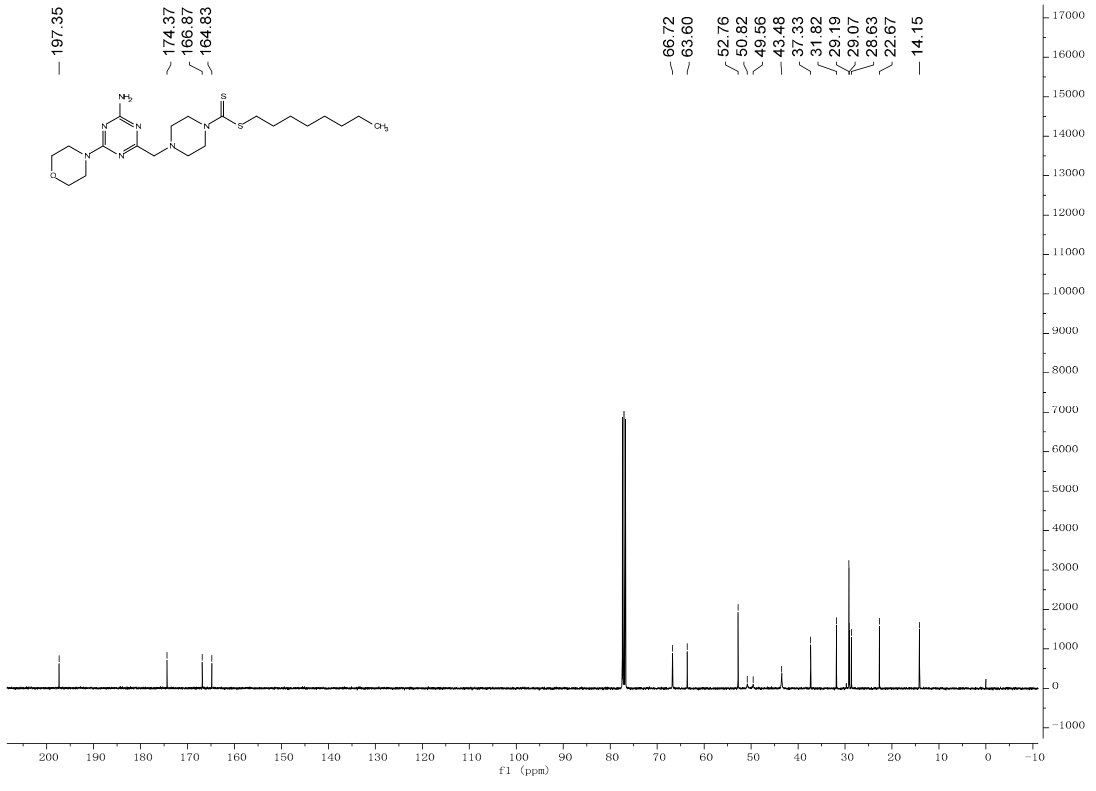

Figure S68. ^{13}C NMR (101 MHz, $CDCl_3$) spectrum of compound **C21**.

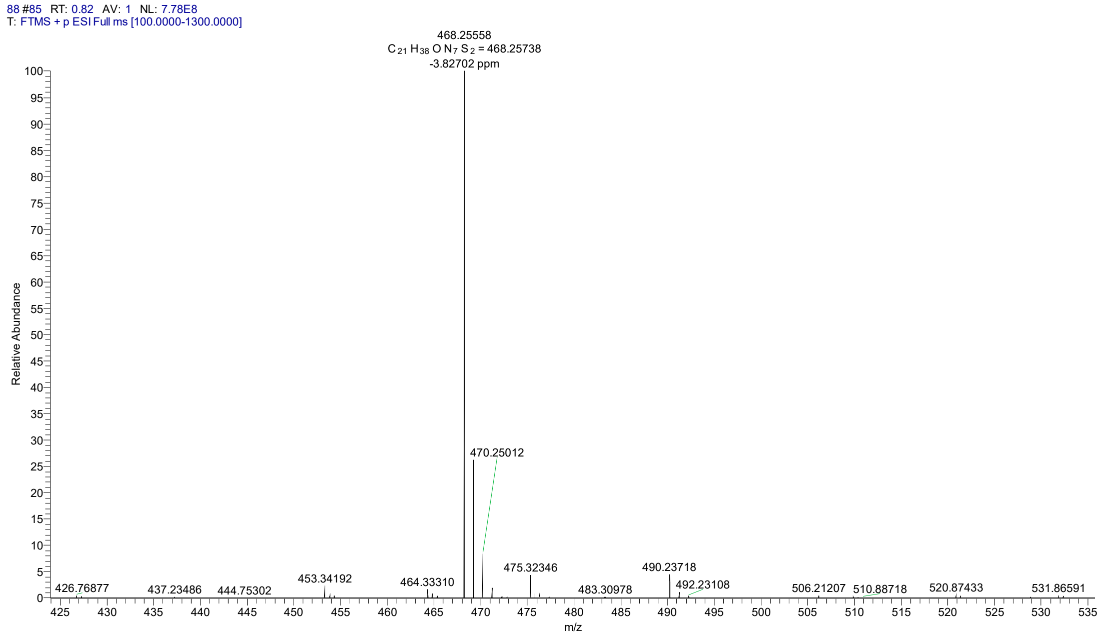

Figure S69. HRMS of compound **C21**.

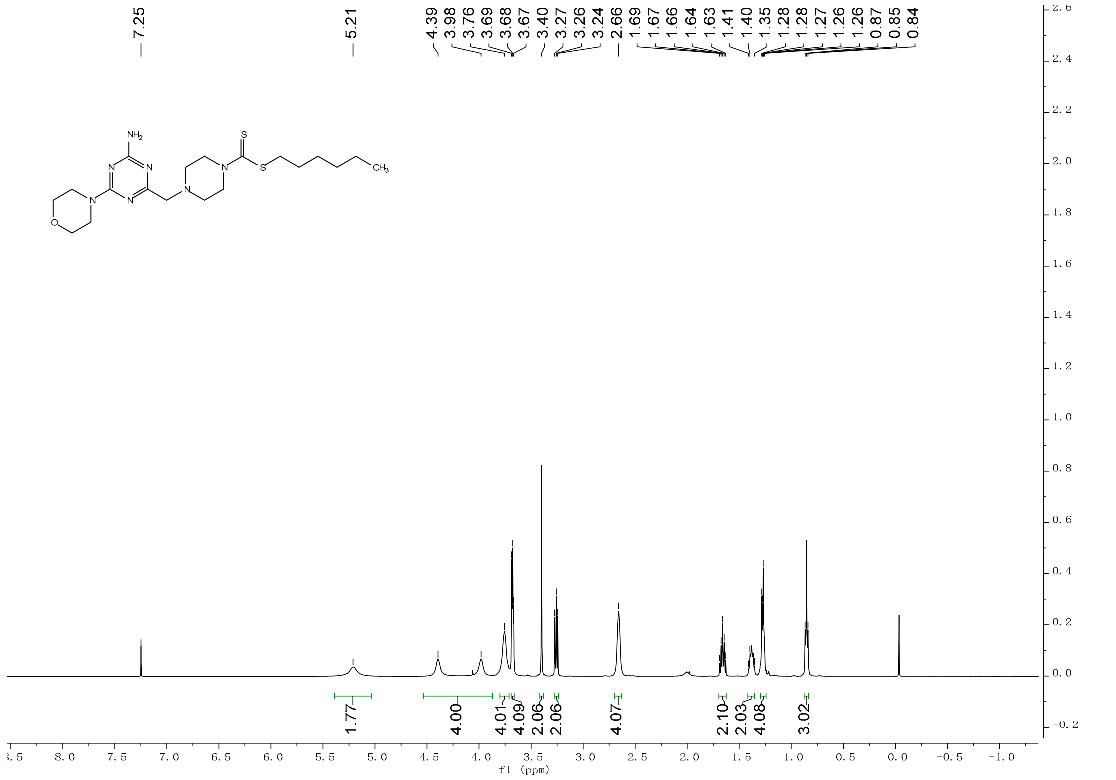

Figure S70. ^{1}H NMR (500 MHz, $CDCl_3$) spectrum of compound **C22**.

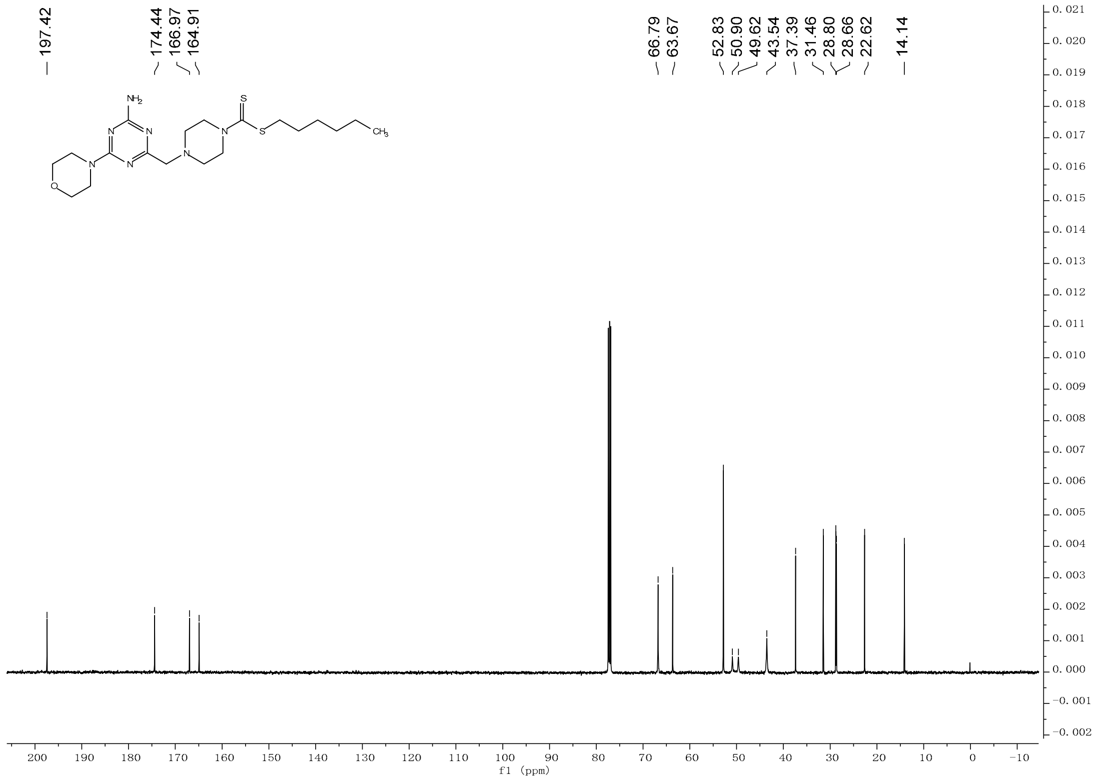

Figure S71. ^{13}C NMR (126MHz, $CDCl_3$) spectrum of compound **C22**.

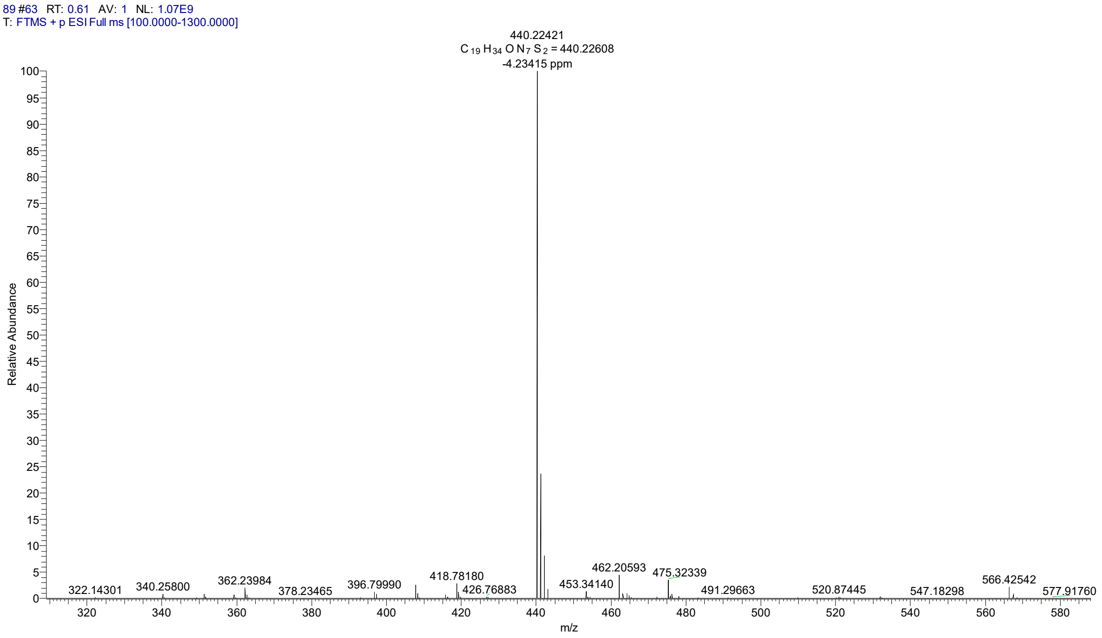

Figure S72. HRMS of compound **C22**.

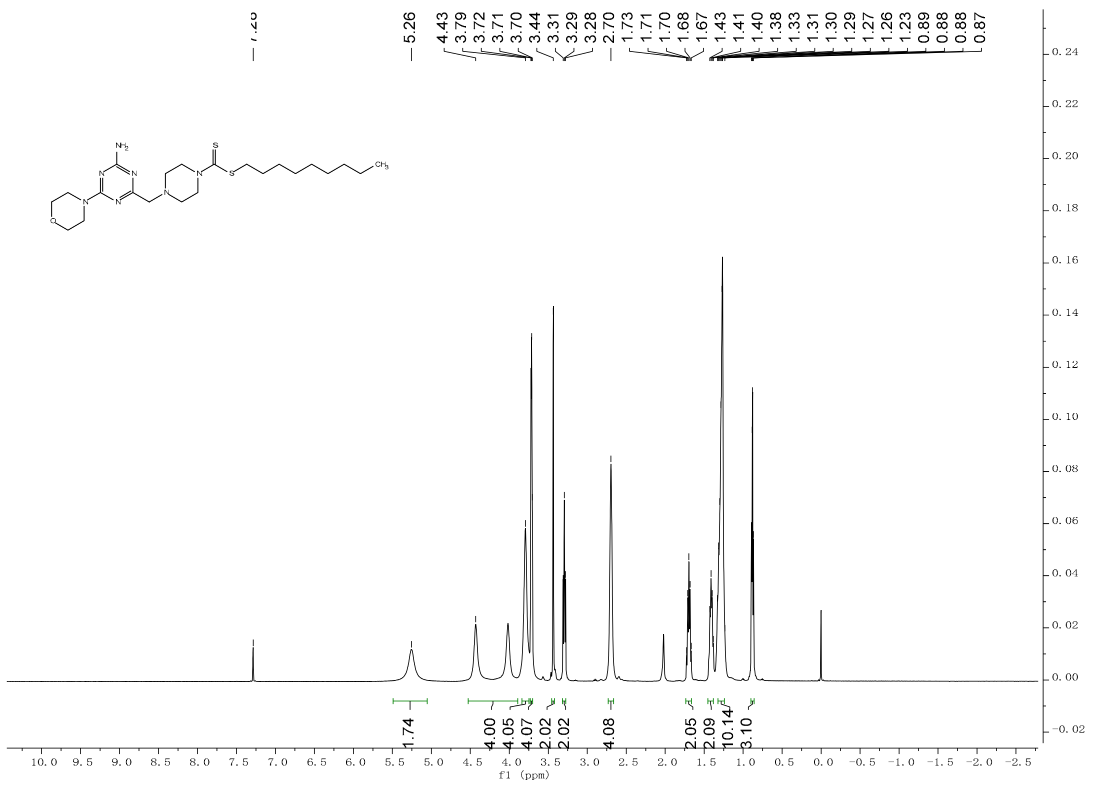

Figure S73. ^{1}H NMR (500 MHz, $CDCl_3$) spectrum of compound **C23**.

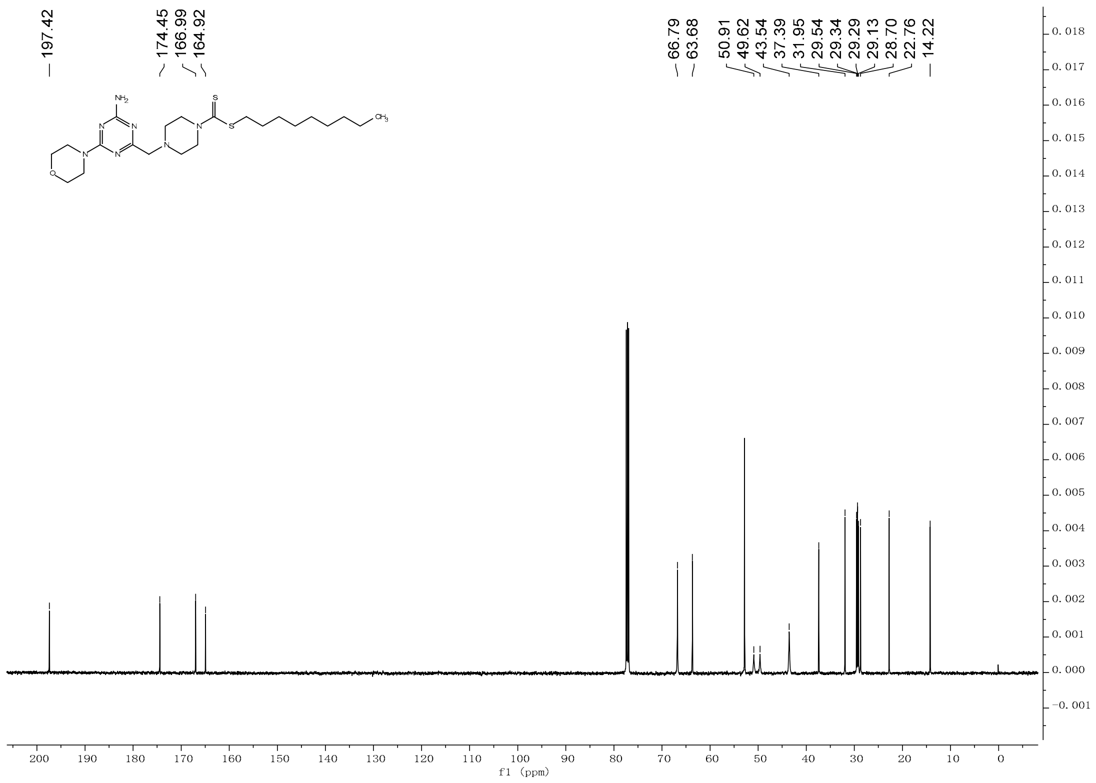

Figure S74. ^{13}C NMR (126 MHz, $CDCl_3$) spectrum of compound **C23**.

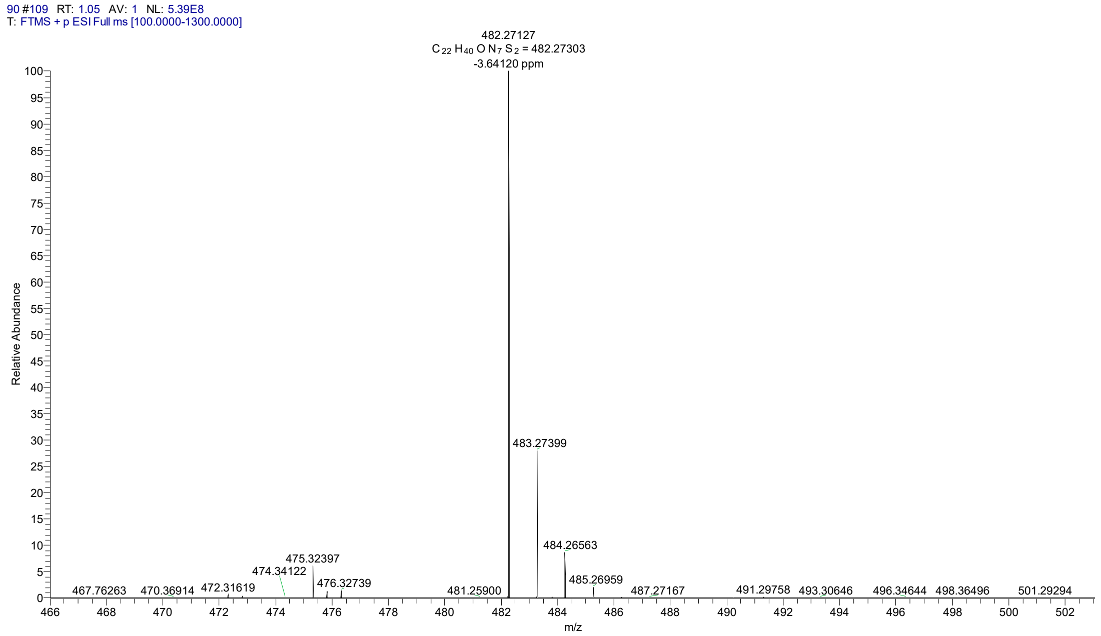

Figure S75. HRMS of compound **C23**.

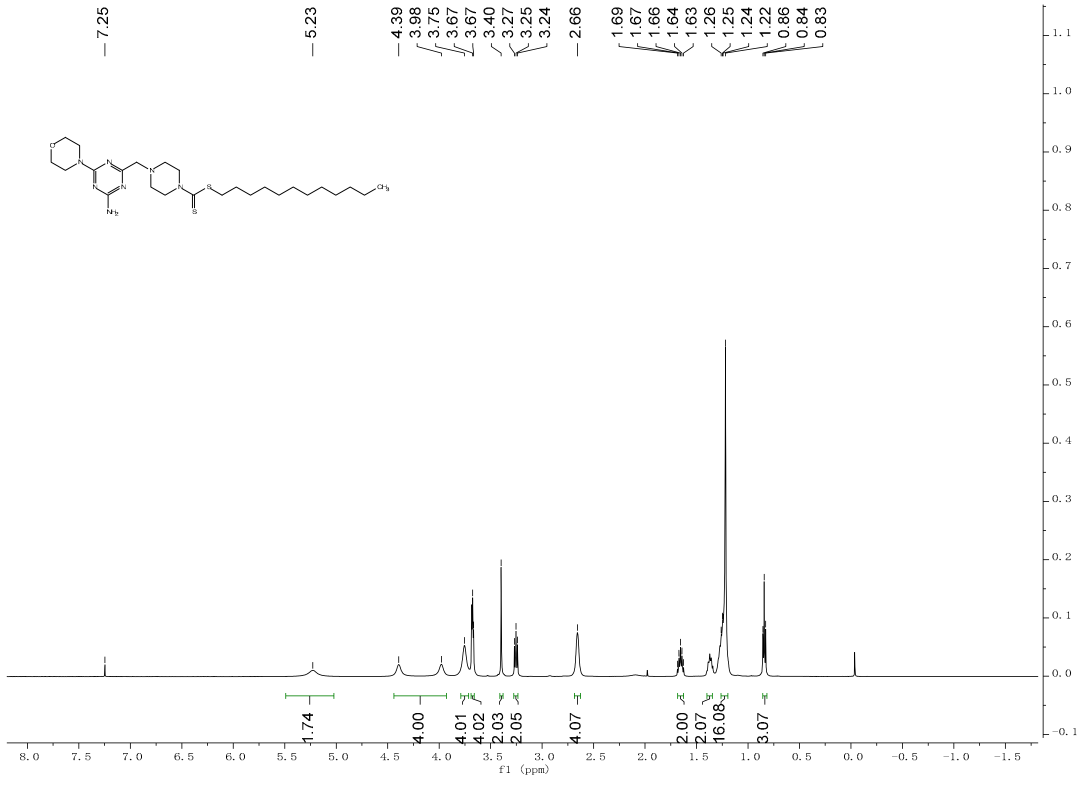

Figure S76. ^{1}H NMR (500 MHz, $CDCl_3$) spectrum of compound **C24**.

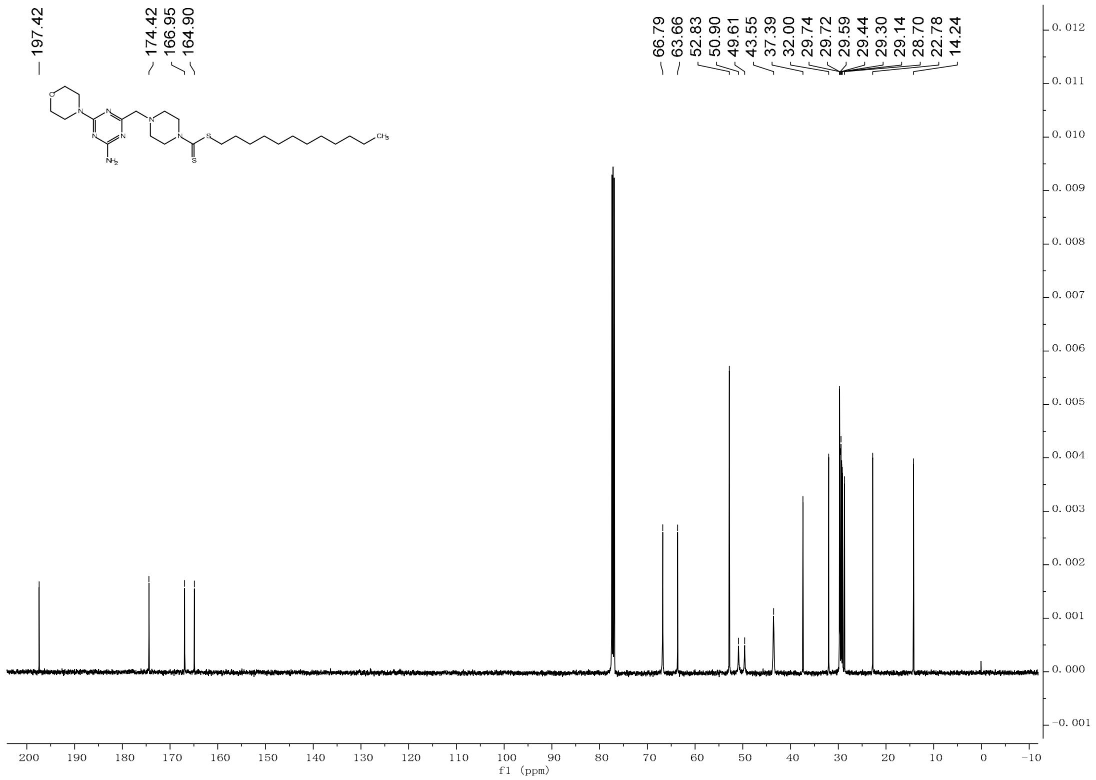

Figure S77. ^{13}C NMR (126 MHz, $CDCl_3$) spectrum of compound **C24**.

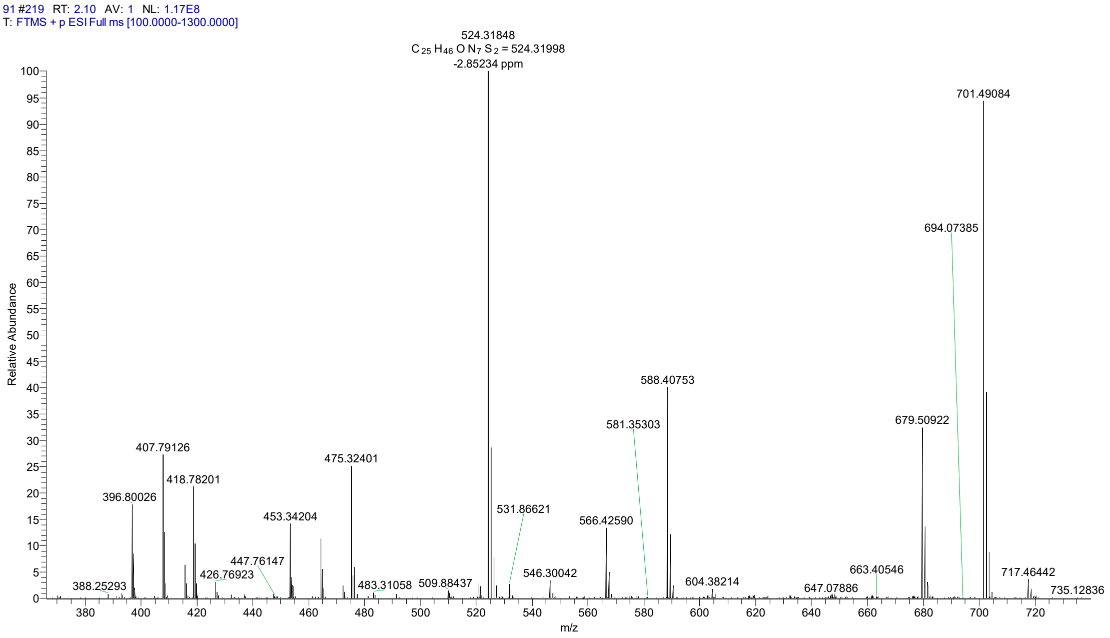

Figure S78. HRMS of compound **C24**.

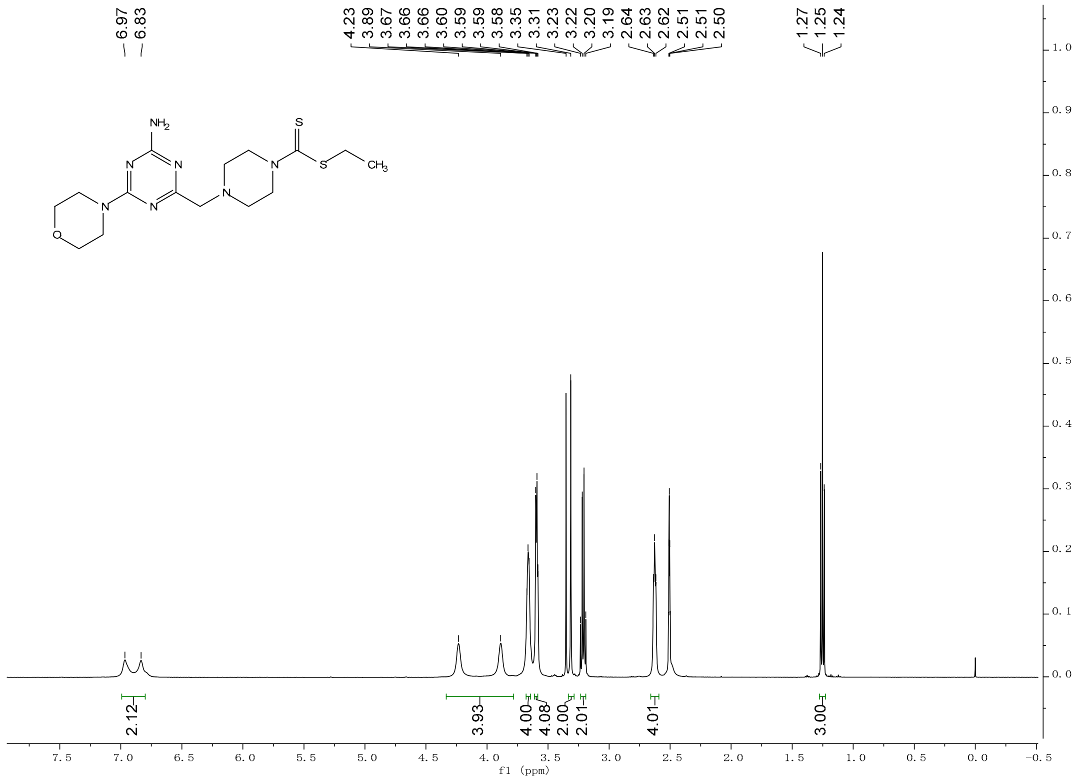

Figure S79. ^{1}H NMR (500 MHz, DMSO-d_6) spectrum of compound **C25**.

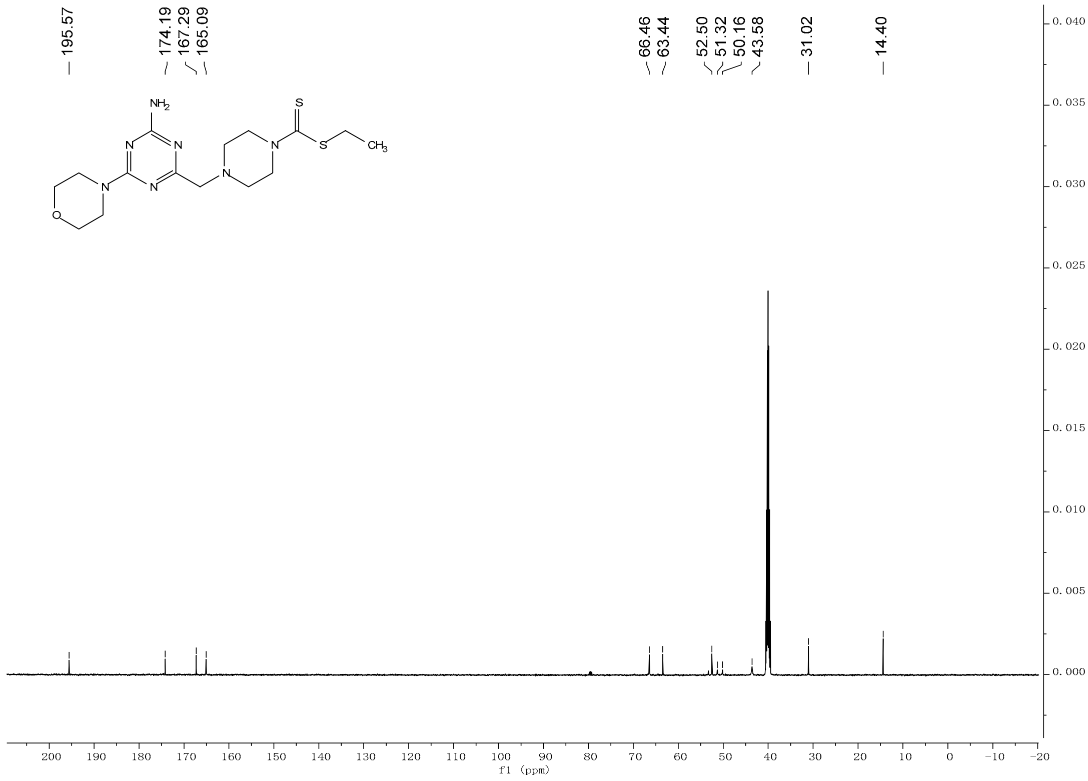

Figure S80. ^{13}C NMR (126 MHz, DMSO-d_6) spectrum of compound **C25**.

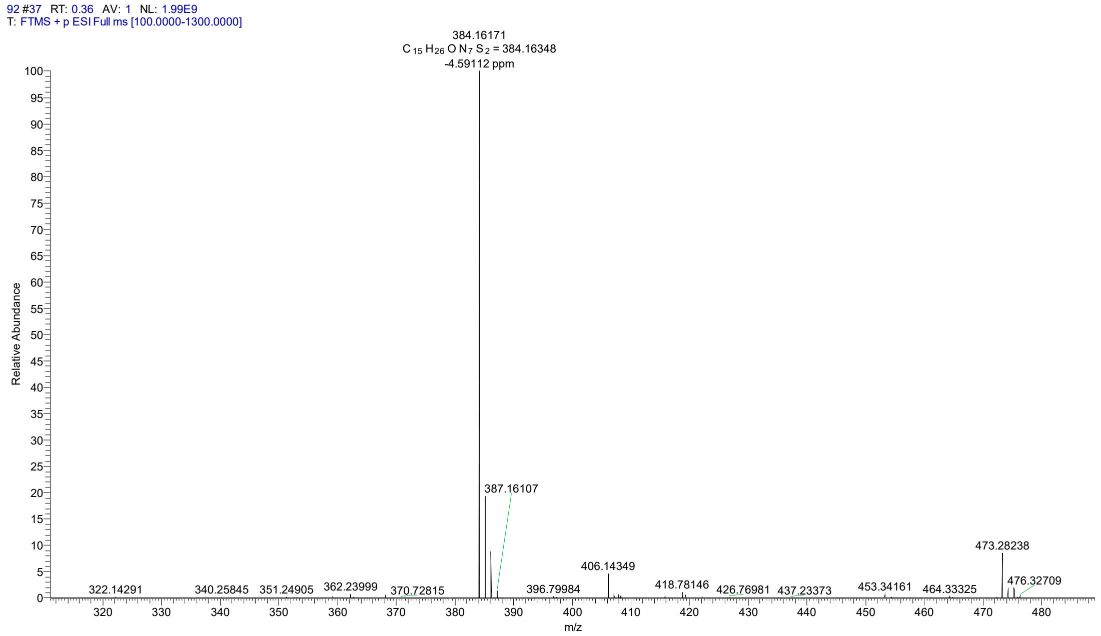

Figure S81. HRMS of compound **C25**.

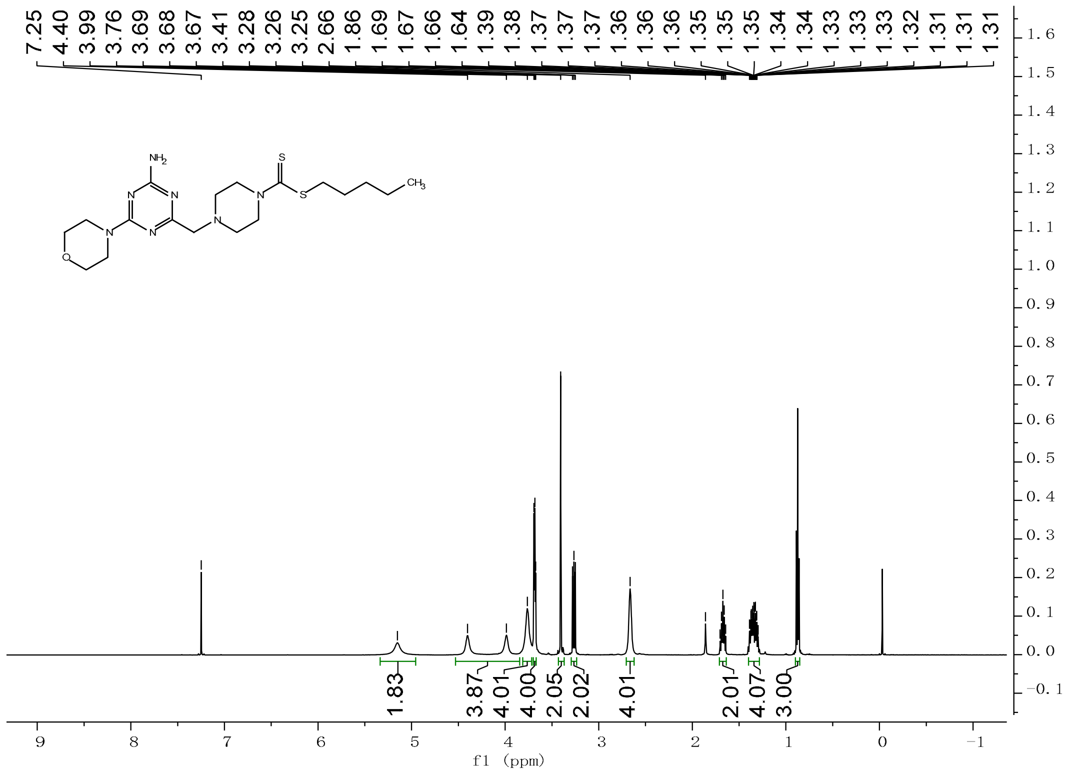

Figure S82. ^{1}H NMR (500 MHz, $CDCl_3$) spectrum of compound **C26**.

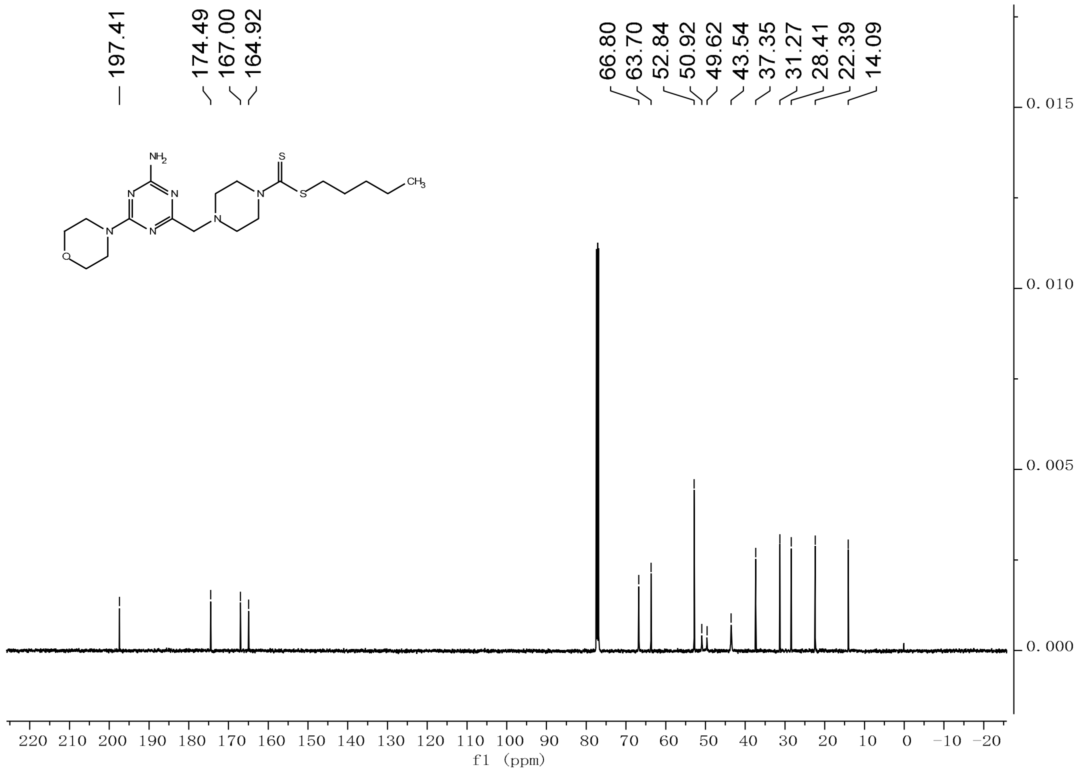

Figure S83. ^{13}C NMR (126 MHz, $CDCl_3$) spectrum of compound **C26**.

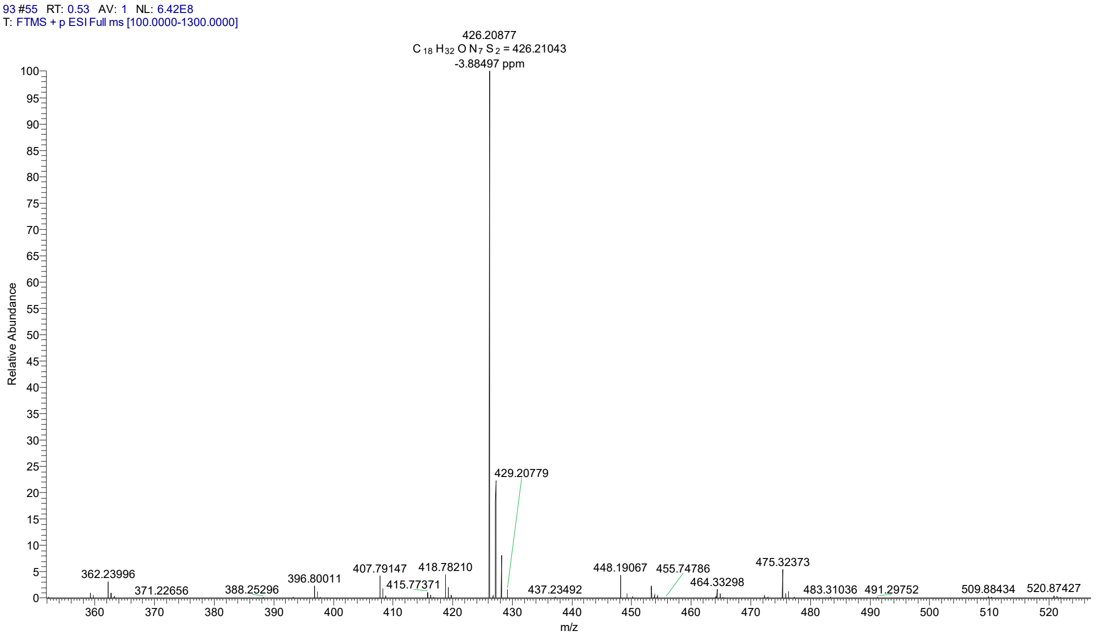

Figure S84. HRMS of compound **C26**.

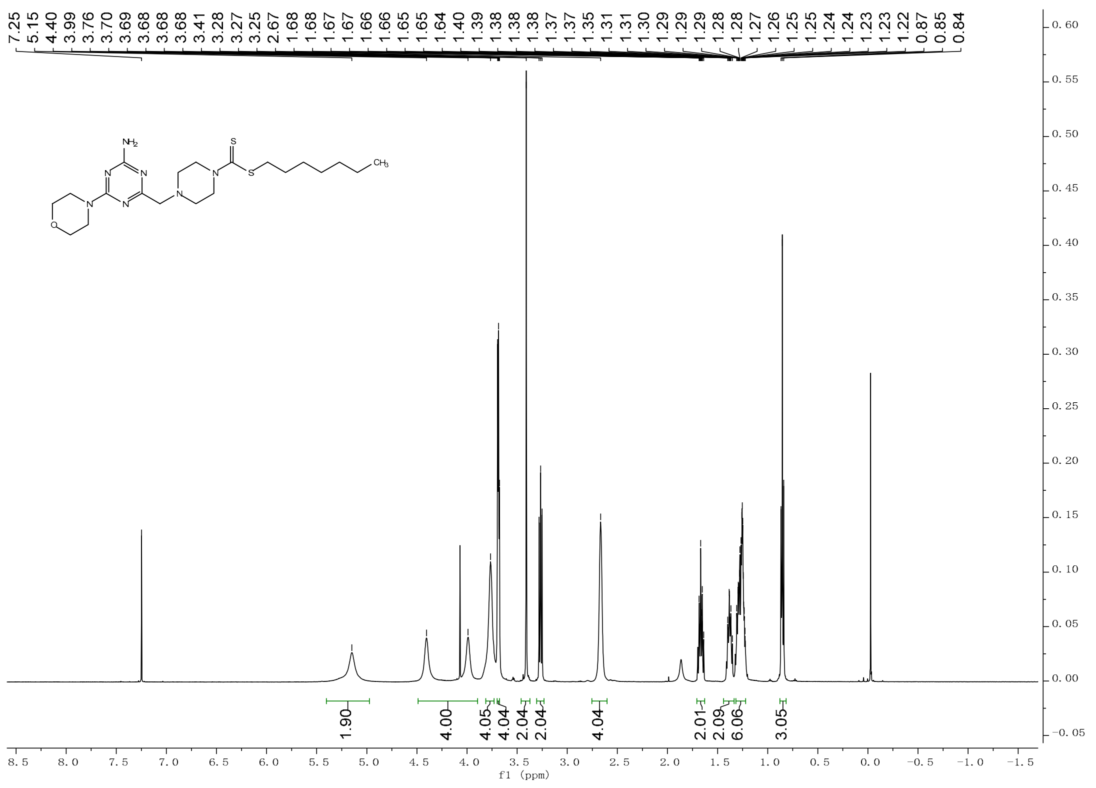

Figure S85. ^{1}H NMR (500 MHz, $CDCl_3$) spectrum of compound **C27**.

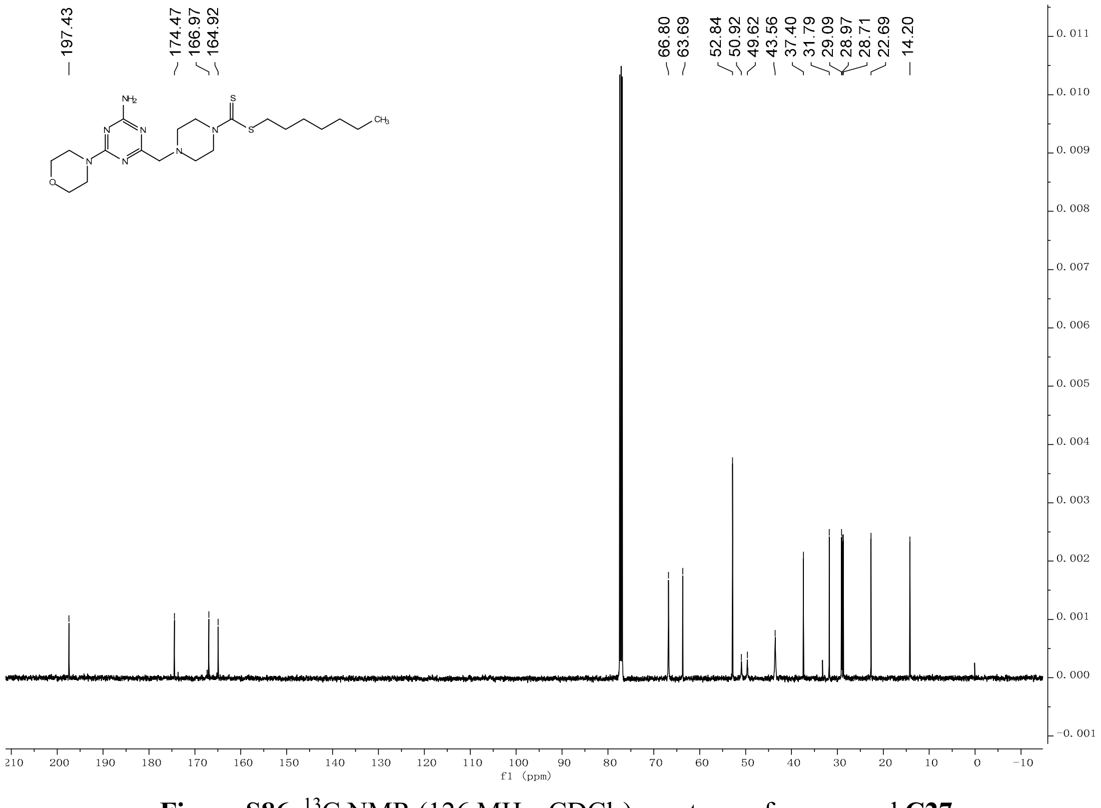

Figure S86. ^{13}C NMR (126 MHz, $CDCl_3$) spectrum of compound **C27**.

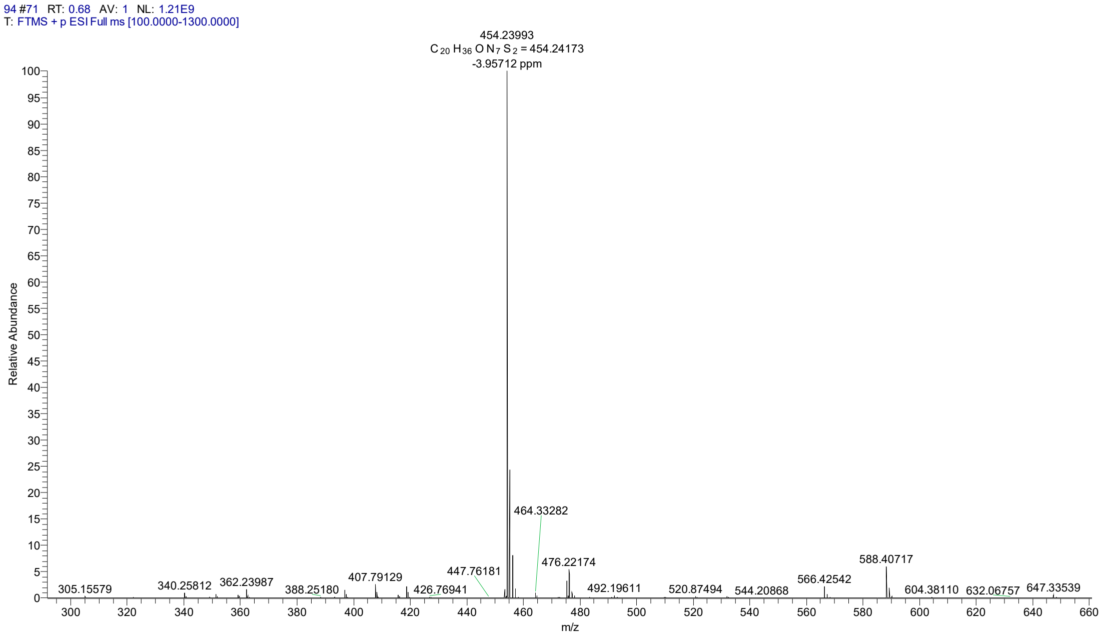

Figure S87. HRMS of compound **C27**.

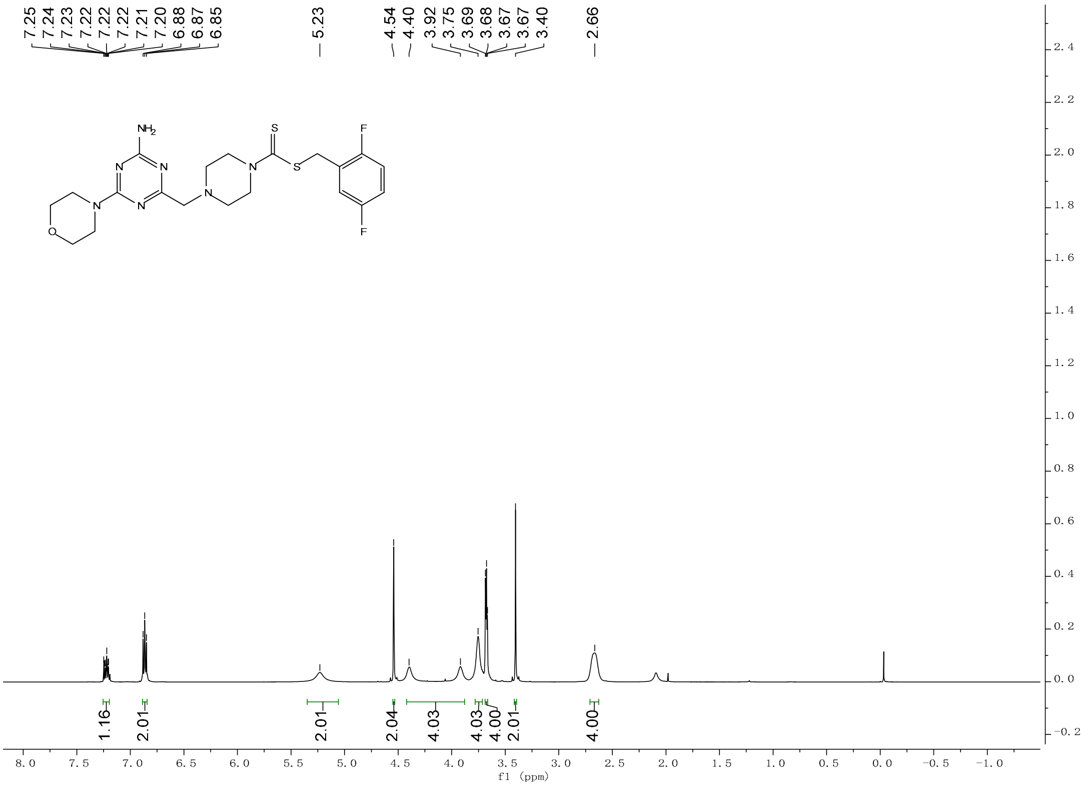

Figure S88. ^{1}H NMR (500 MHz, $CDCl_3$) spectrum of compound **C28**.

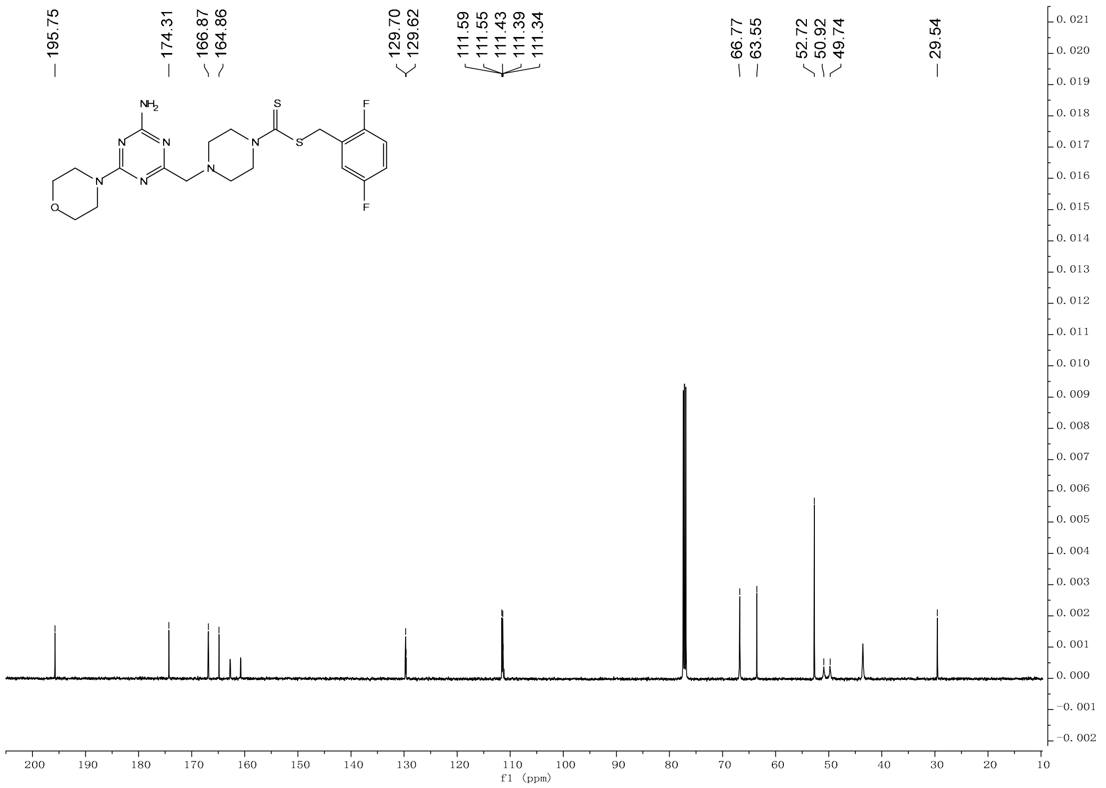

Figure S89. ^{13}C NMR (126 MHz, CDCl$_3$) spectrum of compound **C28**.

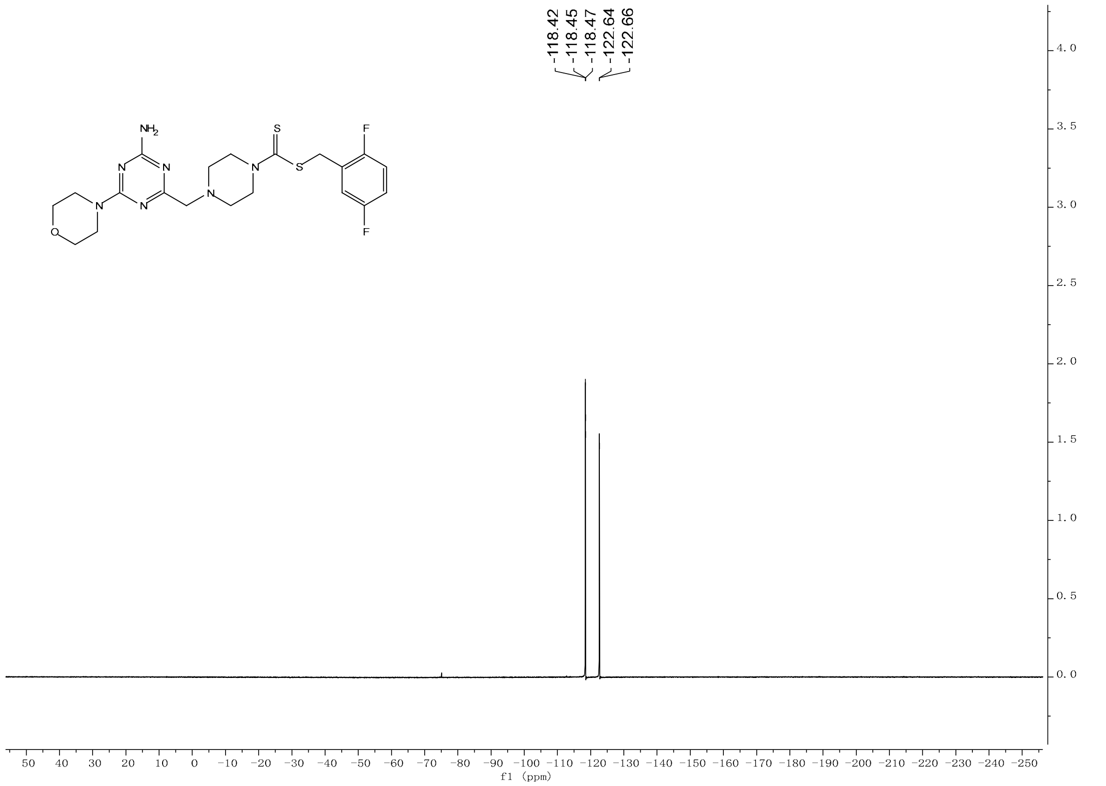

Figure S90. ^{19}F NMR (471 MHz, CDCl$_3$) spectrum of compound **C28**.

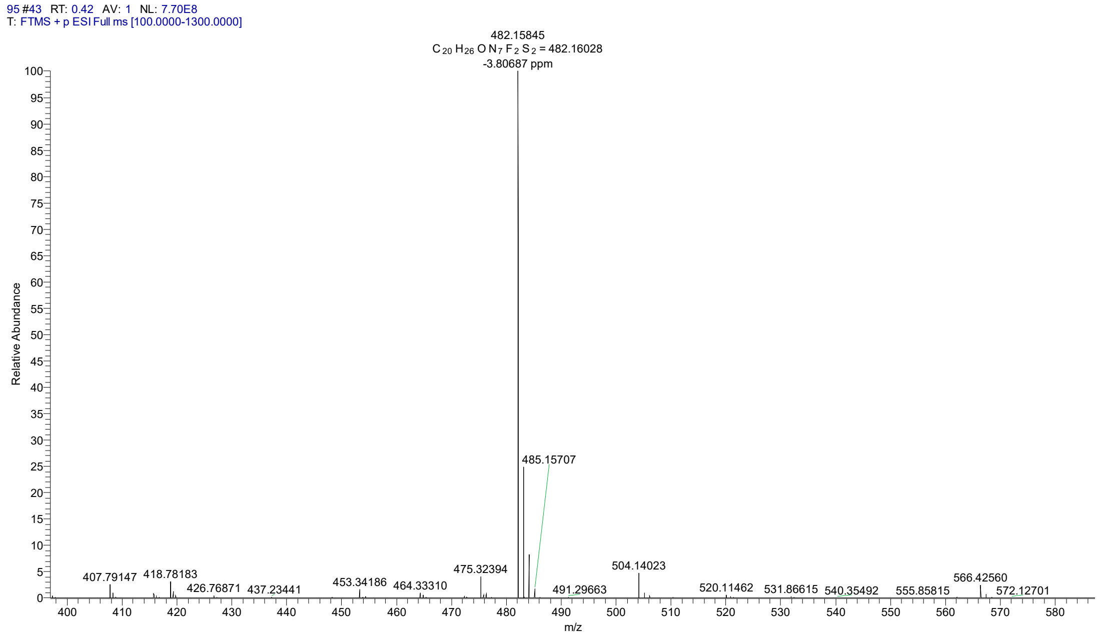

Figure S91. HRMS of compound **C28**.

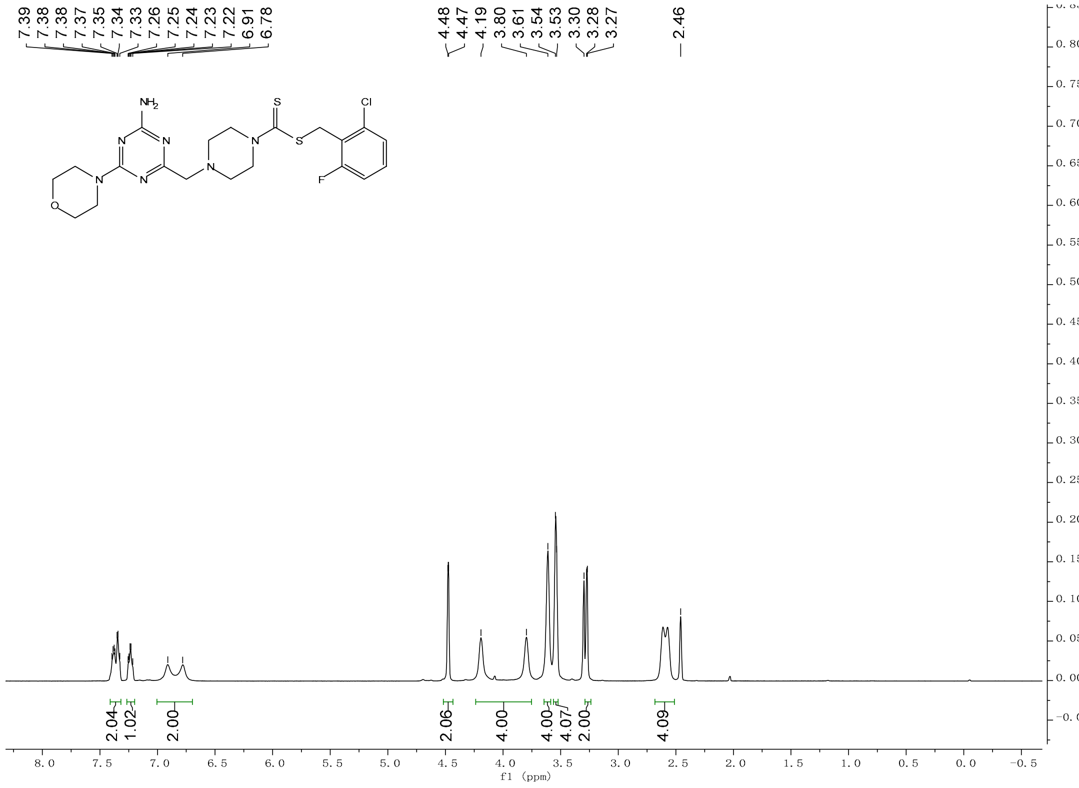

Figure S92. ^{1}H NMR (500 MHz, DMSO-d_6) spectrum of compound **C29**.

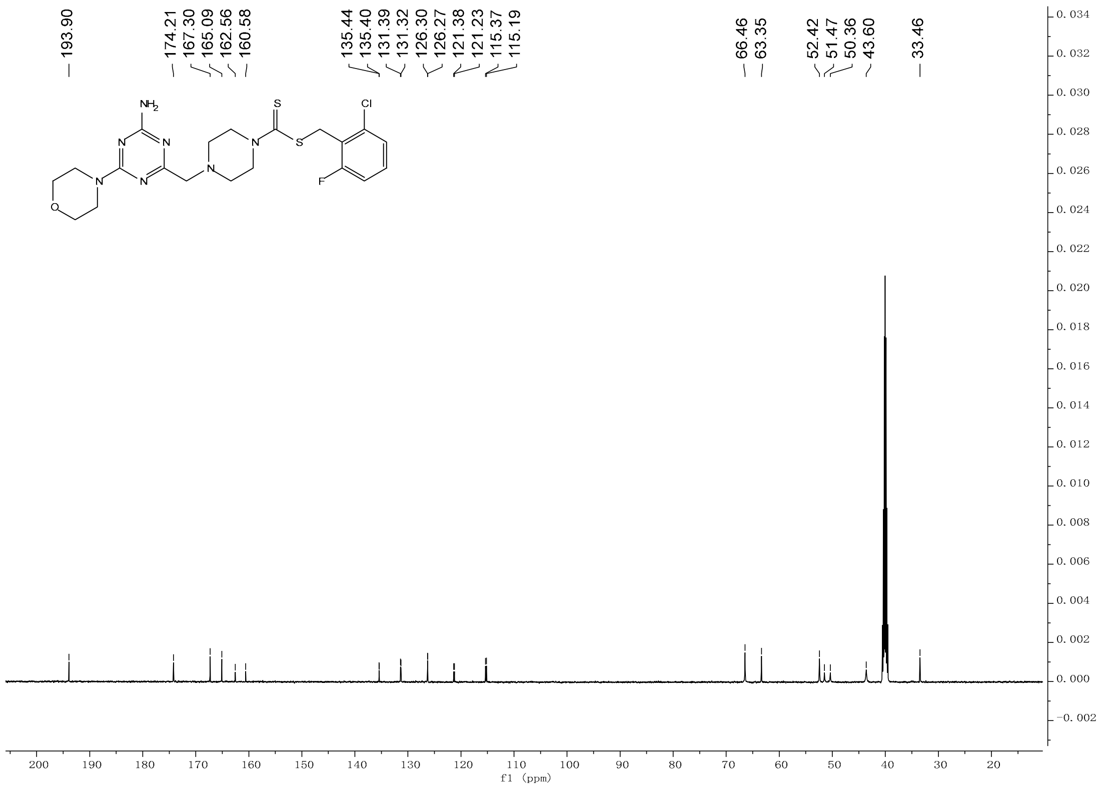

Figure S93. ^{13}C NMR (126 MHz, DMSO-d_6) spectrum of compound **C29**.

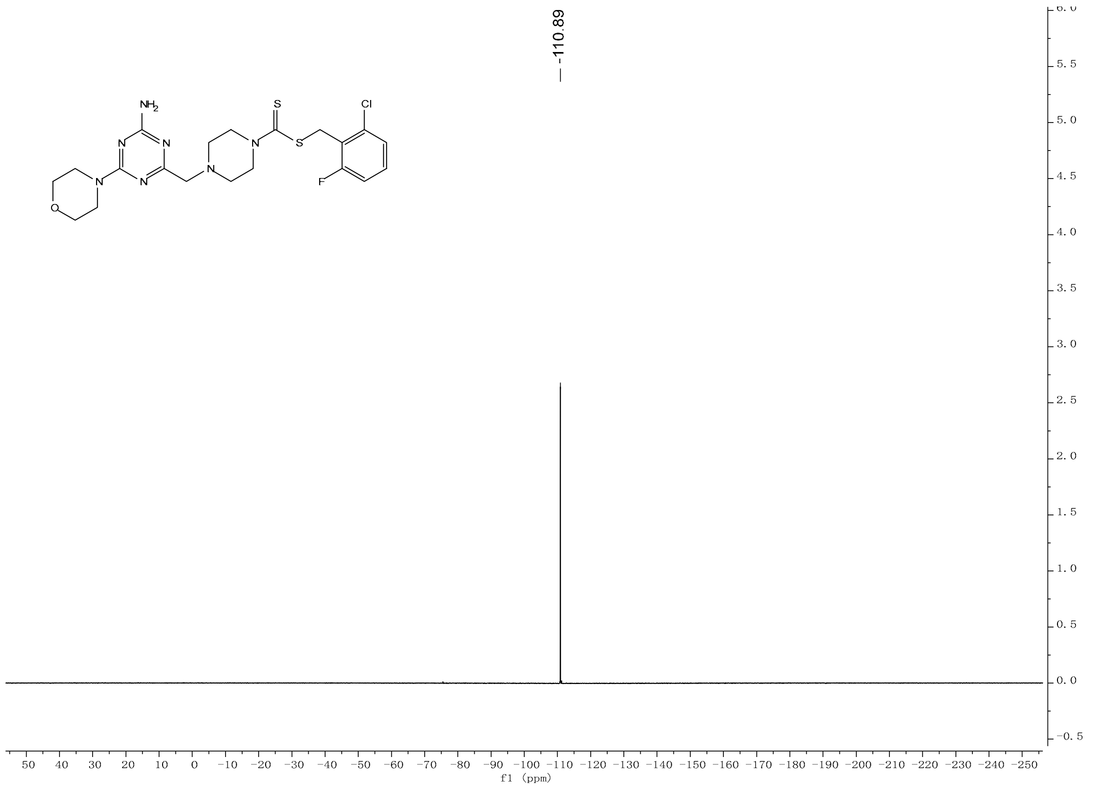

Figure S94. ^{19}F NMR (500 MHz, $CDCl_3$) spectrum of compound **C29**.

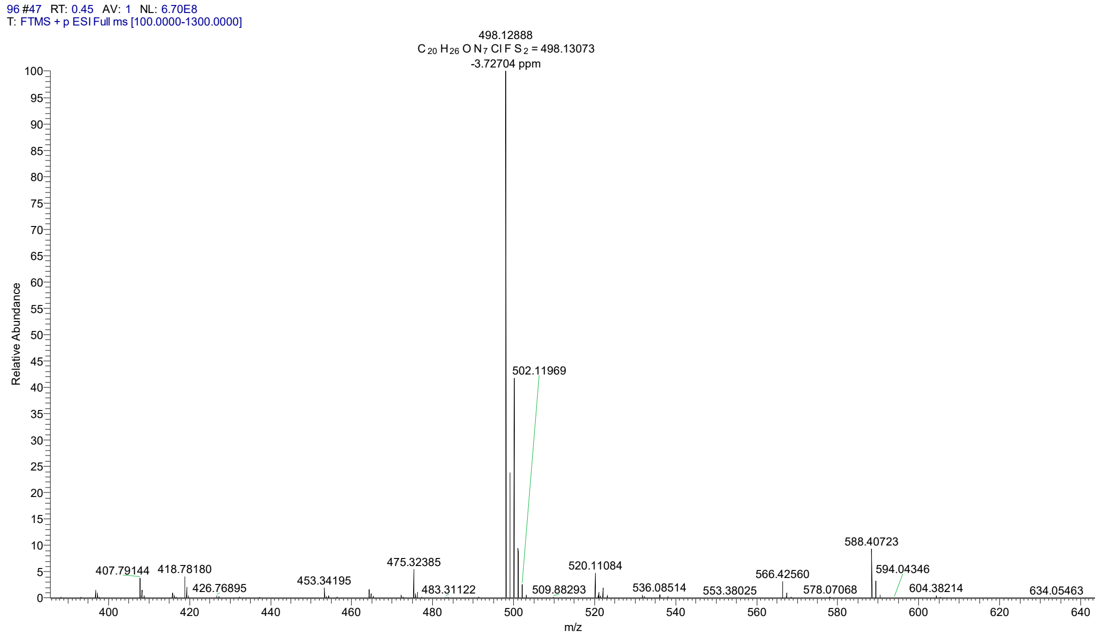

Figure S95. HRMS of compound **C29**.

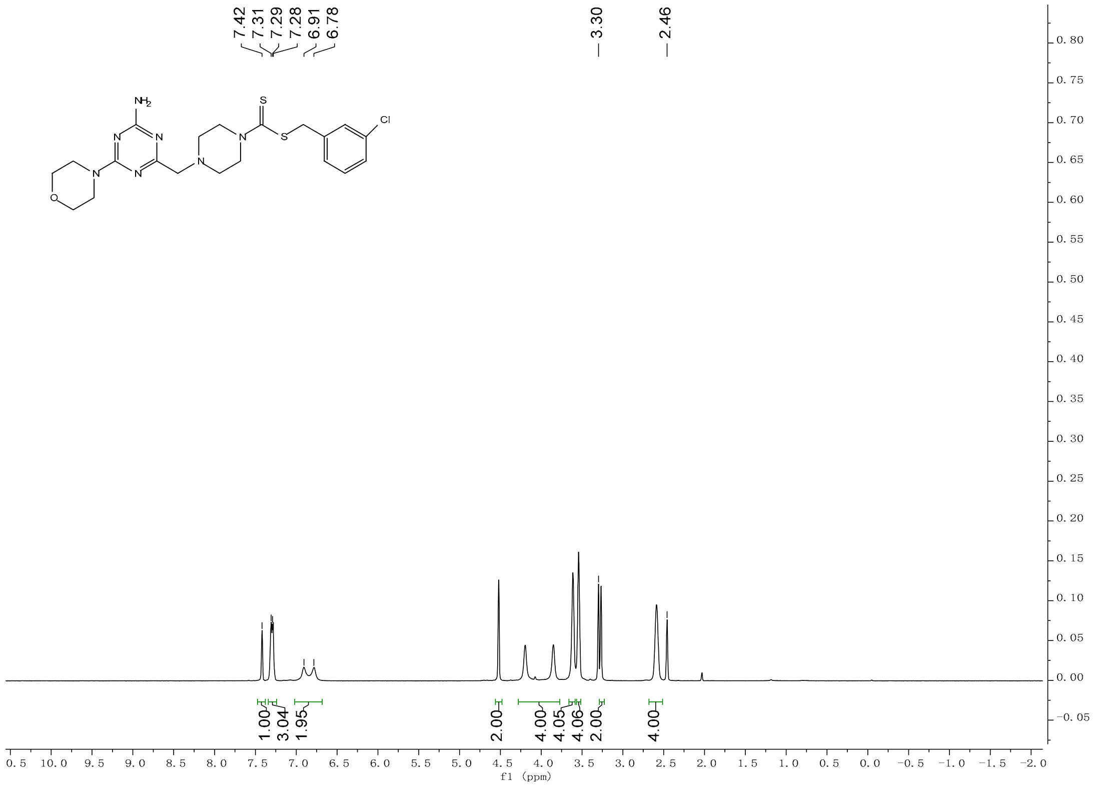

Figure S96. ^{1}H NMR (500 MHz, DMSO-d_6) spectrum of compound **C30**.

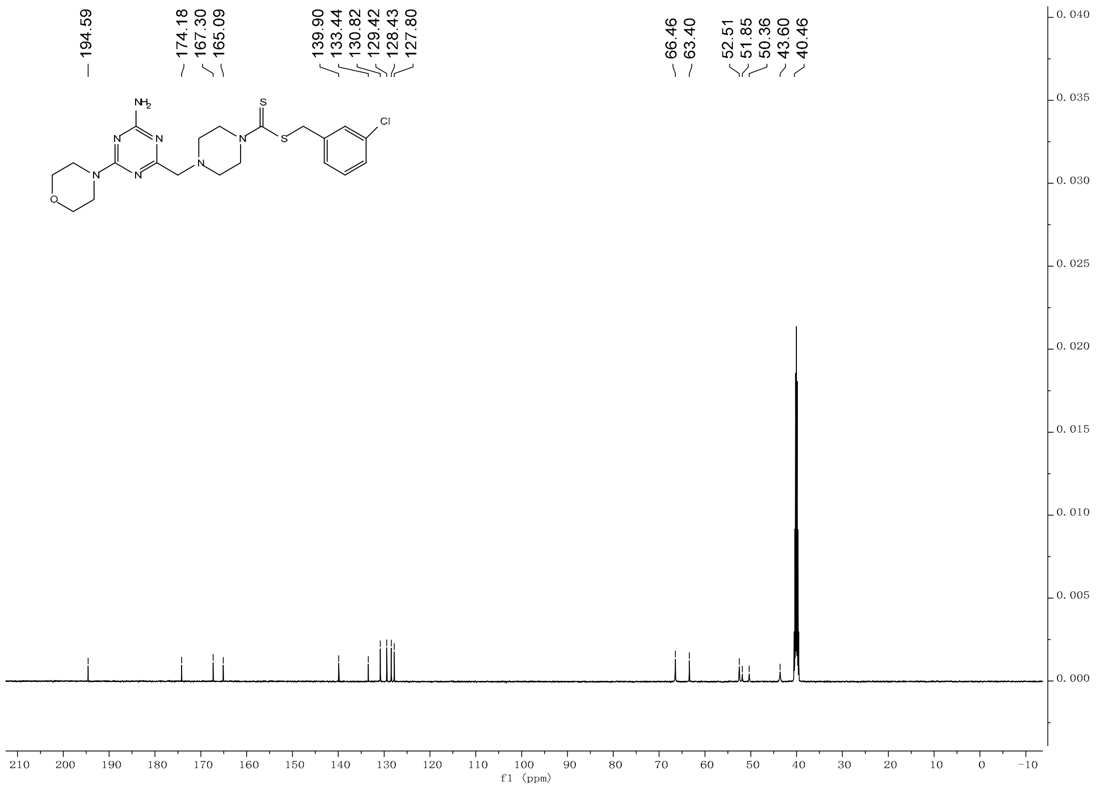

Figure S97. ^{13}C NMR (126 MHz, DMSO-d_6) spectrum of compound **C30**.

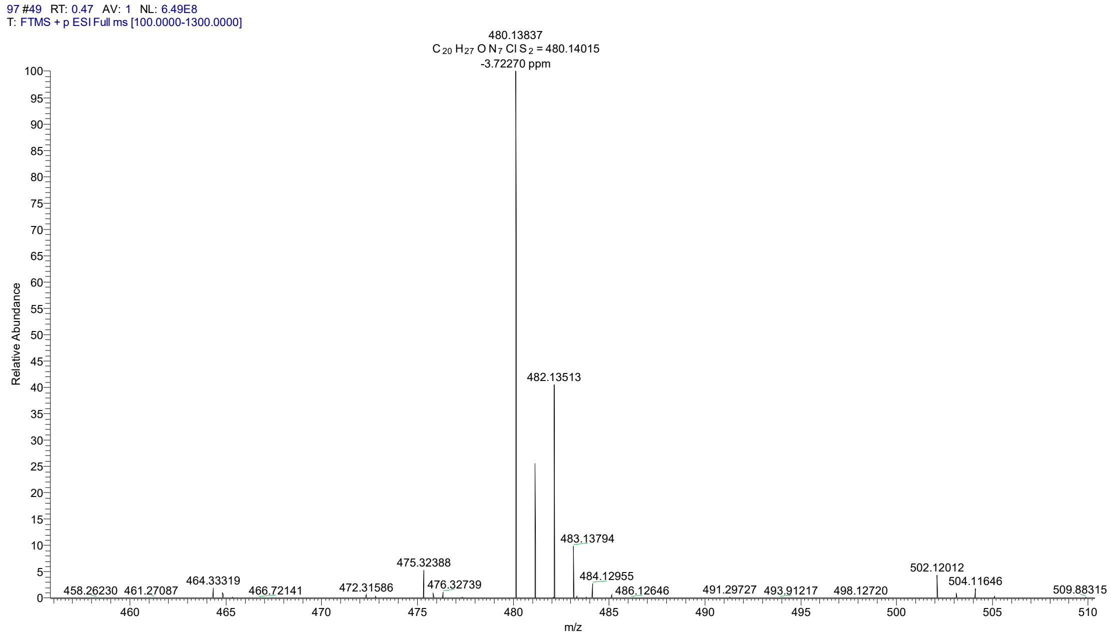

Figure S98. HRMS of compound **C30**.

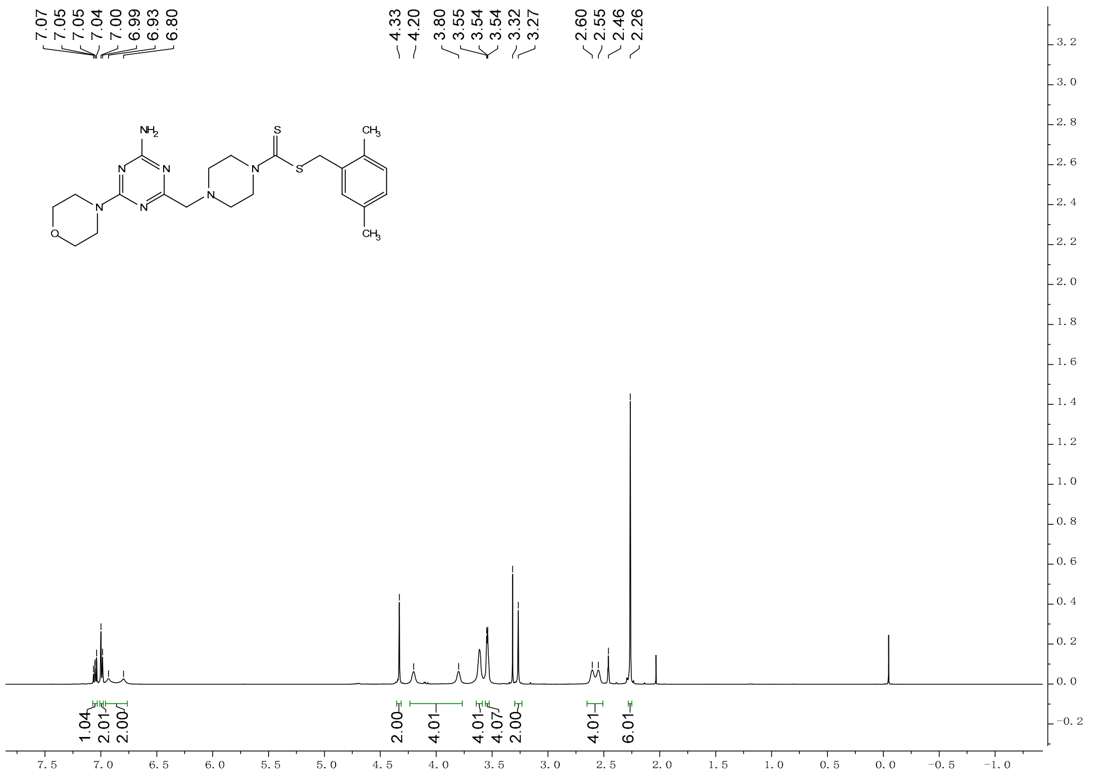

Figure S99. ^{1}H NMR (500 MHz, DMSO-d_6) spectrum of compound **C31**.

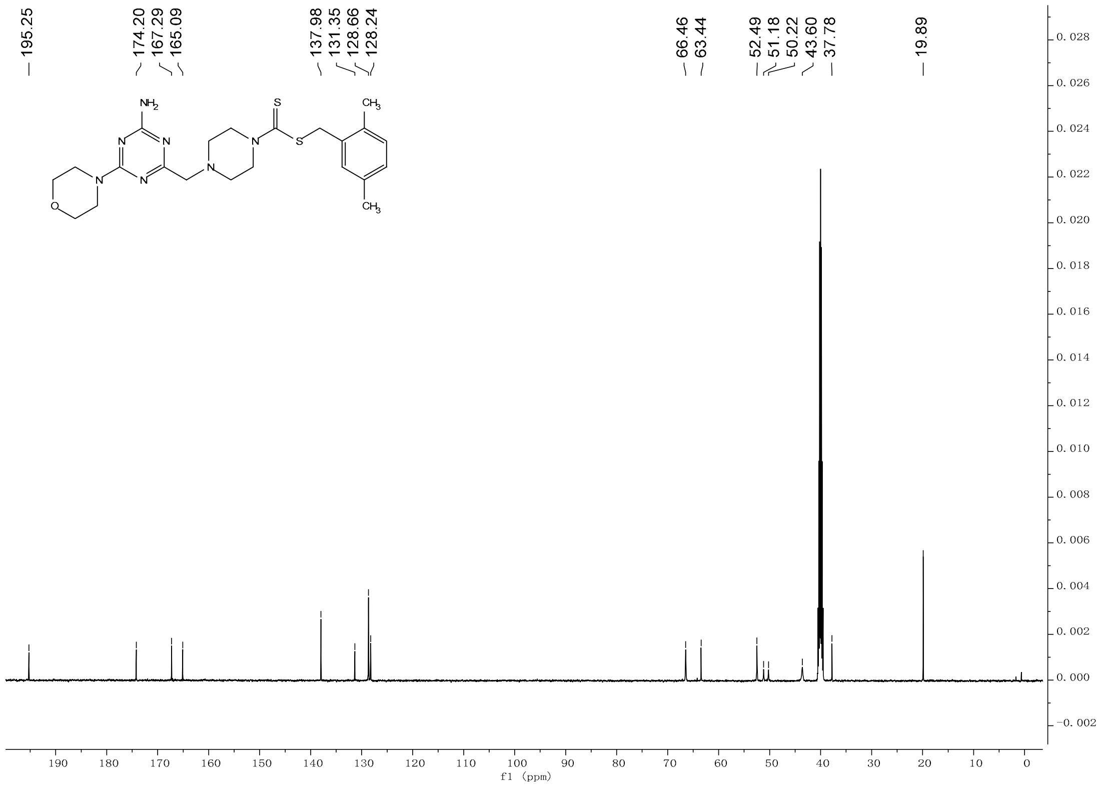

Figure S100. ^{13}C NMR (126 MHz, DMSO-d_6) spectrum of compound **C31**.

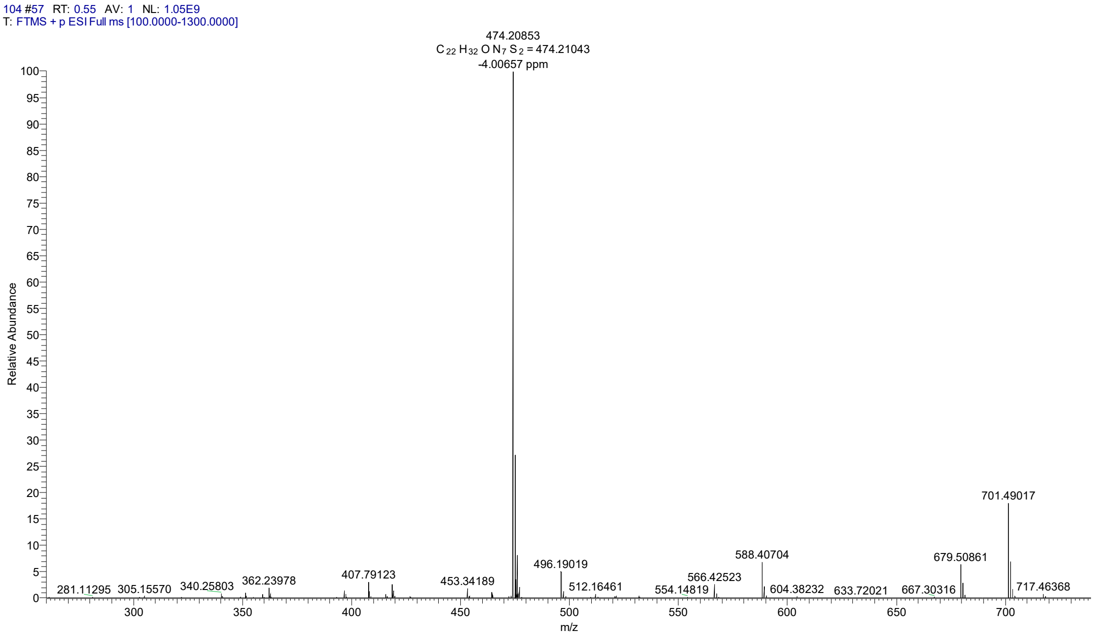

Figure S101. HRMS of compound **C31**.

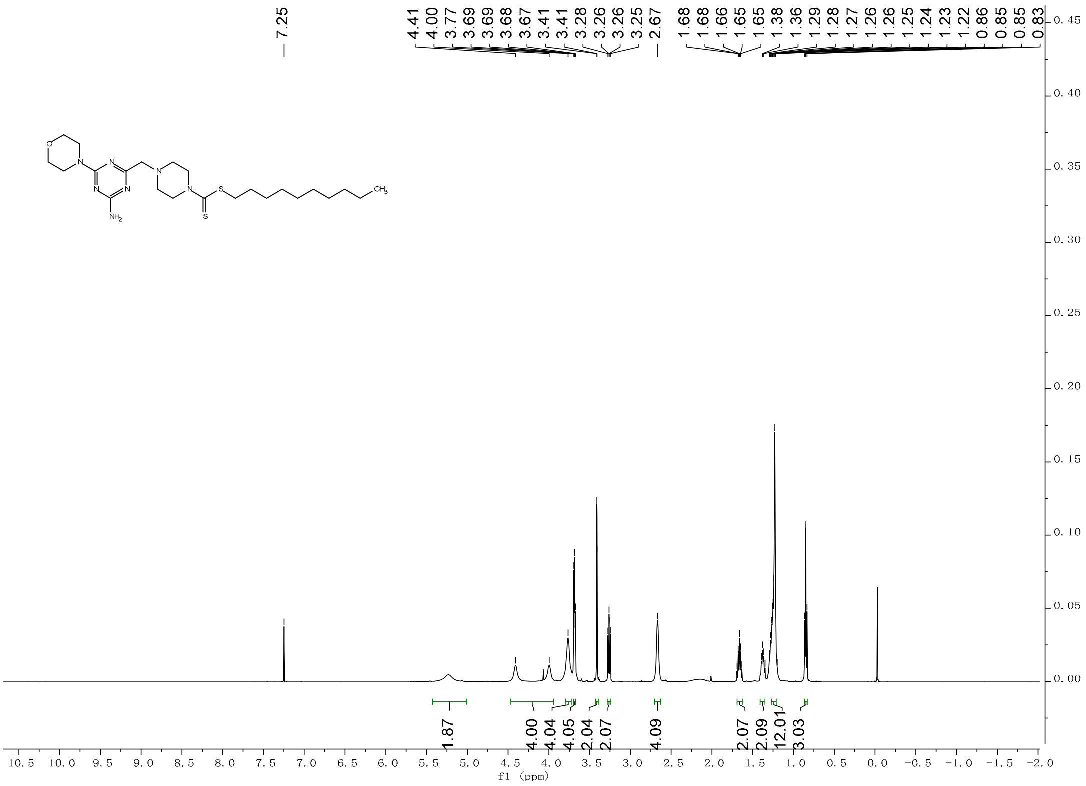

Figure S102. ^{1}H NMR (500 MHz, $CDCl_3$) spectrum of compound **C32**.

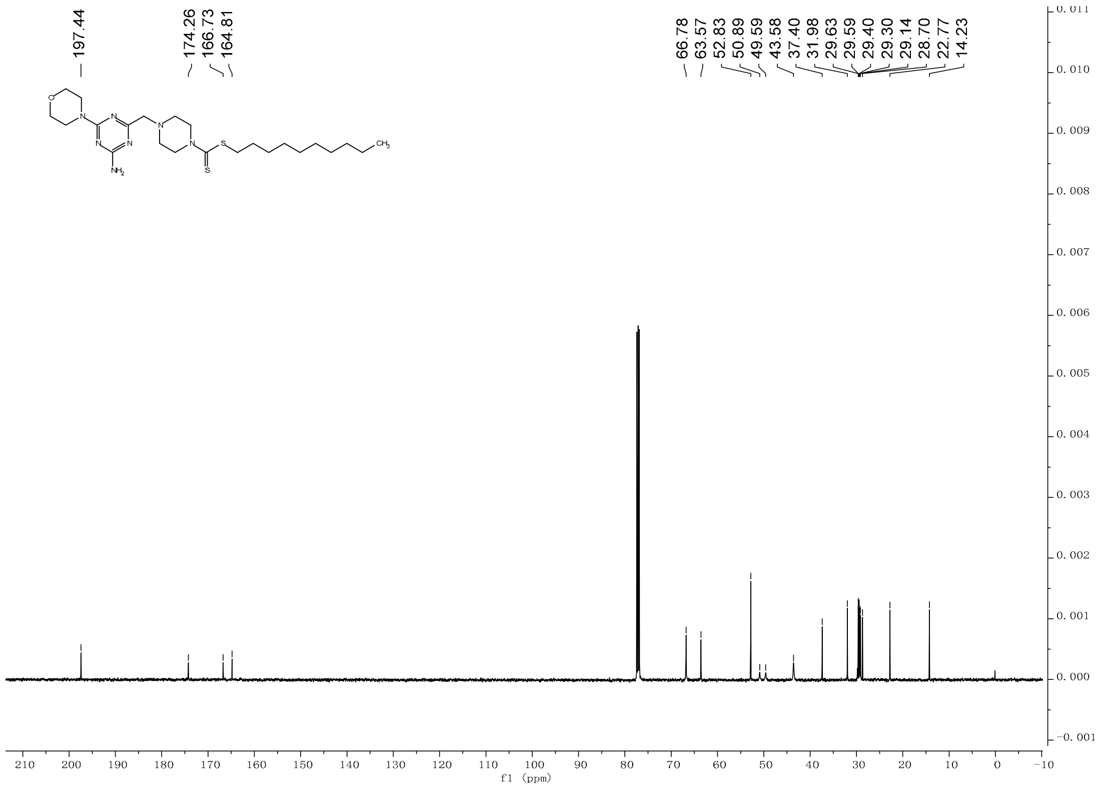

Figure S103. ^{13}C NMR (126 MHz, $CDCl_3$) spectrum of compound **C32**.

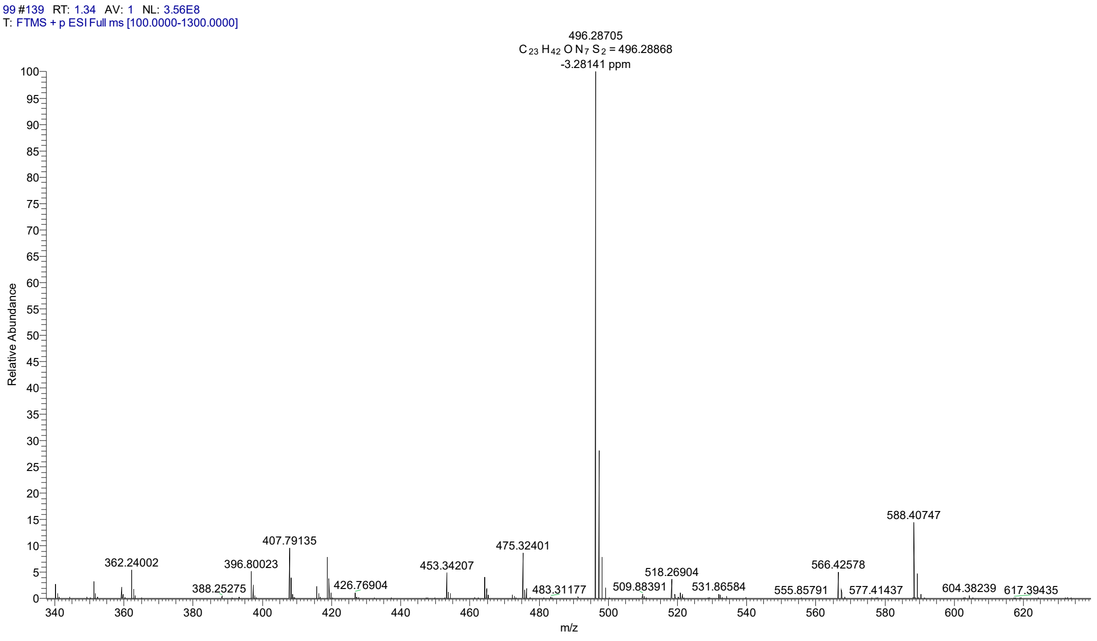

Figure S104. HRMS of compound **C32**.

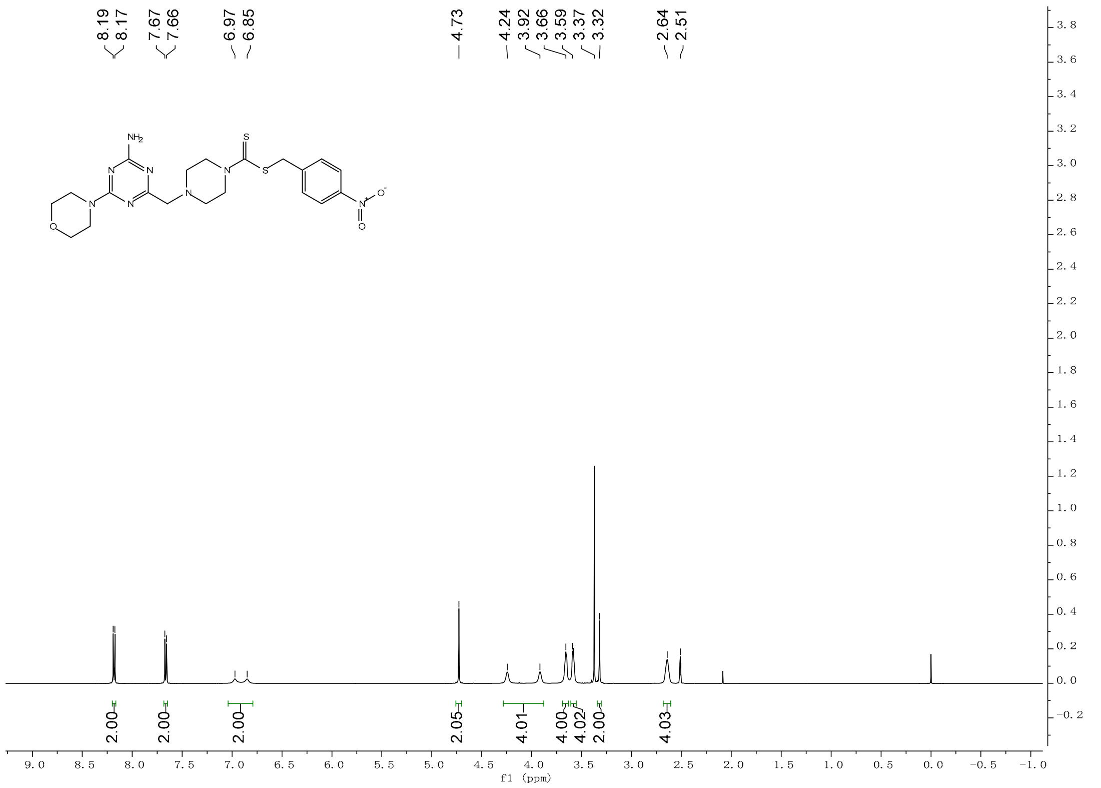

Figure S105. ^{1}H NMR (500 MHz, DMSO-d_6) spectrum of compound **C33**.

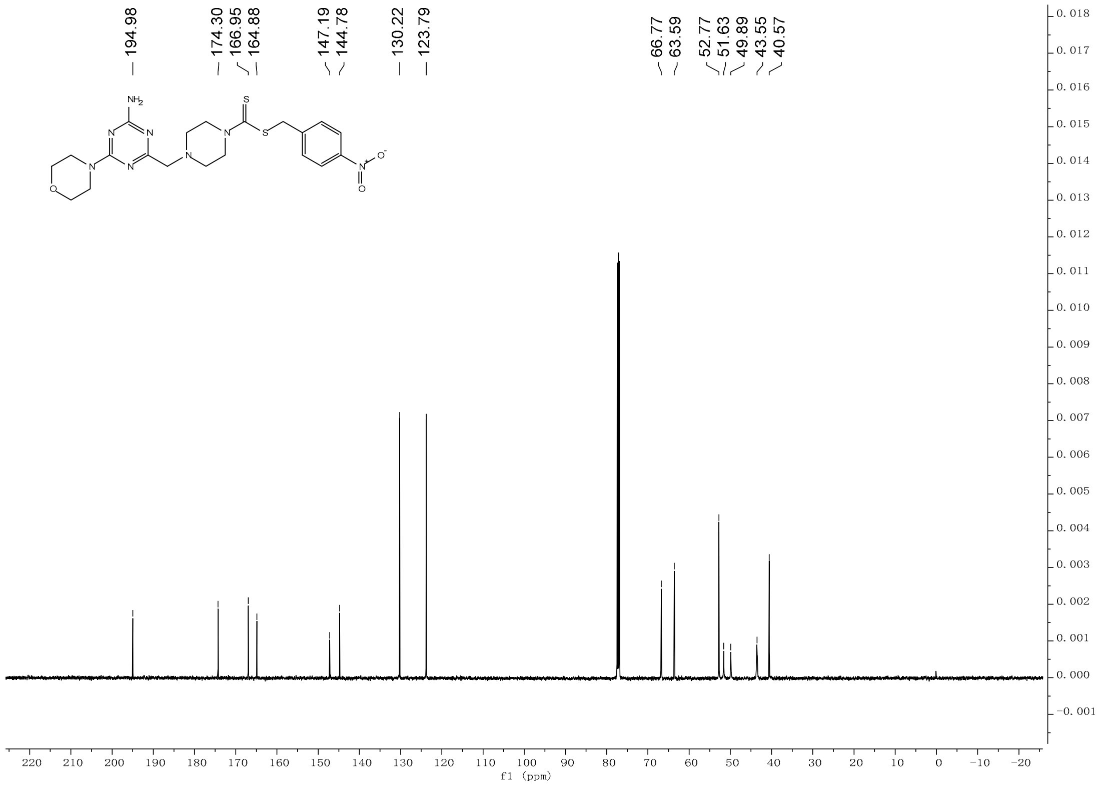

Figure S106. ^{13}C NMR (126 MHz, $CDCl_3$) spectrum of compound **C33**.

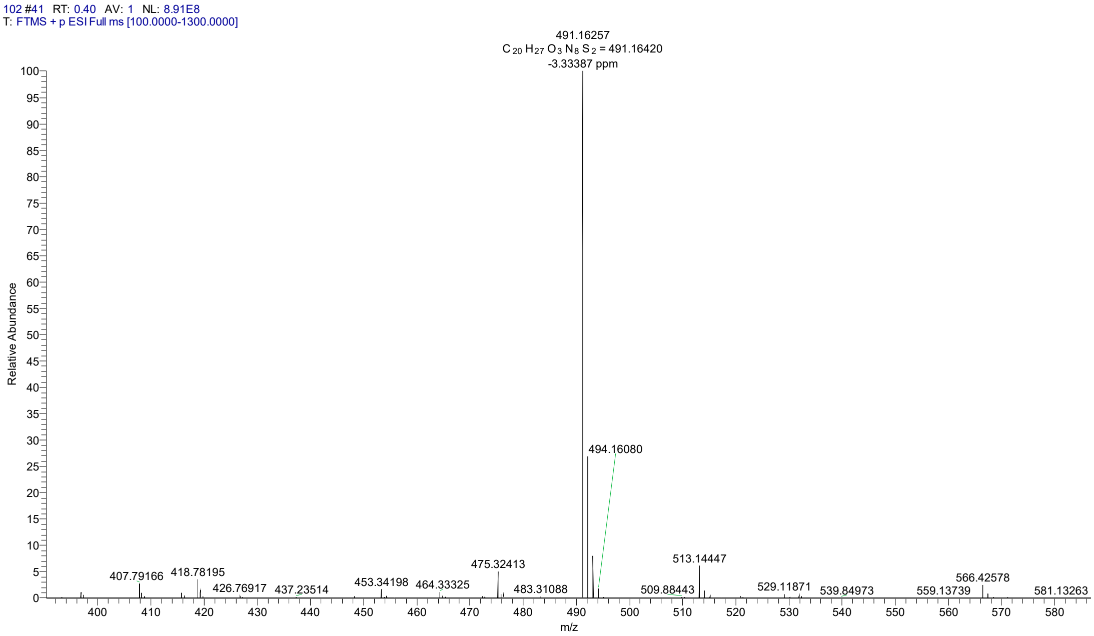

Figure S107. HRMS of compound **C33**.

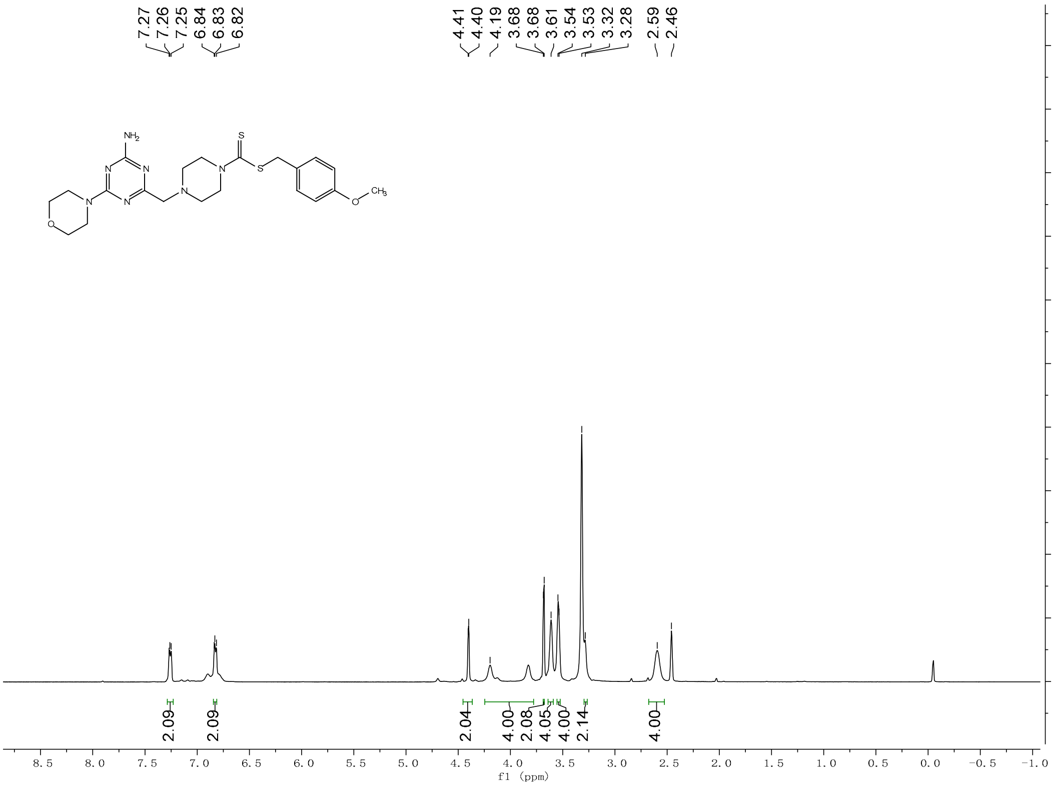

Figure S108. ^{1}H NMR (500 MHz, DMSO-d_6) spectrum of compound **C34**.

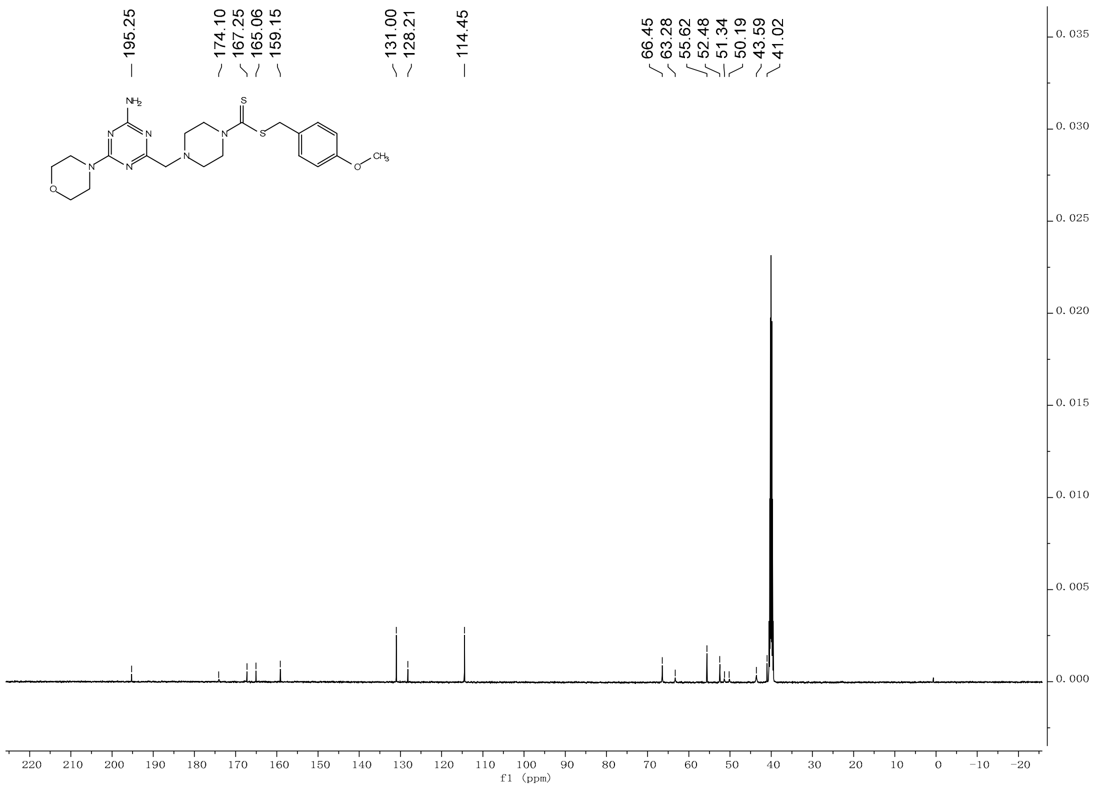

Figure S109. ^{13}C NMR (126 MHz, DMSO-d_6) spectrum of compound **C34**.

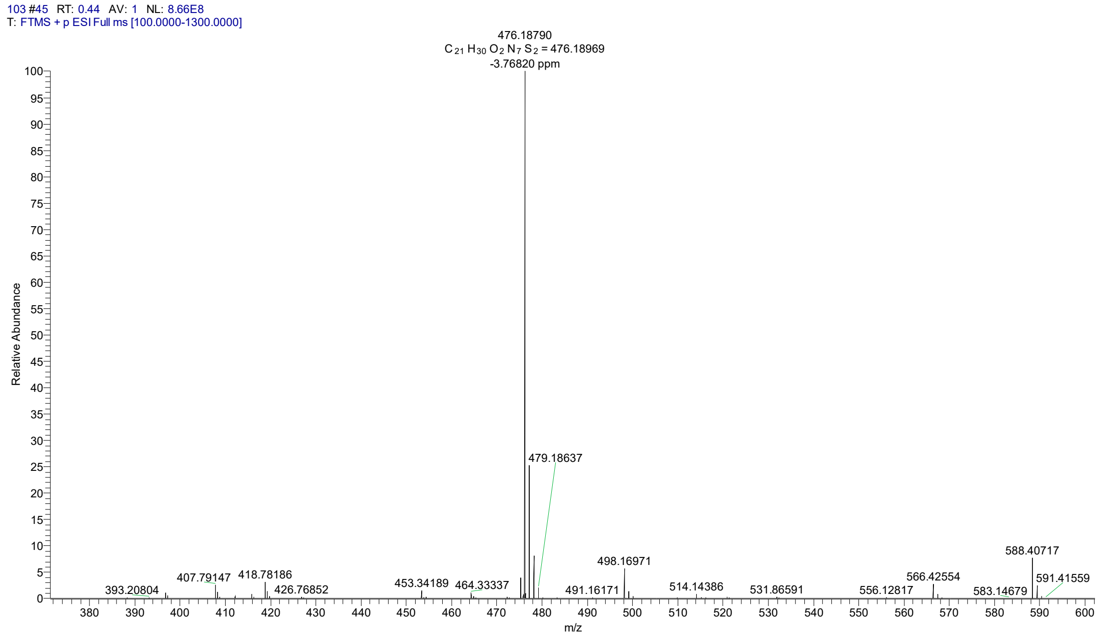

Figure S110. HRMS of compound **C34**.

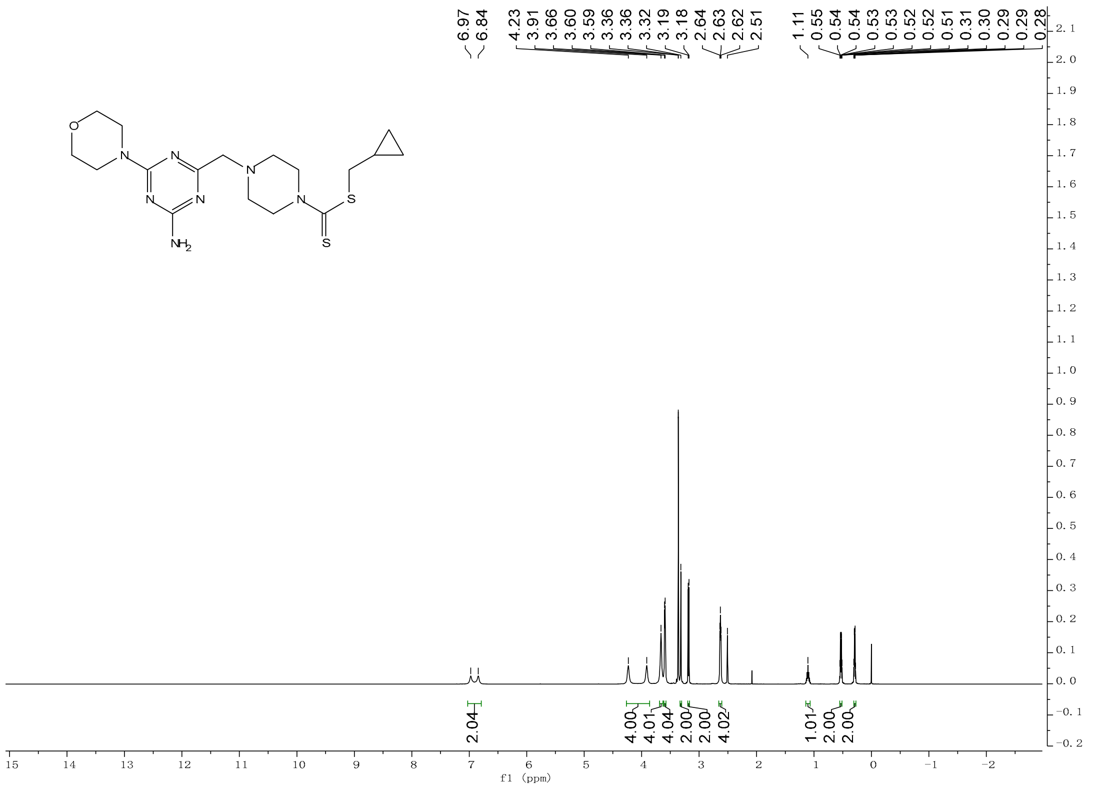

Figure S111. ^{1}H NMR (500 MHz, DMSO-d_6) spectrum of compound **C35**.

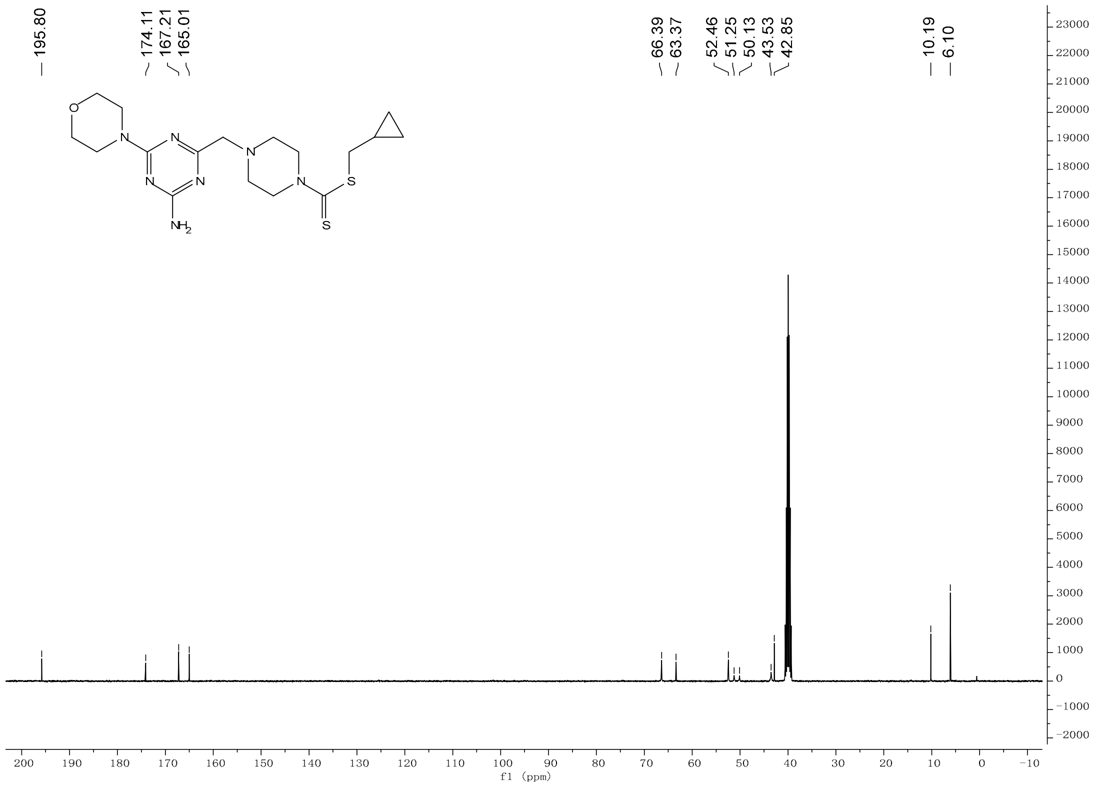

Figure S112. ^{13}C NMR (126 MHz, DMSO-d_6) spectrum of compound **C35**.

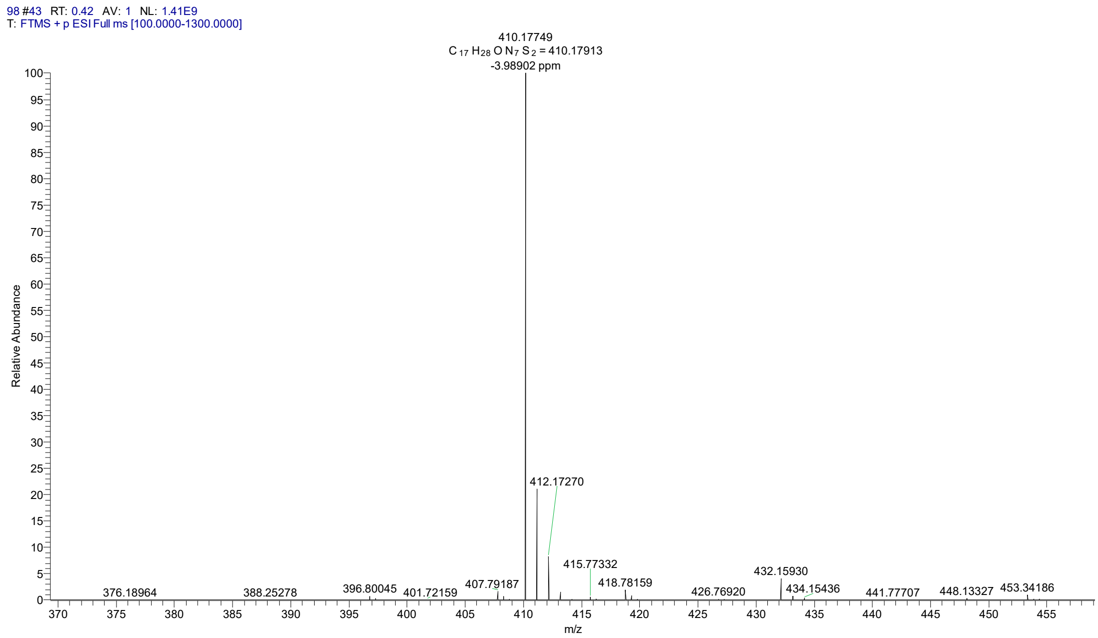

Figure S113. HRMS of compound **C35**.
